# Silver-mediated fluorodestannylation of an electron-rich thieno[3,2-*b*]thiophene diverts to oxygenation: an α-fluorothiophenone instead of an aryl fluoride

**DOI:** 10.3762/bjoc.22.124

**Published:** 2026-11-02

**Authors:** Hai-Tung Chuan, Li-Shin Shen, Huai-En Yu, Chia-Chi Chen, Jin-Pei Deng, Rong-Jiun Sheu, Chung-Shan Yu

**Affiliations:** 1 Department of Biomedical Engineering and Environmental Sciences, National Tsing Hua University, No. 101, Section 2, Kuang-Fu Road, Hsinchu 30013, Taiwan; 2 Department of Electronic Engineering, National Changhua University of Education, Changhua County 500207, Taiwan; 3 Department of Chemistry, Tamkang University, Tamsui, New Taipei City 251301, Taiwan; 4 Institute of Nuclear Engineering and Science, National Tsing Hua University, No. 101, Section 2, Kuang-Fu Road, Hsinchu 30013, Taiwan

**Keywords:** destannylation, electrophilic fluorination, fluorinated thiophene, organic semiconductors, thieno[3,2-*b*]thiophene

## Abstract

Fluorination of the terminal acceptor unit is a reliable way to deepen the LUMO level and enhance the electron mobility of quinoidal n-type organic semiconductors, yet no fluorinated analogue of the thieno[3,2-*b*]thiophene-fused acceptor TTC has been reported. Two routes to such an acceptor were examined. In a late-stage approach, the tricyclic 1,3-dione core was assembled first; under Pd(0)-catalyzed stannylation conditions, however, its doubly activated methylene group rather than the C–Br bond was engaged, as confirmed by a control experiment with a monoketone analogue. In an early-stage approach, a bicyclic thienothiophene stannane was subjected to silver-mediated fluorodestannylation with Selectfluor^TM^. Instead of the expected aryl fluoride, an α-fluorothiophenone was isolated and its structure established by HRMS and multinuclear NMR spectroscopy. Its formation is consistent with the interception of the aryl–silver intermediate by oxygen, followed by tautomerization and α-fluorination. In the absence of silver, the stannane underwent oxidative homocoupling without fluorine incorporation. These results reveal a limitation of silver-mediated fluorodestannylation on π-excessive fused thiophenes.

## Introduction

π-Conjugated organic semiconductors combine solution processability with frontier orbital energies that can be tuned by chemical modification [1–2]. Electron-transporting (n-type) materials nevertheless lag behind their hole-transporting counterparts, largely because a deep LUMO level and close, ordered π-stacking are difficult to achieve simultaneously when solubilizing alkyl chains are required [3]. Quinoidal (non-aromatic) cores address this conflict: they remove inter-ring torsional freedom, flatten the π-system and lower the LUMO, so that the rigid termini stack tightly even when the central donor carries solubilizing chains [4–6]. Among the terminal acceptors developed on this basis, 1,1-dicyanomethylene-3-indanone (IC) has become the benchmark; it is installed by Knoevenagel condensation and its acceptor strength can be tuned by substitution on the benzene ring [7–9].

Two such modifications are relevant here. First, fluorination of IC lowers the LUMO without the steric penalty of the heavier halogens: in a series of diketopyrrolopyrrole (DPP)-based small molecules, the fluorinated derivative showed more than doubled electron mobility of the parent compound (1.05 vs 0.44 cm^2^ V^−1^ s^−1^), whereas the chlorinated analogue fell to 0.26 cm^2^ V^−1^ s^−1^ [10]. Second, replacing the benzene ring of IC with thieno[3,2-*b*]thiophene gives the acceptor TTC, whose sulfur atoms promote short intermolecular S···S contacts and ordered stacking [11–13]. In organic solar cells, TTC raised the open-circuit voltage relative to IC, but at the cost of a shallower LUMO (−3.78 eV, against −4.00 eV for the difluorinated benzo analogue) and a lower short-circuit current [11]. A fluorinated TTC should therefore combine the packing advantages of the thienothiophene with the LUMO depth conferred by fluorine.

To our knowledge, no fluorinated TTC has been reported, reflecting the general difficulty of fluorinating thiophenes and their fused analogues [14–15]. Direct electrophilic fluorination with *N*-fluoro reagents is poorly regioselective or low-yielding [16–17], decarboxylative fluorination is restricted to electron-rich heteroarylcarboxylic acids [18], and organolithium-mediated fluorination with NFSI [15,19] is incompatible with the 1,3-dione of TTC. Fluorine must therefore be introduced through a pre-installed handle, most commonly by fluorodestannylation of an arylstannane, either uncatalyzed [20–21] or promoted by silver [22–23] or copper [24–26].

Here we compare a late-stage strategy, in which the tricyclic dione is assembled before fluorination, with an early-stage strategy in which the bicyclic thienothiophene is fluorinated first. The first route fails because the 1,3-dione, rather than the aryl bromide, is the reactive site towards palladium. The second fails for a more general reason: on this π-excessive fused thiophene, silver-mediated fluorodestannylation delivers an α-fluorothiophenone instead of an aryl fluoride, whereas in the absence of silver the stannane undergoes only oxidative homocoupling. An improved preparation of the complementary DPP donor block is also described.

## Results and Discussion

### Retrosynthetic analysis

**Scheme 1:**
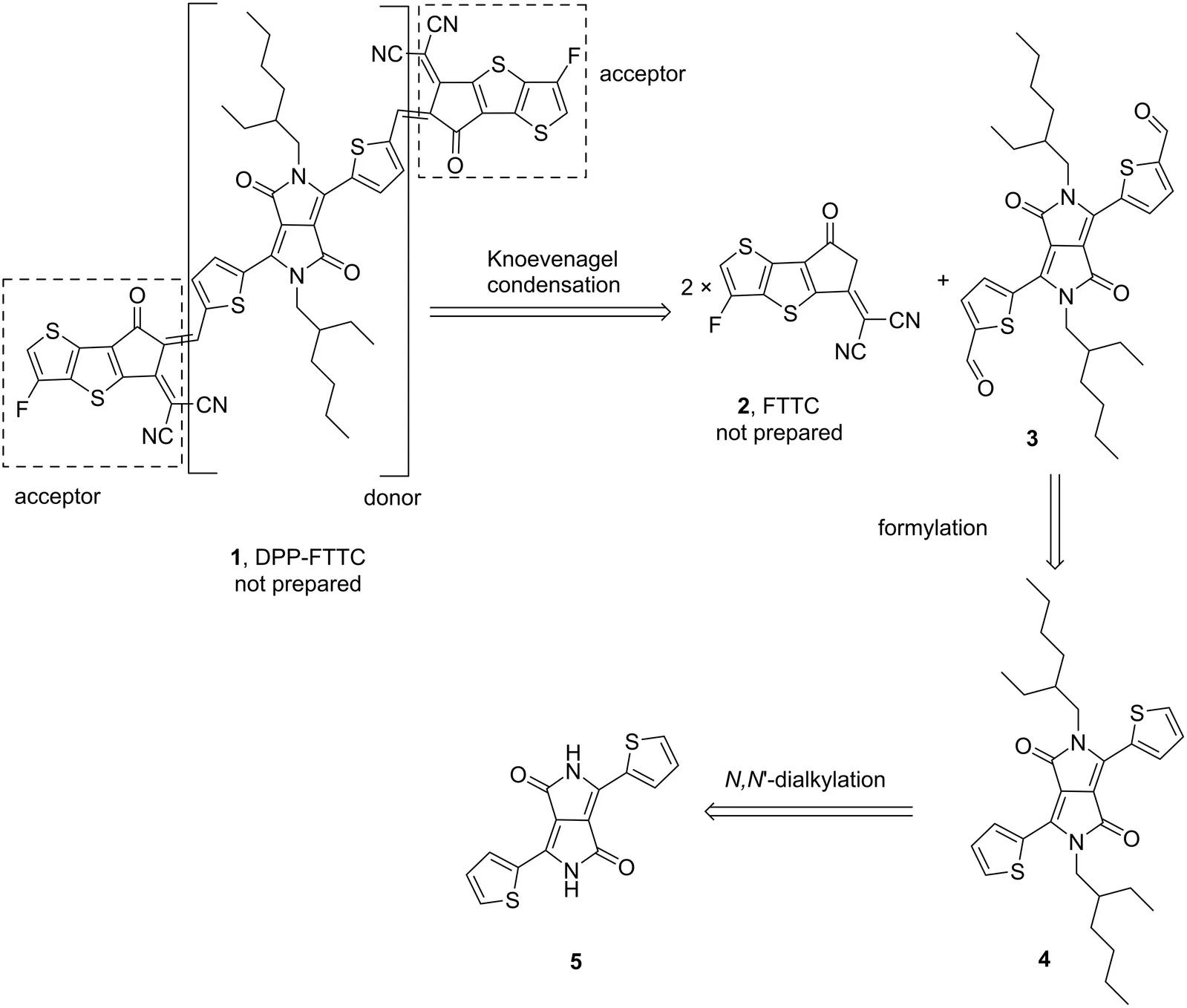
Retrosynthetic analysis of the target semiconductor **1** (DPP–FTTC). Fluorine is intended to deepen the LUMO of the TTC terminus while the fused thienothiophene preserves the S···S-directed packing that lowers the energy loss. Compounds **1**, **2**, and **12** are design targets and were not prepared in this work; no yields are given for them, and they are absent from the experimental section.

The target semiconductor **1** (DPP–FTTC) was designed to be assembled in the last step by double Knoevenagel condensation between the fluorinated acceptor **2** (FTTC) and bisaldehyde donor **3**, which is the standard approach for this class of quinoidal materials (Scheme 1) [9,27]. The donor **3** follows from *N*,*N*′-dialkylation of 3,6-di(thiophen-2-yl)-2,5-dihydropyrrolo[3,4-*c*]pyrrole-1,4-dione (**5**, DTPP) to **4** and subsequent formylation of the thiophene α-positions. The acceptor **2** follows from malononitrile condensation on the fluorinated tricyclic dione **12**. Compounds **1**, **2**, and **12** define the synthetic objective and were not obtained. The following discussion addresses the reasons for this.

**Scheme 2:**
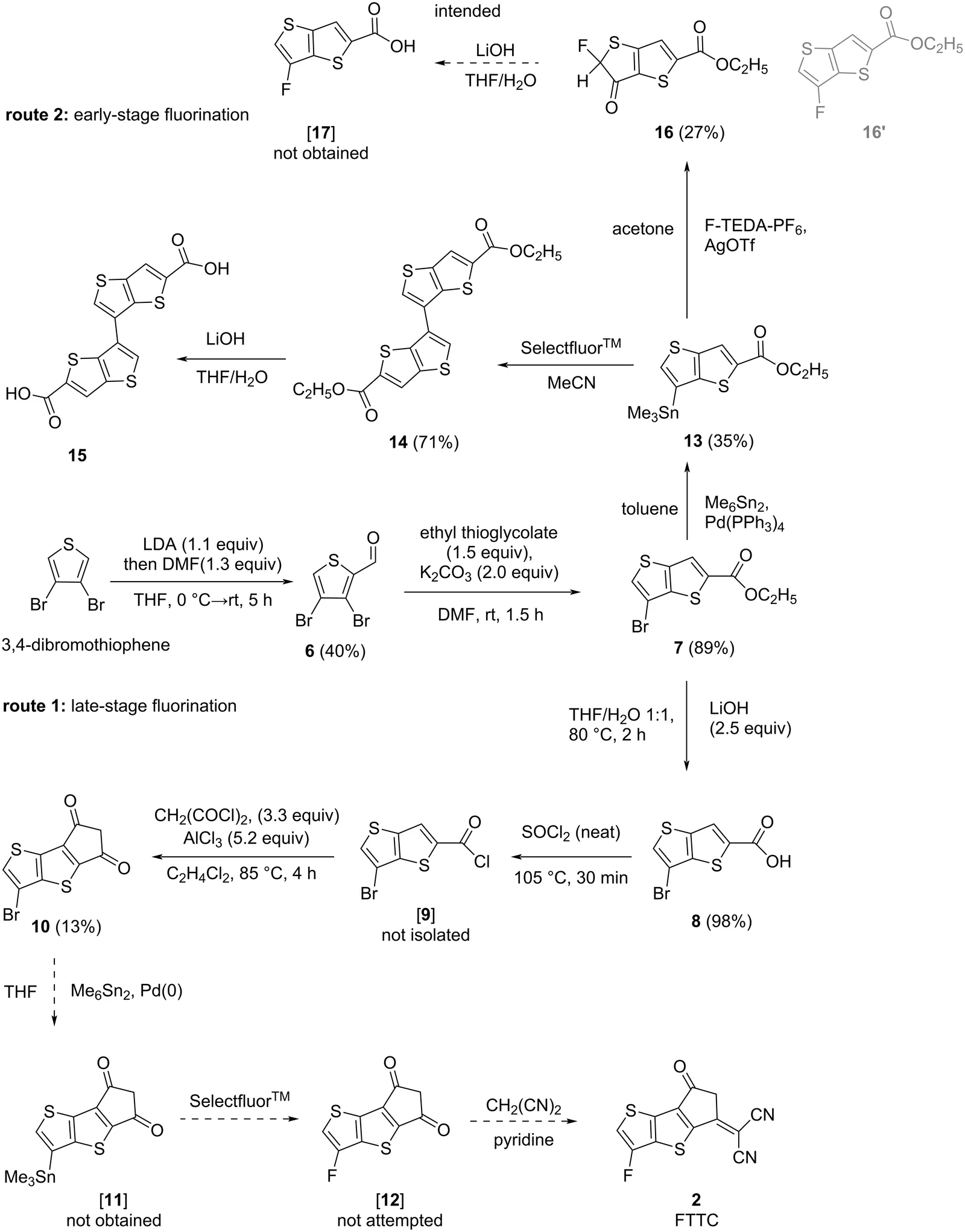
The two strategies examined for installing fluorine on the cyclopenta[*b*]thieno[2,3-*d*]thiophene-5,7(6*H*)-dione core. Dashed arrows denote transformations that did not give the intended product or were not attempted. Acid **17** is the intermediate targeted by the original design, in which fluorine was expected at carbon-6 of the aryl fluoride (**16’**). Compound **16’** could not be obtained, because the reaction actually formed **16**, which carries fluorine on an sp^3^ carbon and decomposes under the hydrolysis conditions.

All of the synthetic difficulty resides in **12**. Its carbon skeleton is built by an intramolecular Friedel–Crafts acylation that closes the cyclopentane-1,3-dione ring onto a thieno[3,2-*b*]thiophene, so the fluorine atom can be introduced either before ring closure, on the bicyclic thienothiophene, or after it, on the assembled tricycle. Although 3-bromo-4-fluorothiophene is offered by a few suppliers, it is not a stock building block, and its preparation from 3,4-dibromothiophene requires silyl protection of both α-positions followed by lithiation with excess *t*-BuLi and fluorination with NFSI [19]. We therefore elected to carry a bromine atom through the sequence as a fluorine surrogate and to compare the two points of exchange (Scheme 2).

### Late-stage fluorination (route 1): assembly of the tricyclic dione **10**

3,4-Dibromothiophene was formylated at C2 with freshly prepared LDA followed by DMF. Deprotonation at −78 °C returned unchanged starting material; when performing the reaction at 0 °C the aldehyde **6** was isolated in 40%, and once the *n*-BuLi titre had been corrected the yield rose to 74%, approaching the 80–90% reported previously [28–29]. Exceeding 1.1 equiv of LDA generated two more polar by-products and eroded the yield, so the base stoichiometry is the controlling variable. Annulation with ethyl thioglycolate then furnished ethyl 6-bromothieno[3,2-*b*]thiophene-2-carboxylate (**7**) in 89% [30], and LiOH-mediated ester hydrolysis gave the acid **8** in 98% (Scheme 2).

Closure of the third ring proved to be the bottleneck. Acid **8** is insoluble in chlorinated solvents, so activation was carried out with SOCl_2_ using catalytic DMF; above one equivalent of DMF the resulting salts could not be removed under reduced pressure and the crude acid chloride **9** fumed on contact with air, whereas 0.4 equiv left a tractable residue [31]. Using neat SOCl_2_ as both reagent and solvent was equally effective. Because the acid chloride **9** is highly moisture-sensitive it was taken directly into the AlCl_3_-promoted Friedel–Crafts acylation with malonyl chloride. Under all conditions examined the tricyclic dione **10** was obtained in no more than 17%, well below the 52% reported for the parent system [11] (Table 1, entries 1–4).

**Table 1:** Optimization of the acid chloride formation and Friedel–Crafts annulation.^a^

entry	substrate	solvent	DMF (equiv)	AlCl₃ (equiv)	activated with^b^	product	yield (%)

1	**8**	CHCl_3_^c^	0.3	6.0	aroyl chloride	**10**	10–17
2	**8**	CHCl_3_^c^	1.2	6.0	aroyl chloride	**10**	0–5
3	**8**	CH_2_Cl_2_	0.4	2.3	malonyl chloride	**10**	12
4	**8**	DCE	0.4	2.3	malonyl chloride	**10**	13
5^d^	benzoyl chloride	CH_2_Cl₂	–	3.0	aroyl chloride	indan-1,3-dione	7
6	benzoyl chloride	CH_2_Cl_2_	–	3.0	aroyl chloride	indan-1,3-dione	10
7	benzoyl chloride	CH_2_Cl_2_	–	2.3	malonyl chloride	indan-1,3-dione	24
8	benzoyl chloride	DCE	–	2.3	malonyl chloride	indan-1,3-dione	45

^a^Malonyl chloride (4.0–4.5 equiv), reflux, 12–16 h. DCE = 1,2-dichloroethane. Full details in Supporting Information File 1, Tables S1 and S2. ^b^The acyl chloride pre-stirred with AlCl_3_ at 0 °C for 15 min before addition of the second component. ^c^CHCl_3_ stabilized with 1% ethanol gave <5% together with an ethyl ester by-product. ^d^Malonyl chloride (1.3 equiv).

To establish whether the low yield was intrinsic to the electron-rich thienothiophene or to the reaction conditions, benzoyl chloride was used as a model substrate for the same annulation to indan-1,3-dione. Two changes proved decisive (Table 1, entries 5–8): pre-activating malonyl chloride, rather than the aroyl chloride, with AlCl_3_, and raising the reaction temperature. Published preparations of indan-1,3-diones are run in nitrobenzene at 80–140 °C [32–33]. Since nitrobenzene is a controlled toxic substance in our jurisdiction, we replaced it with 1,2-dichloroethane (bp 83.5 °C), which allowed the same temperature window to be reached and delivered indan-1,3-dione in 41–45%, within the reported 33–50% range [32–33]. This is a useful and less hazardous variant of a widely used transformation. Applied to compound **9**, however, the optimized protocol gave dione **10** in only 13%, indicating that the limitation is substrate-intrinsic rather than procedural. The four isolated operations therefore deliver **10** in 5% overall yield from 3,4-dibromothiophene (40%, 89%, 98%, 13%) and when carrying out the formylation with corrected *n*-BuLi titre, the overall yield raises to 8%.

### The 1,3-dione blocks stannylation

With dione **10** in hand, conversion of the C3-bromide into a stannane was attempted as the gateway to fluorination. Two standard protocols were used: Me_6_Sn_2_ with Pd(PPh_3_)_2_Cl_2_ in 1,4-dioxane and Me_6_Sn_2_ with Pd(PPh_3_)_4_ in refluxing THF [34–35]. In both cases the starting material was consumed within one hour, but the product was not the expected arylstannane **11**.

The ^1^H NMR spectra of the two crude products were identical and diagnostic. The two-proton singlet of the C6 methylene at δ 3.4 ppm had disappeared and was replaced by a one-proton multiplet at δ 4.2 ppm, while the single aromatic resonance shifted downfield from δ 7.7 to δ 8.1 ppm. The isolated material was a brown oil carrying the characteristic sweet odor of an alkyltin compound, and the bright yellow fluorescence of **10** was lost. Taken together these observations indicate that the reaction has occurred at the doubly activated C6 position – the methylene flanked by both carbonyls, whose p*K*_a_ is comparable to that of a 1,3-diketone – rather than at the C–Br bond, converting the CH_2_ group into a monosubstituted CH center. The C–Br bond survives the reaction, and the aryl bromide is simply not the most reactive electrophilic site in this substrate.

**Scheme 3:**
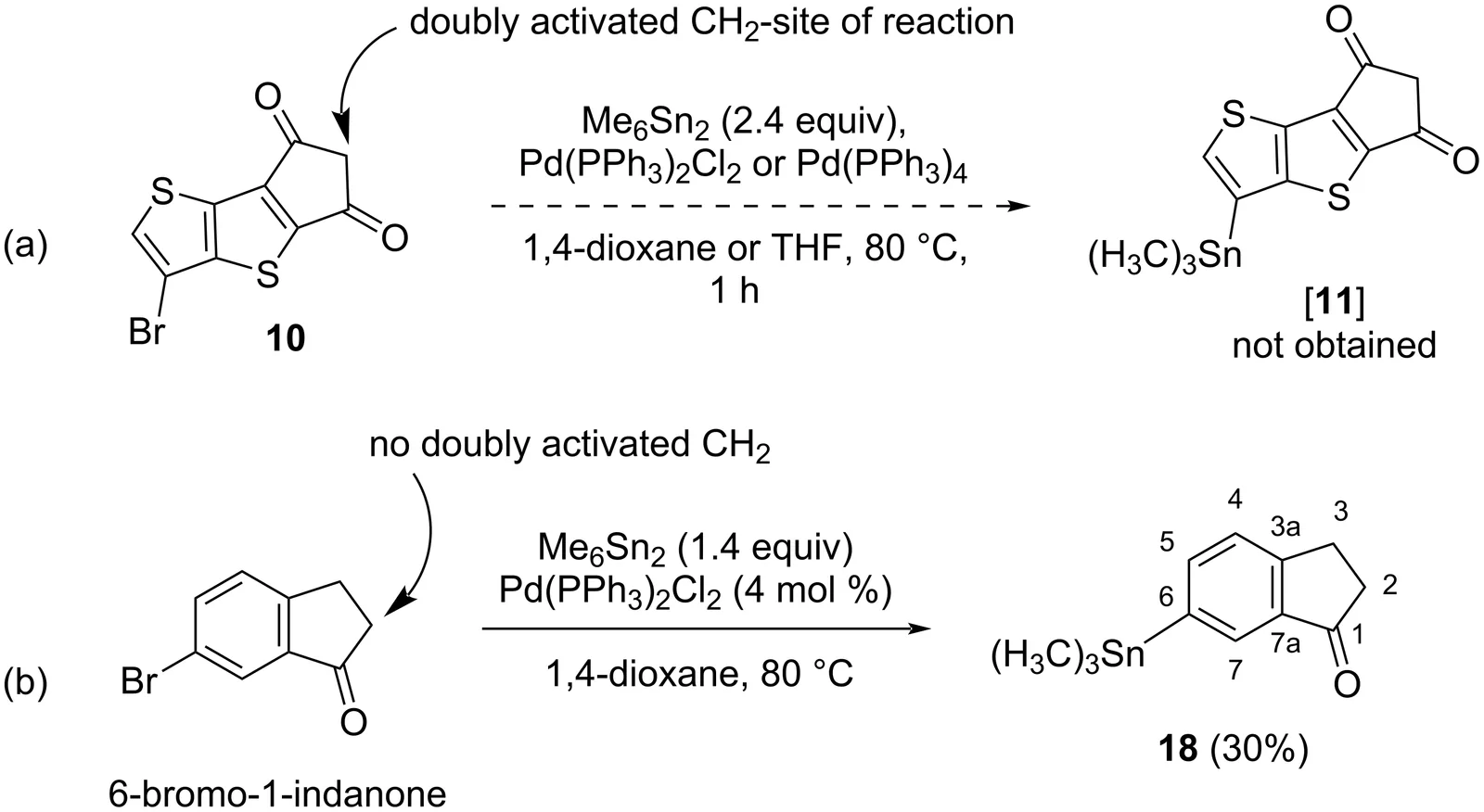
(a) Attempted stannylation of **10**, in which the doubly activated C6 methylene rather than the C3–Br bond is engaged; (b) the control reaction with 6-bromo-1-indanone, which lacks a doubly activated methylene and reacts as expected.

That the failure is specific to the dione was confirmed with a control substrate. 6-Bromo-1-indanone, which carries a single carbonyl and therefore no doubly activated methylene, underwent stannylation cleanly under the same conditions to give the corresponding arylstannane **18** in 30%, identified by its ^1^H NMR spectrum, which shows the expected ^117/119^Sn satellites, a triplet at δ 7.9 ppm (*J* = 21.5 Hz) for H7 and a triplet of doublets at δ 7.7 ppm (*J* = 19.5, 7.5 Hz) for H5, together with the trimethyltin signal at δ 0.3 ppm (Scheme 3).

The practical conclusion is that any late-stage functionalization of this acceptor core must either avoid organometallic conditions altogether or mask the 1,3-dione first.

### Early-stage fluorination (route 2): the stannane and the role of silver

Relocating the halogen exchange to the bicyclic stage removes the 1,3-dione from the substrate entirely. Stannylation of **7** with Me_6_Sn_2_/Pd(PPh_3_)_2_Cl_2_ in 1,4-dioxane gave the trimethylstannane **13** in ca. 21% yield accompanied by 0.25 equiv of the protodestannylated arene, ethyl thieno[3,2-*b*]thiophene-2-carboxylate. Switching to Pd(PPh_3_)_4_ in toluene and adding 3% Et_3_N to the eluent to suppress the decomposition of stannanes on silica gel [36] reduced this by-product to 0.1 equiv and, with 2.3 equiv of Me_6_Sn_2_, raised the yield of **13** to 35% (by ^1^H NMR). The reaction must be stopped when the solution darkens as prolonged heating consumes both **7** and **13** and generates a less polar by-product. Because **7** and **13** are nearly isopolar they could not be separated by flash chromatography, but this proved immaterial, as **7** is inert to the subsequent fluorination and is recovered unchanged.

**Scheme 4:**
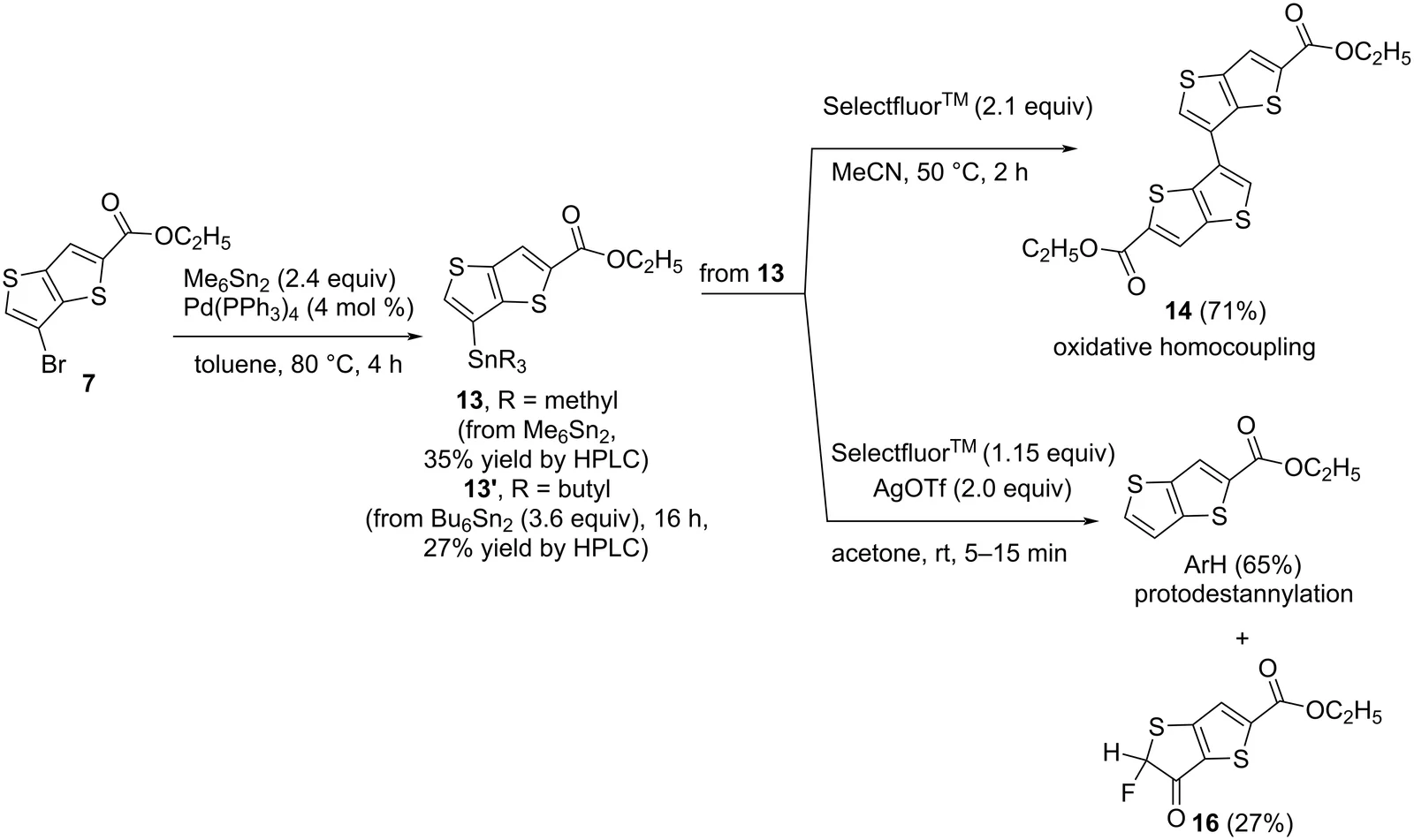
Stannylation of **7** and the divergent behavior of the resulting stannane towards Selectfluor^TM^ in the absence and presence of silver.

The stannane then follows one of two oxidative pathways, and which one operates is decided by a single additive (Scheme 4). Adding silver changes the outcome completely. Following Ritter's protocol [22–23], stannane **13** was treated in acetone with AgOTf generated in situ from Ag_2_O and TfOH, then with Selectfluor^TM^. The reaction was complete within 5–15 min and gave a single fluorine-containing product in 27% yield, with protodestannylation as the major competing pathway (65%) and only ca. 5% of dimer **14** (Table 2, entry 1). Transmetalation of the stannane to a transient aryl–silver species evidently outcompetes the oxidative dimerization. That product, however, is not the aryl fluoride **16’** that this chemistry delivers on benzenoid substrates.

**Table 2:** Silver-mediated fluorination of the stannanes **13** and **13’**.^a^

entry	stannane	fluorinating agent (equiv)	AgOTf (equiv)	NaHCO_3_ (equiv)	time	*T*	ArH (%)^b^	**16** (%)^b^

1	**13** (SnMe_3_)	Selectfluor^TM^ (1.15)	2.0	–	5–15 min	rt	65	27
2	**13’** (SnBu_3_)	Selectfluor ^TM^ (1.2)	2.0	–	30 min	rt	80	13
3	**13**’ (SnBu_3_)	F-TEDA-PF_6_ (1.2)	2.0	–	1 h	rt	70	15
4	**13**’ (SnBu_3_)	F-TEDA-PF_6_ (1.5)	0.1	2.0	30 min	reflux	n.d.	n.d.
5	**13**’ (SnBu_3_)	F-TEDA-PF_6_ (1.5)	0.1	2.0	30 min	rt	40	15
6	**13**’ (SnBu_3_)	F-TEDA-PF_6_ (1.15)	1.5	1.1	1 h	0 °C	82	16

^a^Acetone, 5 min to 1 h. AgOTf generated in situ from Ag_2_O and TfOH. F-TEDA-PF_6_ is the hexafluorophosphate salt of Selectfluor^TM^. n.d. = not detected. ^b^Determined by ^1^H NMR of the crude mixture. ArH = ethyl thieno[3,2-*b*]thiophene-2-carboxylate, the protodestannylation product. Isolated yields are given in Supporting Information File 1.

Attempts to improve the yield beyond 27% were unsuccessful, and the pattern of failure is itself informative (Table 2). The tributylstannane **13’**, prepared from the less expensive Bu_6_Sn_2_, gave the fluorinated product in 13–16% under every condition examined, well below the 27% obtained with **13**. Exchanging Selectfluor^TM^ for the more soluble F-TEDA-PF_6_ [22] made no difference (Table 2, entries 2 and 3). What did change was the extent of protodestannylation, and it tracked the silver loading rather than the added base. Reducing AgOTf from 2.0 to 0.1 equiv in the presence of NaHCO_3_ [23] cut the yield of the protodestannylated arene from 70–80% to 40% (Table 2, entries 3 and 5), whereas adding the same base while keeping the silver loading high left protodestannylation at 82%, the highest value in the series (Table 2, entry 6). Suppressing the competing pathway therefore does not liberate the fluorination: across a twofold change in the amount of protodestannylated arene formed, the yield of **16** stays fixed near 15%. Raising the temperature destroys both – at reflux neither product could be detected (Table 2, entry 4).

### The product is an α-fluorothiophenone, not an aryl fluoride

The molecular formula settles the question first. No ion corresponding to the aryl fluoride C_9_H_7_FO_2_S_2_ (*m/z* 229.9871) was detected in the mass spectrum under EI. The molecular ion observed at *m/z* 245.9822 matches C_9_H_7_FO_3_S_2_ (calcd for 245.9821, Δ = 0.4 ppm) with an isotope pattern consistent with two sulfur atoms: the product carries one oxygen atom more than the aryl fluoride, with the same number of hydrogens and, since neither hydrogen nor fluorine count changes, the same six degrees of unsaturation. An oxygen can be added under that constraint only by converting a C=C into a C=O, or by oxidizing sulfur.

The hydrogen count then fixes where that oxygen sits. Of the seven hydrogens, five belong to the ethyl ester, leaving two on the ring system – the same number as in the stannane **13**, which carries H at C3 and C5 and SnMe_3_ at C6. Since fluorine and one of those hydrogens are mutually coupled and therefore share a carbon, fluorine cannot be at C6. But C6 must acquire some substituent on losing tin, and the only atom available is oxygen. Oxygen therefore occupies C6 and fluorine occupies C5.

The ^13^C NMR spectrum then establishes the connectivity directly, without recourse to inference. The carbon at δ 98.2 ppm is a protonated carbon by DEPT and appears as a doublet with a one-bond coupling of 232.1 Hz. A carbon that carries a hydrogen and is simultaneously bonded to fluorine can only be an sp^3^ C(H)F unit, and the coupling constant is exactly what that unit requires here. One-bond C–F couplings rise steeply as electronegative substituents accumulate on the fluorinated carbon from 158 Hz in CH_3_F to about 235 Hz in CH_2_F_2_ and C5 bears, in addition to hydrogen and fluorine, both a ring sulfur and an adjacent carbonyl. An aromatic C–F carbon would show a comparable one-bond coupling, but it would be quaternary and would resonate near δ 158 ppm rather than at δ 98 ppm. Conversely, an aromatic CH *ortho* to fluorine at δ 98 ppm would show a two-bond coupling of 20–27 Hz, an order of magnitude smaller than observed.

The ^1^H and ^19^F NMR spectra corroborate this independently. The proton attached to this carbon resonates at δ 6.28 ppm with *J*(H,F) = 52.5 Hz, the value expected for a two-bond coupling within an H–C–F unit, and the ^19^F resonance at δ −167.9 ppm carries the same splitting. Both observables exclude the aromatic alternative: in 3-fluorothiophene no coupling from fluorine to a ring proton exceeds 3.4 Hz and the ^19^F resonance lies at δ −131.0 ppm [37–38]. They also exclude a vinylic fluorine, for which δ_F_ would fall between −90 ppm and −130 ppm, and which in any case could not give 52.5 Hz within a five-membered ring, where the substituents projecting from an endocyclic C=C are necessarily *cis* (^3^*J* ≈ 20 Hz).

**Figure 1:**
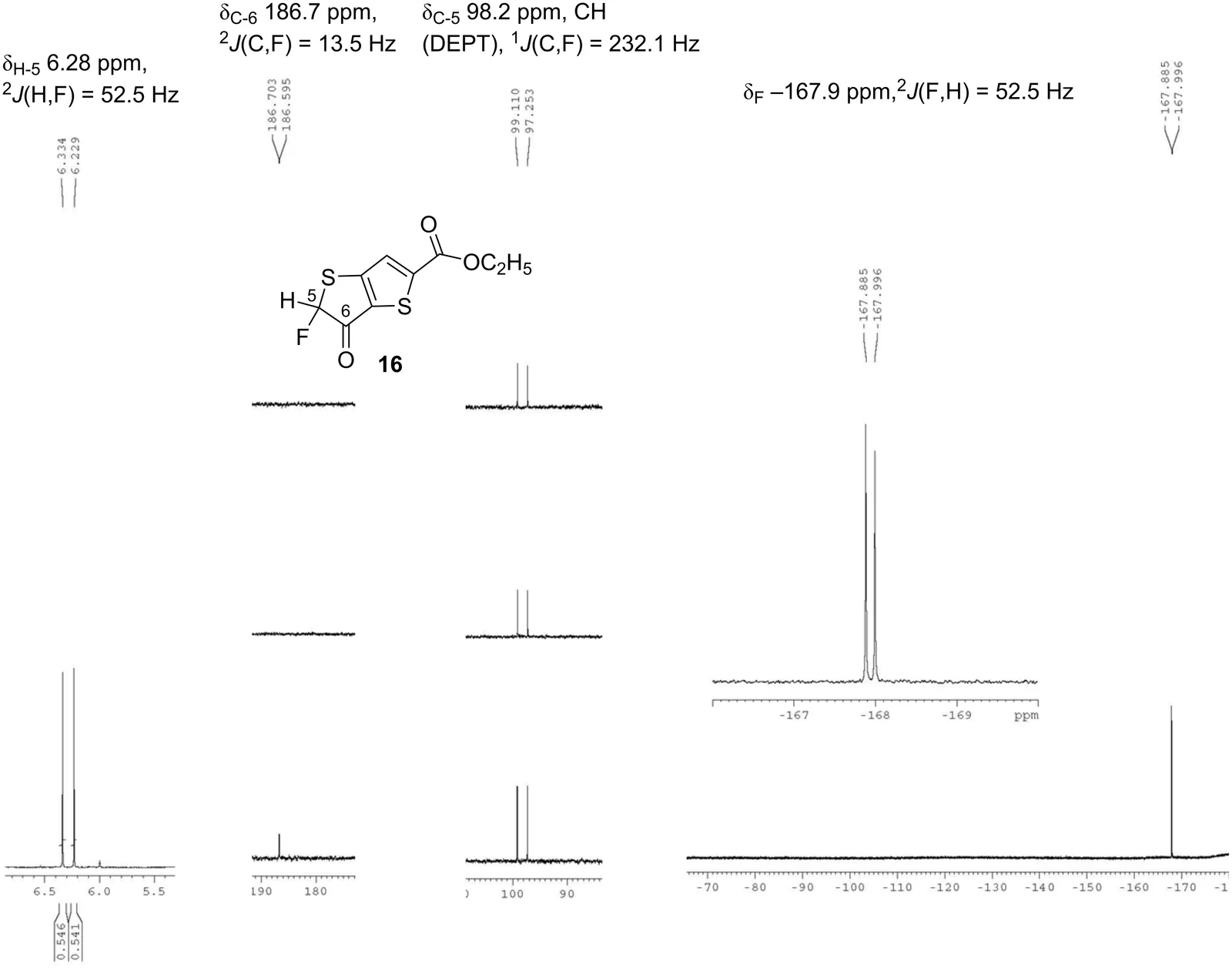
Structure of **16** and the key NMR data that define it. The one-bond C–F coupling of 232.1 Hz on a carbon identified as protonated by DEPT places hydrogen and fluorine on the same sp^3^ center.

Taken together – an oxygen atom more than the aryl fluoride at unchanged hydrogen count, a protonated carbon bonded directly to fluorine, a ring carbonyl two bonds from that fluorine, and a fluorine chemical shift in the saturated region – these observations define the product as ethyl 5-fluoro-6-oxo-5,6-dihydrothieno[3,2-*b*]thiophene-2-carboxylate (**16**), an α-fluorothiophenone in which C5 carries both hydrogen and fluorine while C6 has become a ring ketone (Figure 1). Every resonance is then accounted for: the sp^3^ C5 flanked by sulfur, fluorine, and a carbonyl at δ_C_ 98.2 ppm; the ketone C6 at δ_C_ 186.7 ppm; the enone β-carbon C6a at δ_C_ 157.2 ppm and C3a at δ_C_ 149.8; the surviving aromatic CH at δ_C_ 127.1 (δ_H_ 7.60 ppm, s, uncoupled to fluorine across five bonds) with the ester-bearing C2 at δ_C_ 129.1 ppm; and the ester itself at δ_C_ 160.7, 62.6 and 14.1 ppm, values indistinguishable from those of **8** and **15**.

**Scheme 5:**
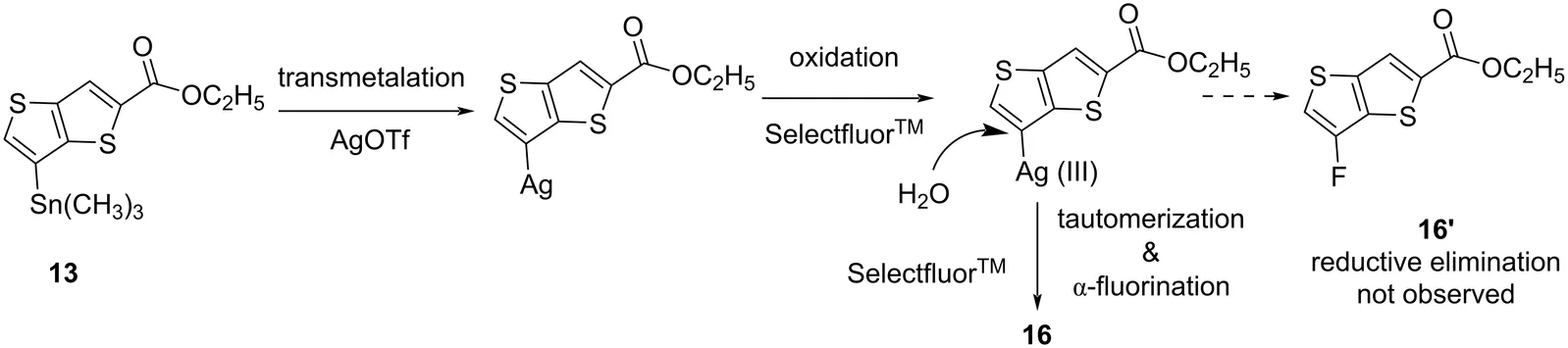
Proposed formation of the α-fluorothiophenone **16**. Interception of the aryl–silver intermediate by oxygen at the carbon that bore tin diverts the sequence away from reductive elimination, and α-fluorination of the resulting thiophenone completes the transformation.

The way **16** arises follows from steps that are individually well precedented (Scheme 5). Transmetalation converts the C6–SnMe_3_ bond into a C6–Ag bond, and oxidation by Selectfluor^TM^ generates a high-valent aryl–silver species, exactly as in the established mechanism for benzenoid substrates [22–23]. On this π-excessive fused thiophene, however, that intermediate is intercepted by oxygen at C6 in preference to C–F reductive elimination. The resulting 6-hydroxyarene is the enol of a thiophen-3(2*H*)-one, a tautomeric equilibrium that lies almost entirely on the keto side for 3-hydroxythiophenes, and the enol of that ketone is precisely the substrate class for which Selectfluor^TM^ is most commonly employed. α-Fluorination at C5 then completes the sequence. Fluorine is thus delivered, but to an sp^3^ carbon two steps removed from the aryl C–F bond that was intended.

This assignment also resolves the behavior of **16** towards a base. Hydrolysis conditions were first calibrated on the fluorine-free analogue, ethyl thieno[3,2-*b*]thiophene-2-carboxylate. TFA/H_2_O mixtures were ineffective from 1:9 to 1:3 and gave only traces of the carboxylic acid even at a 1:1 ratio after 8 h, whereas LiOH effected the conversion cleanly and in 95%. Subjecting **16** to the LiOH protocol produced a complex mixture from which the target acid **17** could not be recovered. The characteristic fluorescence of **16** faded during the reaction, the recovered mass exceeded theory, the ^19^F spectrum showed only a singlet at δ −77.0 ppm attributable to residual triflate, and the measured [M − H]^−^ deviated from any plausible product by more than 400 ppm. Co-spotting against authentic samples of the diacid **15**, obtained by saponification of **14** and of the defluorinated acid, thieno[3,2-*b*]thiophene-2-carboxylic acid, showed the material to be identical to neither, excluding hydrodefluorination and dimerization. An α-fluoroketone bearing an acidic α-C–H is expected to behave in exactly this way: deprotonation at C5 leads to loss of fluoride, and the resulting enolate of a thiophenone is open to further degradation. What appeared to be an unexplained decomposition is a direct consequence of the structure.

The wider lesson concerns the fluorination method rather than this particular substrate. Silver-mediated fluorodestannylation is reliable on benzenoid and moderately electron-rich arenes, and it is on that basis that it is routinely proposed for late-stage fluorination. On a π-excessive fused thiophene bearing an electron-withdrawing ester, the aryl–metal intermediate is sufficiently electrophilic, or sufficiently long-lived, to be intercepted by oxygen before it can be fluorinated. Anyone attempting to fluorinate a thiophene-based semiconductor core by this route should expect C-oxygenation to compete, and the absence of fluorinated thienothiophene acceptors from the literature is likely to have this origin.

### The silver-free control: oxidation without fluorination

Omitting silver shows that it does more than simply accelerate the fluorination. Treatment of **13** with Selectfluor^TM^ alone in acetonitrile – the conditions established for aryltrimethylstannanes [20–21] – gave no fluorinated product whatsoever. The sole isolated product was the symmetrical dimer diethyl [3,3’-bithieno[3,2-*b*]thiophene]-5,5’-dicarboxylate (**14**), formed in 71% yield, with **13** consumed while the co-eluting bromide **7** was recovered untouched (Scheme 4).

A second control excludes the obvious alternative. Stille-type cross-coupling between residual **7** and **13** would give the same dimer, but repeating the reaction on stannane purified to homogeneity by normal-phase HPLC gave **14** in comparable yield. Dimerization therefore proceeds from the stannane alone, without a halide partner and without added palladium, and is most simply understood as single-electron oxidation of this electron-rich arene by Selectfluor^TM^ followed by loss of the trimethylstannyl group and dimerization of the resulting aryl radical.

The two experiments together define the role of silver. Selectfluor^TM^ alone oxidizes the arene; it does not fluorinate it. Silver intercepts the stannane by transmetalation before that oxidation can occur, suppressing dimerization to about 5% and opening the pathway that ultimately delivers **16**. Neither pathway forms an aryl C–F bond.

### The diketopyrrolopyrrole donor block

**Scheme 6:**
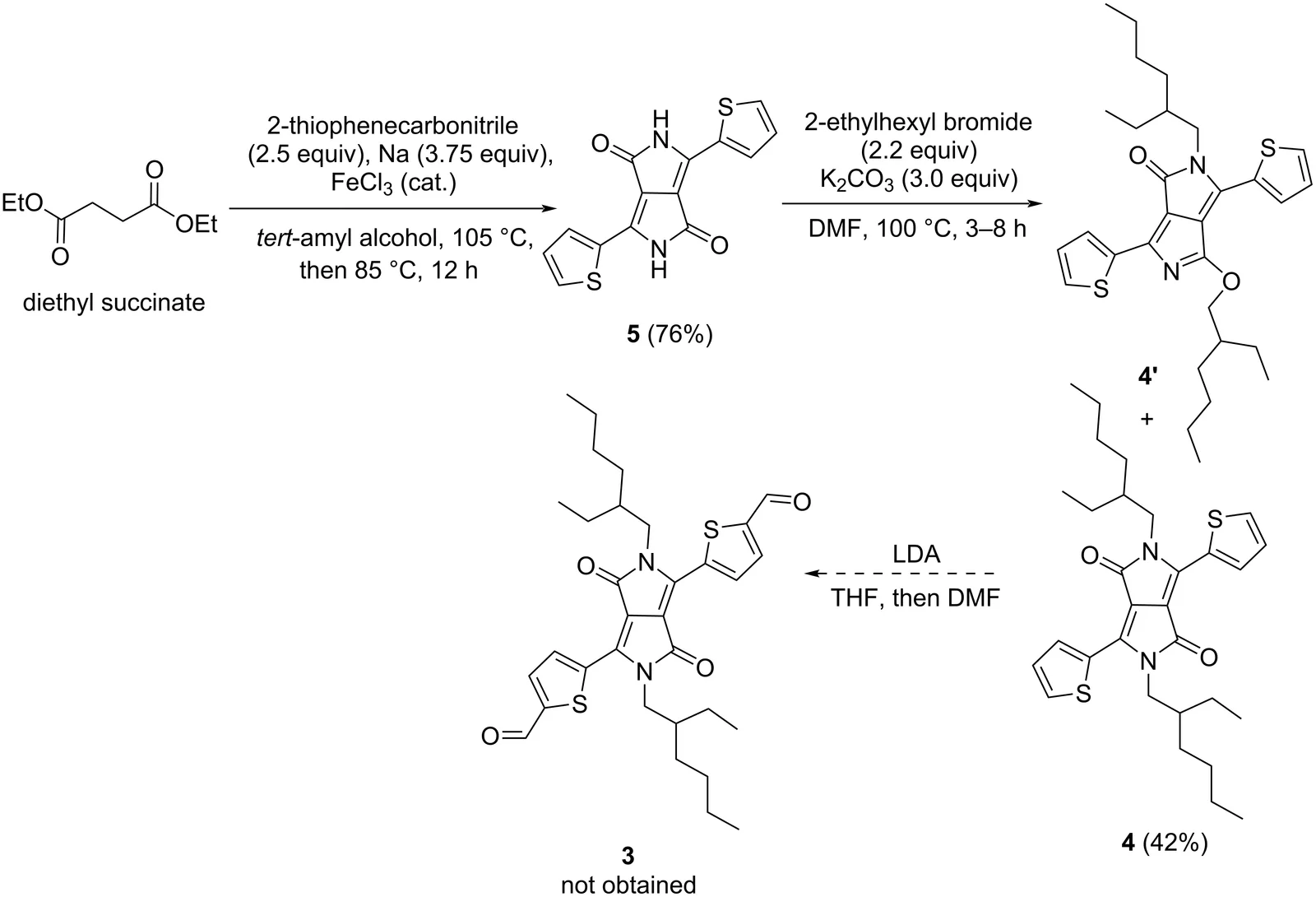
Synthesis of the diketopyrrolopyrrole donor block, showing the competing *O*-alkylation that limits the yield of **4**.

In parallel, the donor fragment was prepared (Scheme 6). Condensation of 2-thiophenecarbonitrile with diethyl succinate under Na/*tert*-amyl alcohol conditions is well precedented but capricious, and reported yields for **5** span 13–40% [39–42]. Three modifications raised it to 76%. First, the reaction is highly moisture-sensitive, and replacing the reflux condenser with a nitrogen balloon at 85 °C rather than heating at reflux improved the yield from 40% to 64%. Second, the acetic acid used to quench the sodium alkoxide must be limited to the calculated equivalence – at ca. 1.5 equiv, a noticeable loss of product was observed. Third, hot methanol followed by water was sufficient to wash away unreacted intermediates, making the acetone/ethyl acetate/hexane washes described previously unnecessary and avoiding the introduction of solvents that are difficult to remove from the poorly soluble product. Residual solvent in **5** is best removed by brief drying at 120 °C (10 min, 28% mass loss) rather than by prolonged high-vacuum treatment, which was ineffective even at 0.03 Torr and 50 °C for 4 h.

*N*,*N*’-Dialkylation of **5** with 2-ethylhexyl bromide/K_2_CO_3_ in DMF gave the soluble derivative **4** in 42% after recrystallization from toluene/hexane. Its yield is limited by a competing *O*-alkylation product, 2-(2-ethylhexyl)-4-((2-ethylhexyl)oxy)-3,6-di(thiophen-2-yl)pyrrolo[3,4-*c*]pyrrol-1(2*H*)-one (**4’**), which appears after 2–4 h and cannot be removed by recrystallization [43–44]. Therefore, the reaction must be stopped at 3–8 h. Product **4’** is readily identified by ^1^H NMR: its six aromatic resonances fall into three inequivalent pairs, showing that the molecular symmetry is broken, and the four-proton N–CH_2_ multiplet at δ 3.9 ppm in **4** is replaced by two-proton signals, at δ 3.9 ppm and at δ 4.5 ppm, the latter consistent with a CH_2_ bound to oxygen.

Formylation of **4** with LDA/DMF, using the conditions that had worked for **6**, did not deliver the bisaldehyde **3**. No aldehyde resonance appeared near δ 10.0 ppm. Instead the aromatic region became complex and three distinct alkyl environments were visible near δ 4.0 ppm, while 80% of **4** was recovered. The pattern suggests competing deprotonation at the acidic *N*-alkyl α-position or attack at the lactam carbonyl rather than at the thiophene α-position. Directed metalation strategies compatible with the DPP lactam, or Vilsmeier-type formylation, are the natural alternatives.

## Conclusion

Fluorination of the thieno[3,2-*b*]thiophene-fused quinoidal acceptor TTC is an obvious target – fluorine is the one substituent shown to deepen the LUMO of this class while simultaneously raising electron mobility – yet it has remained unreported. This study identifies two distinct reasons, one specific and one general.

The specific obstacle is the 1,3-dione. Late-stage functionalization of the assembled tricycle is not viable, because the doubly activated C6 methylene, not the C3–Br bond, is the reactive site under Pd(0)/Me_6_Sn_2_ conditions. A control experiment with 6-bromo-1-indanone, which lacks that methylene and stannylates normally, confirms the diagnosis. Masking both carbonyls, for which a 1,3-dithiane sequence is the logical option, would be required before any organometallic step.

The general obstacle is more consequential. Moving the halogen exchange to the bicyclic stage removes the dione but not the difficulty: the silver-mediated fluorodestannylation of ethyl 6-bromothieno[3,2-*b*]thiophene-2-carboxylate does not give the aryl fluoride. The product, obtained in 27% yield, is the α-fluorothiophenone **16**, whose formation is consistent with an interception of the aryl–silver intermediate by oxygen at the carbon that bore tin, tautomerization of the resulting 6-hydroxyarene to the ketone, and α-fluorination of the enol. Fluorine is delivered, but to an sp^3^ carbon rather than to the ring, and the base sensitivity that frustrated the subsequent ester hydrolysis follows directly from that structure. On a π-excessive fused thiophene, therefore, a method that is dependable on benzenoid arenes is diverted by C-oxygenation – a limitation that, to our knowledge, has not been documented, and one that plausibly explains why fluorinated thienothiophene acceptors have not appeared.

Two controls anchor that conclusion. Without silver, Selectfluor^TM^ merely oxidizes the stannane, which dimerizes to **14** in 71% yield with no fluorine incorporated at all; and repeating that reaction on chromatographically pure stannane shows the dimerization to require no halide partner. Silver therefore does not accelerate a fluorination so much as divert an oxidation.

Access to a fluorinated TTC will require fluorine to be present before the thienothiophene is assembled – most directly from 3-bromo-4-fluorothiophene, which is available from only a few suppliers or by NFSI fluorination – rather than installed on the intact heterocycle. On the donor side, the yield of the DTPP core was raised to 76% by controlling moisture, temperature, and the quench stoichiometry, the *O*-alkylation by-product limiting *N*,*N*’-dialkylation was identified and 1,2-dichloroethane was shown to replace nitrobenzene in the Friedel–Crafts formation of indan-1,3-diones.

## Supporting Information

File 1 Experimental procedures, characterization data, and copies of spectra.

## Data Availability

All data that supports the findings of this study is available in the published article and/or the supporting information of this article.
