# Supplementary material for: Silver-mediated fluorodestannylation of an electron-rich thieno[3,2-b]thiophene diverts to oxygenation: an α-fluorothiophenone instead of an aryl fluoride
Source: Beilstein J Org Chem. 2026 Nov 2;22:1532–43. doi: 10.3762/bjoc.22.124 (PMC13746459; doi:10.3762/bjoc.22.124)
Supplement: Supplementary file 1 [file Beilstein_J_Org_Chem-22-1532-s001.pdf]

BEILSTEIN JOURNAL OF ORGANIC CHEMISTRY

Supporting Information

for

Silver-mediated fluorodestannylation of an electron-rich thieno[3,2-*b*]thiophene diverts to oxygenation: an α-fluorothiophenone instead of an aryl fluoride

Hai-Tung Chuan, Li-Shin Shen, Huai-En Yu, Chia-Chi Chen, Jin-Pei Deng, Rong-Jiun Sheu and Chung-Shan Yu

Beilstein J. Org. Chem. **2026,** *22,* 1532–1543. doi:10.3762/bjoc.22.124

Experimental procedures, characterization data, and copies of spectra

License and Terms: This is a supporting information file under the terms of the Creative Commons Attribution License (https://creativecommons.org/licenses/by/4.0). Please note that the reuse, redistribution and reproduction in particular requires that the author(s) and source are credited and that individual graphics may be subject to special legal provisions.
The license is subject to the *Beilstein Journal of Organic Chemistry* terms and conditions: (https://www.beilstein-journals.org/bjoc/terms)

Contents

Content	Pages
Experimental procedures and characterization data for all compounds	S1
Optimization of the acid chloride formation and Friedel–Crafts annulation (Tables S1, S2)	S9
Literature survey of metal-mediated fluorination of aryl stannanes and boron reagents (Table S3)	S11
Optimization of the ester deprotection (Table S4)	S12
Additional experimental observations	S13
Chromatographic data: TLC *R*f values and HPLC methods (Tables S5, S6)	S15
Spectroscopic basis for the structure of **16** (Table S7)	S16
Copies of NMR and mass spectra, HPLC traces and TLC comparisons (Figures S1–S65)	S17
References	S66

S1 Experimental

S1.1 General remarks

All reactions were carried out under nitrogen in oven-dried glassware. CH_2Cl_2, toluene, Et_3N, MeCN and 1,2-dichloroethane were distilled from CaH_2; MeOH from Mg; DIPA from NaOH. DMF and DMSO were distilled under reduced pressure from $MgSO_4$ and CaH_2, respectively, and stored over 4 Å molecular sieves under nitrogen. THF and 1,4-dioxane were pretreated with $FeSO_4 \cdot 7H_2O$, filtered through Celite, stored over KOH and distilled from sodium benzophenone ketyl. All other reagents were purchased from Sigma-Aldrich, Alfa, TCI or Acros and used as received. Reactions were monitored on Merck 60 F254 silica gel plates visualized at 254 or 366 nm. Normal-phase HPLC was performed on an Agilent 1100 series system (G1310A pump, Rheodyne 7725i injector, 0.5 mL loop; ZORBAX SIL, 9.4 × 250 mm, 5 µm; G1314A variable-wavelength detector at 254 or 366 nm) at 3 mL min^{-1}. Reversed-phase HPLC used a CHEMCOSORB 7-ODS-H column (10 × 250 mm) with $MeCN/H_2O$ 1:1 (isocratic) at 3 mL min^{-1}. NMR spectra were recorded on a Varian Unity Inova 500 spectrometer (^1H 500 MHz, ^{13}C 125 MHz, ^{19}F 470 MHz). HRESIMS was measured on a Bruker Impact HD Q-TOF (charging voltage 2000 V) and HREIMS on a JEOL JMS-T200GC AccuTOF GCx at 70 eV.

3,4-Dibromothiophene-2-carbaldehyde (6)

n-BuLi (2.5 M in hexanes, 18 mL, 45 mmol, 1.1 equiv) was added to THF (16 mL) at −78 °C, followed by DIPA (8.11 mL, 57.9 mmol, 1.4 equiv), and the mixture was stirred for 10 min. 3,4-Dibromothiophene (7.5 mL, 41.3 mmol, 1.0 equiv) in THF (2.5 mL) was added, the cooling bath was replaced by an ice bath, and stirring was continued for 1 h. DMF (4 mL, 53.7 mmol, 1.3 equiv) was

added, the ice bath removed, and the mixture stirred for 5 h. After quenching with NH_4Cl to pH 5, aqueous work-up and flash chromatography (EtOAc/hexanes 1:39 → 1:19) gave **6** as a bright yellow powder (4.35 g, 16.2 mmol, 40%). Titration of the *n*-BuLi and adjustment of the stoichiometry raised the yield to 74% (Section S5).

^{1}H NMR (500 MHz, $CDCl_3$): δ 7.73 (d, *J* = 1.5 Hz, 1H), 9.93 (d, *J* = 1.5 Hz, 1H, CHO). ^{13}C NMR (125 MHz, $CDCl_3$): δ 116.1, 123.5, 131.7, 137.7, 182.9 (CHO). HREIMS: *m/z* calcd for $C_5H_2Br_2OS$ $[M]^+$ 269.8173, found 269.8166 (Δ = −2.5 ppm).

Ethyl 6-bromothieno[3,2-*b*]thiophene-2-carboxylate (7)

To oven-dried K_2CO_3 (1.78 g, 14.4 mmol, 2.0 equiv) in anhydrous DMF (15 mL) were added **6** (1.99 g, 7.41 mmol) and ethyl thioglycolate (1.22 mL, 11.1 mmol, 1.5 equiv). After 1.5 h at room temperature, aqueous work-up and flash chromatography (EtOAc/hexanes 1:29 → 1:19) gave **7** as a white powder (1.90 g, 6.60 mmol, 89%).

^{1}H NMR (500 MHz, $CDCl_3$): δ 1.38 (t, *J* = 7.0 Hz, 3H), 4.37 (q, *J* = 7.0 Hz, 2H), 7.45 (s, 1H), 7.99 (s, 1H). ^{13}C NMR (125 MHz, $CDCl_3$): δ 14.3, 61.6, 102.8 (C–Br), 126.1, 128.2, 136.1, 137.6, 144.7, 162.2 (CO_2Et). HRESIMS: *m/z* calcd for $C_9H_8BrO_2S_2$ $[M + H]^+$ 290.9149, found 290.9144 (Δ = −1.7 ppm).

6-Bromothieno[3,2-*b*]thiophene-2-carboxylic acid (8)

Compound 7 (493 mg, 1.72 mmol) was treated with LiOH (110 mg, 4.30 mmol, 2.5 equiv) in THF/H_2O (1:1, 6 mL) at 80 °C for 2 h. Acidification to pH 4 with HCl and extraction with EtOAc gave **8** as a white powder (438 mg, 1.67 mmol, 98%).

^{1}H NMR (500 MHz, DMSO-d_6): δ 8.07 (s, 1H), 8.20 (s, 1H), 13.49 (s, 1H, CO_2H). ^{13}C NMR (125 MHz, DMSO-d_6): δ 101.5 (C–Br), 127.4, 130.5, 136.5, 138.2, 143.3, 163.0 (CO_2H). HRESIMS: *m/z* calcd for $C_7H_2BrO_2S_2$ $[M - H]^-$ 260.8685, found 260.8677 (Δ = −3.1 ppm).

3-Bromo-5*H*-cyclopenta[*b*]thieno[2,3-*d*]thiophene-5,7(6*H*)-dione (10)

Caution**:** copious HCl is evolved; acid-resistant tubing must be used.

Acid chloride formation. **8** (100 mg, 0.38 mmol) was heated at reflux in $SOCl_2$ (3 mL) at 105 °C. After 30 min the suspension became a clear wine-red solution. Concentration and azeotropic removal of residual $SOCl_2$ with freshly distilled toluene (3 mL) gave crude **9** as an orange solid, used without purification.

Friedel–Crafts annulation. $AlCl_3$ (327 mg, 2.46 mmol, 5.2 equiv) in 1,2-dichloroethane (2 mL) was treated at 0 °C with malonyl dichloride (0.16 mL, 1.6 mmol, 3.3 equiv) and stirred for 15 min. **9** (140 mg, 0.48 mmol) in 1,2-dichloroethane (2 mL) was added slowly and the mixture heated at 85 °C for 4 h, then poured into 10% aqueous oxalic acid (30 mL) and stirred vigorously for 30 min. Neutralization with $NaHCO_3$, extraction with CH_2Cl_2, drying over $MgSO_4$ and flash chromatography (CH_2Cl_2/hexanes 3:7 → 1:1) gave **10** as a bright yellow powder (14.2 mg, 0.05 mmol, 13% based on **8**). An analytical sample was further purified by normal-phase HPLC (CH_2Cl_2, 3 mL min^{-1}, 366 nm, t_R = 10.2 min).

^{1}H NMR (500 MHz, $CDCl_3$): δ 3.49 (s, 2H, CH_2), 7.68 (s, 1H). ^{13}C NMR (125 MHz, $CDCl_3$): δ 48.3 ($COCH_2CO$), 103.4 (C–Br), 129.7, 131.8, 149.8, 151.9, 158.1, 188.5, 188.5 (2 × C=O). HREIMS: *m/z* calcd for $C_9H_3BrO_2S_2$ $[M]^+$ 285.8758, found 285.8744 (Δ = −4.9 ppm).

1*H*-Indene-1,3(2*H*)-dione (indan-1,3-dione)

$AlCl_3$ (1.53 g, 11.5 mmol, 2.3 equiv) in 1,2-dichloroethane (5 mL) was treated at 0 °C with malonyl dichloride (1.96 mL, 20 mmol, 4.0 equiv) and stirred for 15 min. Benzoyl chloride (0.58 mL, 5.0 mmol) in 1,2-dichloroethane (2 mL) was added slowly and the mixture heated at 80 °C for 12 h. Work-up with 5% aqueous oxalic acid, neutralization with saturated $NaHCO_3$, extraction with CH_2Cl_2 and flash chromatography (EtOAc/hexanes 1:19 → 1:9) gave indan-1,3-dione as a yellow-green powder (140 mg, 0.95 mmol, 41%; up to 45% on optimization, Table S2).

^{1}H NMR (500 MHz, $CDCl_3$): δ 3.23 (s, 2H, CH_2), 7.83 (m, 2H), 7.97 (m, 2H).

6-(Trimethylstannyl)-2,3-dihydro-1*H*-inden-1-one (18) – control experiment

This compound was prepared only to establish that a substrate lacking a doubly activated methylene stannylates normally, and is identified by its ^{1}H NMR spectrum rather than claimed as a fully characterized new compound.

6-Bromo-1-indanone (180 mg, 0.85 mmol) in degassed 1,4-dioxane (3 mL) was treated with Me_6Sn_2 (0.25 mL, 1.2 mmol, 1.4 equiv) and $Pd(PPh_3)_2Cl_2$ (21 mg, 0.04 equiv) and heated at 80 °C until the solution turned black. Concentration and flash chromatography (EtOAc/hexanes 1:19 → 1:9) gave **18** as a yellow oil (75 mg, 0.25 mmol, 30%).

^{1}H NMR (500 MHz, $CDCl_3$): δ 0.29 (t, *J* = 28.0 Hz, 9H, $SnMe_3$), 2.65 (td, *J* = 5.5, 3.0 Hz, 2H), 3.09 (td, *J* = 6.0, 3.0 Hz, 2H), 7.44 (d, *J* = 7.0 Hz, 1H, H4), 7.68 (td, *J* = 19.5, 7.5 Hz, 1H, H5), 7.89 (t, *J* = 21.5 Hz, 1H, H7). The triplet appearance of the H5 and H7 resonances arises from unresolved ^{117}Sn/^{119}Sn satellites.

Ethyl 6-(trimethylstannyl)thieno[3,2-*b*]thiophene-2-carboxylate (13)

Compound 7 (270 mg, 0.95 mmol) in degassed toluene (3 mL) was treated with Me_6Sn_2 (0.45 mL, 2.27 mmol, 2.4 equiv) and $Pd(PPh_3)_4$ (45 mg, 0.04 equiv) and heated at 80 °C for 4 h, the reaction being stopped as soon as the solution darkened. Flash chromatography on silica pretreated with 3% Et_3N (EtOAc/hexanes 1:49 → 1:29) gave **13** as a colorless oil (140 mg, 0.37 mmol, 40% by mass; 35% corrected for residual **7** and the protodestannylated arene by ^{1}H NMR). An analytical sample was obtained by normal-phase HPLC (EtOAc/hexanes 1:19, 3 mL min^{-1}, 254 nm, t_R = 6.2 min).

^{1}H NMR (500 MHz, $CDCl_3$): δ 0.41 (t, *J* = 28.0 Hz, 9H, $SnMe_3$), 1.38 (t, *J* = 7.0 Hz, 3H), 4.36 (q, *J* = 7.0 Hz, 2H), 7.49 (t, *J* = 13.5 Hz, 1H), 8.02 (t, *J* = 2.5 Hz, 1H). ^{13}C NMR (125 MHz, $CDCl_3$): δ −9.2 ($SnMe_3$), 14.3, 61.3, 125.6, 131.7, 134.6, 137.4, 139.0, 151.5, 162.7 (CO_2Et). HREIMS: *m/z* calcd for $C_{12}H_{16}O_2S_2{}^{120}Sn$ $[M]^+$ 375.9614, found 375.9618 (Δ = 1.1 ppm); calcd for $[M - CH_3]^+$ 360.9379, found 360.9383 (Δ = 1.1 ppm).

Ethyl 6-(tributylstannyl)thieno[3,2-*b*]thiophene-2-carboxylate (13′)

Prepared as for **13** from **7** (2.80 g, 10 mmol), Bu_6Sn_2 (18.2 mL, 36 mmol, 3.6 equiv) and $Pd(PPh_3)_4$ (580 mg, 0.05 equiv) in degassed toluene (15 mL) at 80 °C for 16 h. Flash chromatography (EtOAc/hexanes 1:39 → 1:19) gave **13′** as a colorless oil (2.5 g, 5.0 mmol, 50% by mass); an analytical sample was obtained by normal-phase HPLC (EtOAc/hexanes 1:19, 254 nm, t_R = 5.6 min), corresponding to 27% of pure material.

^{1}H NMR (500 MHz, $CDCl_3$): δ 0.87 (t, J = 7.0 Hz, 9H), 1.17 (t, J = 7.0 Hz, 6H), 1.32 (td, J = 7.5, 7.0 Hz, 6H), 1.38 (t, J = 7.0 Hz, 3H), 1.55 (m, 6H), 4.36 (q, J = 7.0 Hz, 2H), 7.46 (t, J = 10.5 Hz, 1H), 8.01 (t, J = 2.0 Hz, 1H). ^{13}C NMR (125 MHz, $CDCl_3$): δ 10.1, 13.6, 14.3, 27.0, 29.0, 61.2, 125.5, 131.5, 134.4, 137.3, 138.9, 152.1, 162.8 (CO_2Et). HREIMS: m/z calcd for $C_{21}H_{34}O_2S_2{}^{120}Sn$ $[M]^+$ 502.1022, found 502.1015 (Δ = −1.4 ppm); calcd for $[M - C_4H_9]^+$ 445.0318, found 445.0328 (Δ = 2.2 ppm).

Ethyl 5-fluoro-6-oxo-5,6-dihydrothieno[3,2-*b*]thiophene-2-carboxylate (16)

Compound 13 (30 mg, 0.08 mmol) in freshly distilled acetone (1 mL) was treated with AgOTf (43 mg, 0.16 mmol, 2.0 equiv), prepared in situ from Ag_2O and TfOH, and after 1 min with Selectfluor™ (32.4 mg, 0.09 mmol, 1.15 equiv). The reaction was complete within 5–15 min. Concentration and flash chromatography (EtOAc/hexanes 1:29 → 1:9) returned the protodestannylated arene, ethyl thieno[3,2-*b*]thiophene-2-carboxylate (11 mg, 65%) and gave **16** as a yellow powder (5.4 mg, 0.023 mmol, 32% by mass; 27% corrected for co-eluting **14** by ^{1}H NMR).

Spectra were recorded on material enriched in **16** but still containing **14**; signals assignable to **14** are listed separately.

^{1}H NMR (500 MHz, $CDCl_3$): δ 1.38 (t, J = 7.0 Hz, 3H, CH_3), 4.39 (q, J = 7.0 Hz, 2H, OCH_2), 6.28 (d, ^2J(H,F) = 52.5 Hz, 1H, H5), 7.60 (s, 1H, H3). ^{19}F NMR (470 MHz, $CDCl_3$): δ −167.9 (d, ^2J(F,H) = 52.2 Hz). ^{13}C NMR (125 MHz, $CDCl_3$): δ 14.1 (CH_3), 62.6 (OCH_2), 98.2 (CH, d, ^1J(C,F) = 232.1 Hz, C5, S–CHF–C=O), 127.1 (CH, C3), 129.1 (C, C2), 149.8 (C, C3a), 157.2 (C, C6a), 160.7 (C, CO_2Et), 186.7 (C, d, ^2J(C,F) = 13.5 Hz, C6, ring C=O). Signals of **15**: δ 14.3, 61.6, 125.9, 127.2, 127.7, 135.6, 138.7, 141.6, 162.4. HREIMS: m/z calcd for $C_9H_7FO_3S_2$ $[M]^+$ 245.9821, found 245.9822 (Δ = 0.4 ppm); no ion corresponding to the aryl fluoride $C_9H_7FO_2S_2$ (m/z 229.9871) was detected.

Using **13′** (2.25 g, 4.5 mmol), AgOTf (1.7 g, 6.6 mmol, 1.5 equiv), $NaHCO_3$ (400 mg, 4.76 mmol, 1.1 equiv) and F-TEDA-PF_6 (5.11 mmol, 1.15 equiv) in acetone (10 mL) at 0 °C – the conditions of Table 2, entry 6 – gave, after chromatography, the protodestannylated arene (800 mg, 3.7 mmol, 82%) and **16** (160 mg, 0.7 mmol, 15% isolated; 16% by ^{1}H NMR of the crude mixture), spectroscopically identical to the above.

Diethyl [3,3′-bithieno[3,2-*b*]thiophene]-5,5′-dicarboxylate (14)

A mixture of **7** and **13** (250 mg, 0.67 mmol total) in freshly distilled MeCN (3 mL) was treated with Selectfluor™ (500 mg, 1.41 mmol, 1.3 equiv) and heated at 50 °C for 2 h. Concentration and flash chromatography (EtOAc/hexanes 1:39 → 1:19) returned unreacted **7** (53 mg, 0.18 mmol) and gave **14** as a yellow powder (99.5 mg, 0.24 mmol, 71%). The same product was obtained from HPLC-purified **13**.

^{1}H NMR (500 MHz, $CDCl_3$): δ 1.41 (t, *J* = 7.0 Hz, 6H), 4.40 (q, *J* = 7.0 Hz, 4H), 7.94 (s, 2H), 8.05 (s, 2H). ^{13}C NMR (125 MHz, $CDCl_3$): δ 14.3, 61.6, 125.9, 127.2, 127.7, 135.6, 138.7, 141.6, 162.4 (CO_2Et). HREIMS: *m/z* calcd for $C_{18}H_{14}O_4S_4$ $[M]^+$ 421.9775, found 421.9778 (Δ = 0.7 ppm). Melting point not determined; the sample was largely consumed in the subsequent deprotection experiments.

[3,3′-Bithieno[3,2-*b*]thiophene]-5,5′-dicarboxylic acid (15) – reference sample

This compound was prepared solely as an authentic sample for the chromatographic comparison described in Section S5 and is not claimed as a fully characterized new compound.

Compound 14 (11.3 mg, 0.03 mmol) was treated with LiOH (30 mg, 3.0 equiv) in THF/H_2O (1:1, 1 mL) at 80 °C for 2 h. Acidification to pH 4 with HCl and extraction with EtOAc gave crude **15** as a yellow powder (7.2 mg). Recrystallization from hot MeOH afforded material of sufficient purity for TLC comparison (4.0 mg), mp 202–203 °C.

^{1}H NMR (400 MHz, DMSO-d_6): δ 8.20 (s, 2H), 8.27 (s, 2H), consistent with the C_2-symmetric diacid.

Ethyl thieno[3,2-*b*]thiophene-2-carboxylate

This is a known, commercially available compound (CAS 201004-08-2). It is formed in this work as the protodestannylation by-product of the silver-mediated fluorination of **13** (Table 2) and was used as the fluorine-free substrate for calibrating the ester hydrolysis conditions (Table S4).

Thieno[3,2-*b*]thiophene-2-carboxylic acid

Ethyl thieno[3,2-*b*]thiophene-2-carboxylate (100 mg, 0.47 mmol) was treated with LiOH (35 mg, 3.0 equiv) in THF/H_2O 1:1 (1.6 mL) at 80 °C for 2 h. Acidification to pH 4 and extraction with EtOAc gave the acid as a white powder (83 mg, 0.44 mmol, 95%). Residual solvent was removed by three azeotropic co-evaporations with $CHCl_3$/MeOH.

^{1}H NMR (500 MHz, $CD_3OD/CDCl_3$ 2:1): δ 7.29 (dd, *J* = 5.0, 2.5 Hz, 1H), 7.66 (m, 1H), 7.95 (d, *J* = 2.5 Hz, 1H).

3,6-Di(thiophen-2-yl)-2,5-dihydropyrrolo[3,4-*c*]pyrrole-1,4-dione (5)

Sodium (3.45 g, 150 mmol, 3.75 equiv) and $FeCl_3$ (50 mg) in *tert*-amyl alcohol (50 mL) were stirred vigorously at 105 °C for 2 h. The bath was cooled to 85 °C, 2-thiophenecarbonitrile (9.3 mL, 100 mmol, 2.5 equiv) was added, and a solution of diethyl succinate (6.7 mL, 40 mmol, 1.0 equiv) in *tert*-amyl alcohol (5 mL) was introduced dropwise. The reflux condenser was replaced by a nitrogen balloon and stirring continued for 12 h at 85 °C. MeOH (10 mL) was added, followed by exactly the calculated equivalence of acetic acid (excess causes product loss), until pH < 7. The solid was collected and washed successively with hot MeOH and H_2O until the washings were no longer yellow-green, giving **5** as a wine-red solid (11.48 g, 38.2 mmol, 76%).

Residual solvent is best removed by drying at 120 °C for 10 min (28% mass loss); prolonged high-vacuum treatment (0.03 Torr, 50 °C, 4 h) is ineffective.

^{1}H NMR (500 MHz, DMF-d_7): δ 7.35 (dd, *J* = 5.0, 1.0 Hz, 2H), 8.01 (t, *J* = 5.0 Hz, 2H), 8.41 (dd, *J* = 4.0, 1.0 Hz, 2H), 11.16 (s, 2H, NH).

2,5-Bis(2-ethylhexyl)-3,6-di(thiophen-2-yl)-2,5-dihydropyrrolo[3,4-*c*]pyrrole-1,4-dione (4)

Oven-dried K_2CO_3 (1.46 g, 10 mmol, 3.0 equiv) in anhydrous DMF (15 mL) was treated with **5** (1.00 g, 3.33 mmol, dried at 120 °C for 10 min) and heated at 100 °C for 30 min. 2-Ethylhexyl bromide (1.4 mL, 7.4 mmol, 2.2 equiv) in DMF (1.5 mL) was added and heating continued for 3–8 h; the reaction must be stopped before **4′** accumulates. H_2O (15 mL) was added, the precipitate dissolved in $CHCl_3$ (300 mL),

washed with H_2O and concentrated. Flash chromatography (EtOAc/hexanes 1:24 → 1:14) gave crude **4** (911 mg, 52%). Dissolution in hot toluene (25 mL) followed by addition of a small volume of ice-cold hexane and a second chromatography gave pure **4** as a bright red solid (42%).

^{1}H NMR (500 MHz, $CDCl_3$): δ 0.81–0.87 (m, 12H), 1.21–1.37 (m, 16H), 1.84 (t, J = 6.0 Hz, 2H), 4.00 (m, 4H, NCH_2), 7.25 (d, J = 4.5 Hz, 2H), 7.60 (d, J = 5.0 Hz, 2H), 8.86 (d, J = 4.5 Hz, 2H). ^{13}C NMR (125 MHz, $CDCl_3$): δ 10.4, 14.0, 23.0, 23.5, 28.3, 30.2, 39.0, 45.8, 107.9, 128.4, 129.8, 130.5, 135.2, 140.4, 161.7 (C=O). HRESIMS: *m/z* calcd for $C_{30}H_{41}N_2O_2S_2$ $[M + H]^+$ 525.2604, found 525.2608 (Δ = −0.7 ppm).

2-(2-Ethylhexyl)-4-((2-ethylhexyl)oxy)-3,6-di(thiophen-2-yl)pyrrolo[3,4-*c*]pyrrol-1(2*H*)-one (4′)

Isolated as the principal by-product of the alkylation (R_f = 0.44, EtOAc/hexanes 1:9).

^{1}H NMR (500 MHz, $CDCl_3$): δ 0.83–0.98 (m, 12H), 1.20–1.34 (m, 16H), 1.76 (m, 2H), 3.93 (m, 2H, NCH_2), 4.51 (m, 2H, OCH_2), 7.19 (t, J = 4.0 Hz, 1H), 7.22 (t, J = 4.5 Hz, 1H), 7.50 (d, J = 5.0 Hz, 1H), 7.68 (d, J = 5.0 Hz, 1H), 8.19 (d, J = 4.0 Hz, 1H), 8.47 (d, J = 3.5 Hz, 1H). The six inequivalent aromatic resonances and the two distinct two-proton CH_2 signals establish the loss of molecular symmetry and the presence of an O-bound alkyl chain.

Reagents prepared in house

AgOTf. Following a published procedure [S1], Ag_2O (1.00 g, 4.3 mmol) in THF (25 mL) protected from light was treated with TfOH (0.8 mL, 9.06 mmol, 2.1 equiv) for 1 h. Removal of solvent gave AgOTf as an off-white powder, used directly.

F-TEDA-PF_6. Selectfluor™ (1.06 g, 3.0 mmol) in H_2O (9 mL) was treated with NH_4PF_6 (2.93 g, 18 mmol, 6.0 equiv) for 1 h. The precipitate was washed with H_2O (5 × 5 mL) and EtOAc (2 × 5 mL) and dried (1.01 g, 2.3 mmol, 75%). ^{1}H NMR (500 MHz, acetone-d_6): δ 4.98 (s, 6H), 5.40 (d, J = 6.0 Hz, 6H), 5.95 (d, J = 1.0 Hz, 2H). ^{19}F NMR (470 MHz, acetone-d_6): δ −67.1 (dd, J = 706.9, 8.0 Hz, PF_6^-), 53.2 (s, N–F).

Unsuccessful transformations

*Attempted stannylation of **10**.* Compound **10** was treated with Me_6Sn_2 (2.4 equiv) and either $Pd(PPh_3)_2Cl_2$ (1,4-dioxane, 80 °C) or $Pd(PPh_3)_4$ (THF, reflux). In both cases starting material was consumed within 1 h. ^{1}H NMR of the crude product showed loss of the C6 methylene singlet at δ 3.49 (2H), appearance of a one-proton multiplet at δ 4.2, and a downfield shift of the aromatic singlet from δ 7.68 to δ 8.1; the yellow fluorescence of **10** was lost and the product was a brown oil with an organotin odor. No arylstannane **11** was detected.

*Attempted hydrolysis of **16**.* Compound **16** (containing **14**) was treated with LiOH (3.0 equiv) in THF/H_2O 1:1 at 80 °C for 1 h and acidified to pH 4. The recovered material exceeded the theoretical mass, showed a single ^{19}F singlet at δ −77.0 (residual triflate) with no aromatic C–F doublet, and gave a $[M - H]^-$ ion deviating from the value calculated for **17** by >400 ppm. TLC co-spotting against **15** and thieno[3,2-*b*]thiophene-2-carboxylic acid excluded dimerization and hydrodefluorination. **17** was not obtained.

*Attempted formylation of **4**.* Compound **4** was treated with LDA (from *n*-BuLi/DIPA) followed by DMF under the conditions used for **6**. No resonance was observed near δ 10.0; the aromatic region became complex, three alkyl environments appeared near δ 4.0, and 80% of **4** was recovered. **3** was not obtained.

S2 Optimization of the acid chloride formation and Friedel–Crafts annulation

Acid **8** is insoluble in chlorinated solvents, and activation to the acid chloride **9** therefore requires either catalytic DMF as a solubilizing co-catalyst or neat $SOCl_2$ as the reaction medium. Two variables were examined: the DMF loading during activation, and the stoichiometry and solvent of the subsequent $AlCl_3$-promoted annulation. The crude acid chloride **9** was never purified, so the yields in Table S1 are for the two operations combined and are based on **8**.

Table S1: Formation of acid chloride **9** and Friedel–Crafts annulation to **10**.

Entry	Solvent (activation)	$SOCl_2$ (equiv)	DMF (equiv)	Time (h)[a]	Annulation conditions[b]	Yield of **10** (%)
1	$CHCl_3$[c]	4	0.3	3	A	10–17[d]
2	$CHCl_3$[c]	4	1.2	1.5	A	0–5[d]
3	$CHCl_3$	4	1.2	1.5	B	8
4	CH_2Cl_2	4	0.4	4	B	12
5	DCE	4	0.4	0.5	B	13
6	$SOCl_2$ (neat)	100	—	1	B	13

[a]Time to complete consumption of **8**. [b]Conditions A: malonyl dichloride (4.5 equiv), $AlCl_3$ (6.0 equiv), CH_2Cl_2, reflux, 12–16 h. Conditions B: malonyl dichloride (3.3 equiv), $AlCl_3$ (2.3 equiv), 1,2-dichloroethane (DCE), reflux, 12–16 h. [c]$CHCl_3$ containing 1% ethanol as stabiliser gave <5% of **10** together with an ethyl ester by-product; ethanol-free $CHCl_3$ must be used. [d]Repeated runs under these conditions gave a maximum of 17%, most commonly 10–12%, and occasionally no product.

Practical notes. Above one equivalent of DMF the salts formed during activation cannot be removed under reduced pressure, and the concentrated residue fumes on exposure to air; 0.3–0.4 equiv leaves a tractable solid. Using neat $SOCl_2$ avoids the problem entirely: the suspension of **8** dissolves within 30 min at 105 °C to give a clear wine-red solution, and excess $SOCl_2$ is removed by azeotropic co-evaporation with freshly distilled toluene until the sulfurous odor is no longer detectable. Because **9** is highly moisture-sensitive, TLC monitoring of the annulation was abandoned in favor of a fixed 12–16 h reaction time; CH_2Cl_2 evaporates so rapidly from the capillary that repeated sampling causes a brown precipitate to deposit which does not redissolve.

Because the yield of **10** never exceeded 17%, benzoyl chloride was used as a model substrate to determine whether the limitation lays in the conditions or in the substrate. The two decisive variables proved to be which acyl chloride is pre-activated with $AlCl_3$ and the reaction temperature (Table S2).

Table S2: Model annulation of benzoyl chloride to 1*H*-indene-1,3(2*H*)-dione.

**Entry**	**$AlCl_3$ (equiv)**	**Malonyl dichloride (equiv)**	**Pre-activated with $AlCl_3$[a]**	**Solvent[b]**	**Yield (%)**
1	3.0	1.3	benzoyl chloride	CH_2Cl_2	7
2	3.0	4.0	benzoyl chloride	CH_2Cl_2	10
3	2.3	4.0	malonyl dichloride	CH_2Cl_2	24
4	2.3	4.0	malonyl dichloride	DCE	45

[a]The stated acyl chloride was stirred with $AlCl_3$ at 0 °C for 15 min before the second acyl chloride was added. [b]Reflux temperature ca. 50 °C in CH_2Cl_2 and ca. 90 °C in 1,2-dichloroethane (DCE).

Preactivating malonyl dichloride rather than the aroyl chloride raised the yield from 10% to 24% (entries 2 and 3), and raising the temperature by moving from CH_2Cl_2 to DCE raised it further to 45% (entry 4). Published preparations of indan-1,3-diones use nitrobenzene at 80–140 °C and report 33–50% [S2,S3]; DCE (bp 83.5 °C) reaches the same temperature window and is not a controlled toxic substance, so it is a directly substitutable and less hazardous alternative. Applying the optimized conditions of entry 4 to **9** nonetheless gave **10** in only 13%, establishing that the low yield is intrinsic to the electron-rich thienothiophene substrate.

S3 Literature survey of metal-mediated fluorination of arylstannanes and boron reagents

Because direct electrophilic fluorination of thiophenes is either poorly regioselective or low-yielding, fluorine was installed by halogen-to-fluorine exchange through an arylstannane. Table S3 summarizes the reported yields [S4–S11] that guided the choice of conditions. Uncatalyzed fluorination with Selectfluor™ is effective only for aryltrimethylstannanes; aryltributylstannanes require a silver or copper promoter.

Table S3: Reported yields for fluorination of arylstannanes and boron reagents.

Ar–X	Fluorinating agent	Promoter	Yield (%)
$SnMe_3$	Selectfluor™	none	21–40 [S4,S5]
$SnBu_3$	Selectfluor™	AgOTf	70 [S6]
$SnBu_3$	Selectfluor™	NaOTf + 10 mol % AgOTf	68 [S7]
$SnBu_3$	F-TEDA-PF_6[a]	AgOTf	83 [S6]
$SnBu_3$	F-TEDA-PF_6	NaOTf + 5 mol % Ag_2O + $NaHCO_3$	75–90 [S7]
$SnBu_3$	NFTPT[b]	$(t\text{-BuCN})_2CuOTf$	74 [S8]
$SnBu_3$	NFTPT	$(MeCN)_4CuBF_4$	60 [S8]
$SnBu_3$	NFTPT	$Cu(OTf)_2$	23 [S8]
$SnBu_3$	NFTPT	$(t\text{-BuCN})_2CuOTf$	30–70 [S8]
Bpin	NFTPT	$(t\text{-BuCN})_2CuOTf$	9–40 [S8]
$SnBu_3$	Selectfluor™	$(t\text{-BuCN})_2CuOTf$	11–30 [S8]
$SnBu_3$	NFSI	$(t\text{-BuCN})_2CuOTf$	9–23 [S8]
$SnBu_3$	KF	$Cu(OTf)_2$ + 18-crown-6 + pyridine	55[c] [S9]
$SnBu_3$	TBAT[d]	$Cu(OTf)_2$	50–70 [S10]
Bpin	AgF (KF)	$Cu(OTf)_2$	56 [S11]

[a]F-TEDA-PF_6 = 1-(chloromethyl)-4-fluoro-1,4-diazoniabicyclo[2.2.2]octane bis(hexafluorophosphate), the PF_6 salt of Selectfluor™. [b]NFTPT = 1-fluoro-2,4,6-trimethylpyridinium triflate. [c]30 min reaction time. [d]TBAT = tetrabutylammonium difluorotriphenylsilicate.

S4 Optimization of the ester deprotection

Conditions for removing the ethyl ester were calibrated on the fluorine-free analogue, ethyl thieno[3,2-*b*]thiophene-2-carboxylate, before being applied to **16**, so that decomposition of the fluorinated substrate could be distinguished from a generally unsuitable protocol.

Table S4: Deprotection of ethyl thieno[3,2-*b*]thiophene-2-carboxylate.

Entry	Reagent	Solvent ratio	Temperature	Time (h)	Outcome
1	TFA/H_2O	1:9	reflux	8	no consumption of the ester
2	TFA/H_2O	1:3	reflux	8	no consumption of the ester
3	TFA/H_2O	1:1	reflux	8	slow consumption; trace acid only
4	LiOH (3.0 equiv)	THF/H_2O 1:1	80 °C	2	acid, 95%

Applying entry 4 to **16** gave a complex mixture from which **17** could not be recovered (main text, and Figures S55–S61).

S5 Additional experimental observations

Titration of *n*-BuLi (compound 6). Deprotonation of 3,4-dibromothiophene at −78 °C returned unchanged starting material; the reaction proceeds only at 0 °C. With a bottle of *n*-BuLi that had been stored for four years and was cloudy with white precipitate, the formylation gave **6** in 39.7%. Performing a small-scale test reaction, calculating the effective titre from the recovered starting material and correcting the stoichiometry accordingly raised the yield to 74% using the same reagent, close to the 80–90% reported in the literature [S12,S13]. Cloudy *n*-BuLi is therefore usable provided the titre is determined. Exceeding 1.1 equiv of LDA generates two more polar by-products (EtOAc/hexanes 1:9, R_f 0.40 and 0.34; product R_f 0.54) and lowers the yield. Because 3,4-dibromothiophene is volatile, a separate aliquot must be reserved as the TLC reference.

Ionization of compound 6. Under ESI(+) the $[M + H]^+$ signal of **6** reached only about 10% of the background at *m/z* 282.27, because the low-polarity $CHCl_3$ used to dissolve the sample ionizes poorly. Switching to EI gave strong, well-resolved isotope patterns for both $[M]^+$ and $[M - H]^+$.

Solubility of the carboxylic acids. Acid **8** and thieno[3,2-*b*]thiophene-2-carboxylic acid dissolve only in DMSO and DMF and are sparingly soluble in EtOAc, approximately 100 mg per 4 mL. Two extractions with a volume of EtOAc equal to the aqueous phase remove inorganic salts efficiently. The latter also dissolves in $CHCl_3$/MeOH mixtures; three azeotropic co-evaporations from this solvent system remove water and residual EtOAc and markedly improve the quality of the ^1H spectrum (Figure S48).

Chromatography of the stannanes. Compounds **13** and **13′** decompose on untreated silica. Adding 3% Et_3N to the eluent suppresses this and reduces the protodestannylated by-product, ethyl thieno[3,2-*b*]thiophene-2-carboxylate, from 0.25 to 0.1 equiv starting material **7** (*R*f 0.48) and product **13** (R_f 0.51) are almost isopolar in EtOAc/hexanes 1:19 and cannot be separated by flash chromatography; this is inconsequential, because **7** is inert to the subsequent fluorination and is recovered unchanged. The stannylation must be stopped as soon as the solution turns black – continued heating consumes both **7** and **13** and generates a less polar by-product (R_f 0.70).

Drying of DPP 5. Compound **5** retains large amounts of solvent that cannot be removed by prolonged high-vacuum treatment (0.03 Torr, 50 °C, 4 h). Heating a 50 mg sample in an oven at 120 °C for 10 min reduced its mass by 28% and, by ^{1}H NMR, removed all solvent except a trace of methanol. This treatment was used before every alkylation.

Quench stoichiometry in the DPP synthesis. The acetic acid used to neutralize the sodium alkoxide must be limited to the equivalence calculated from the sodium charged. At approximately 1.5 equiv, visible loss of product occurs. Washing the collected solid with hot methanol and then water is sufficient; the acetone, ethyl acetate and hexane washes described in earlier procedures [S14–S17] introduce solvents that are difficult to remove from this poorly soluble product and were omitted.

Formation of the *O*-alkylation by-product 4′. Compound **4′** (*R*f 0.44, EtOAc/hexanes 1:9) begins to appear 2–4 h into the alkylation of **5** and cannot be removed by recrystallization. Stopping the reaction between 3 and 8 h, before **4′** accumulates, is essential. Dissolving the crude product in hot toluene and adding a small volume of ice-cold hexane, rather than cooling the toluene solution alone, gives a solid mixture containing **4** (R_f 0.38) together with by-products at R_f 0.16 and 0.08 that are readily resolved by a second chromatography; this raised the recovery of pure **4** from 23% to 42%. Filtrates were combined with subsequent batches for repurification.

Decomposition of 16 during deprotection. During the LiOH treatment of **16**, a broad band of unknown material fluorescing at 366 nm appeared on TLC, and the 235 nm absorption of the substrate disappeared. No such band was observed when the fluorine-free ester was subjected to identical conditions. After acidification the solution was concentrated without extraction; TLC of the residue (MeOH/CH_2Cl_2 3:7) showed multiple spots. Reversed-phase HPLC resolved three fractions (4–6, 6–10.5, and 14.5–17.5 min) accounting for 12 mg from a 12.4 mg charge, which by itself establishes that the recovered material is not pure **17**. The isolated solid resembled **16** in appearance and in its blue-green fluorescence at 366 nm, its ^{19}F spectrum showed only a singlet at δ −77.0 ppm attributable to residual triflate, and its measured $[M - H]^-$ deviated from the value calculated for **17** by more than 400 ppm. Co-spotting against **15** and thieno[3,2-*b*]thiophene-2-carboxylic acid (MeOH/CH_2Cl_2 2:8) showed it to be identical to neither, excluding dimerization and hydrodefluorination.

Purification of the dimeric diacid 15. After hydrolysis of **14** and acidification, TLC of the combined phases (MeOH/CH_2Cl_2 1:1) showed at least three components in the aqueous and organic layers. Recrystallization of the organic extract from hot methanol gave clean material in 31% recovery, mp 202–203 °C; chromatography on silica with MeOH/CH_2Cl_2 gave material of acceptable purity in the aromatic region but containing minor impurities elsewhere.

S6 Chromatographic data

Table S5: TLC *R*f values.

Compound	Eluent	R_f	Detection
3,4-dibromothiophene	EtOAc/hexanes 1:9	0.70	254 nm
**6**	EtOAc/hexanes 1:9	0.54	254 nm
**7**	EtOAc/hexanes 2:8	0.64	254 and 366 nm
**7**	EtOAc/hexanes 1:19	0.48	254 nm
**8**	MeOH/CH_2Cl_2 2:8	0.37	254 nm
**9**	MeOH/CH_2Cl_2 2:8	0.00–0.08	254 nm
**10**	CH_2Cl_2	0.54	366 nm
**13**	EtOAc/hexanes 1:19	0.51	254 nm
**13′**	EtOAc/hexanes 1:19	0.54	254 nm
**16**	EtOAc/hexanes 1:9	0.19	366 nm
**14**	EtOAc/hexanes 1:19	0.19	254 nm
ethyl thieno[3,2-*b*]thiophene-2-carboxylate	EtOAc/hexanes 2:8	0.64	254 nm
**15**	MeOH/CH_2Cl_2 3:7	0.42	254 nm
thieno[3,2-*b*]thiophene-2-carboxylic acid	MeOH/CH_2Cl_2 2:8	0.37	254 nm
**4**	EtOAc/hexanes 1:9	0.38	254 nm
**4′**	EtOAc/hexanes 1:9	0.44	254 nm
indan-1,3-dione	EtOAc/hexanes 2:8	0.34	254 nm
**18**	EtOAc/hexanes 2:8	0.55	254 nm

Table S6: HPLC methods. Normal-phase separations used a ZORBAX SIL column (9.4 × 250 mm, 5 µm); the reversed-phase separation used a CHEMCOSORB 7-ODS-H column (10 × 250 mm). All eluents were isocratic at 3 mL min^{-1} and filtered through 0.2 µm membranes before use.

Compound	Mode	Eluent	λ (nm)	Retention time (min)	Sample loading
**10**	normal phase	CH_2Cl_2	366	10.21	30 mg in CH_2Cl_2/hexanes 1:1 (1.0 mL) + $CHCl_3$ (1.0 mL); 20 µL injected
**13**	normal phase	EtOAc/hexanes 1:19	254	6.14	81.2 mg in CH_2Cl_2 (0.3 mL) + EtOAc/hexanes 1:19 (1.0 mL); 20 µL injected

**13′**	normal phase	EtOAc/hexanes 1:19	254	5.6–6.2	200 mg in EtOAc/hexanes 1:19 (0.8 mL)
**16**	normal phase	EtOAc/hexanes 1.5:8.5	366	9.40	200 mg in CH_2Cl_2 (1.8 mL); 30 mg undissolved
**17** (mixture)	reverse phase	MeCN/H_2O 1:1	366	4–6, 6–10.5, 14.5–17.5	12.6 mg in MeOH (1.0 mL)

Because **16** is only sparingly soluble in the mobile phase, part of every sample remained undissolved; the recoveries quoted for HPLC-purified **16** are therefore lower bounds and are not yields.

S7 Spectroscopic basis for the structure of 16

The product of the silver-mediated reaction is assigned as the α-fluorothiophenone ethyl 5-fluoro-6-oxo-5,6-dihydrothieno[3,2-*b*]thiophene-2-carboxylate rather than the aryl fluoride ethyl 6-fluorothieno[3,2-*b*]thiophene-2-carboxylate. Three independent lines of evidence converge, and each excludes the aromatic alternative on its own.

Table S7: Observed data for **16** against the values expected for the three structural possibilities.

Observable	Observed	Aryl fluoride	Vinyl fluoride (endocyclic C5=C6)	α-Fluoroketone (sp^3 C5)
Molecular formula (HREIMS)	$C_9H_7FO_3S_2$, 245.9822	$C_9H_7FO_2S_2$, 229.9871 –not detected	$C_9H_7FO_3S_2$ ✓	$C_9H_7FO_3S_2$ ✓
J(H,F)	52.5 Hz	≤3.4 Hz for 3-fluorothiophene [S18,S19]	ca. 20 Hz – substituents are necessarily *cis* in a five-membered ring	45–55 Hz for ^2J in H–C–F ✓
δ_F	−167.9	−131.0 for 3-fluorothiophene [S18,S19]	−90 to −130 for vinylic F	−165 to −200 for secondary alkyl F ✓
Carbon bearing F	δ 98.2, protonated (DEPT), d, ^1J(C,F) = 232.1 Hz	quaternary, δ ≈ 158	quaternary	protonated and directly F-bonded ✓
Ring carbonyl	δ 186.7, d, *J* = 13.5 Hz	absent	absent	ketone with ^2J(C,F) 15–25 Hz ✓
Ester carbonyl	δ 160.7	δ 162.2 in **7**, 162.4 in **15**	–	✓

The ^{13}C NMR spectrum is decisive on its own. A one-bond coupling of 232.1 Hz on a carbon that DEPT identifies as protonated can only mean that hydrogen and fluorine occupy the same carbon: an sp^3 C(H)F unit. One-bond C–F couplings increase steeply with the electronegativity of the other substituents on that carbon –158 Hz in CH_3F against about 235 Hz in CH_2F_2 –and C5 carries a ring sulfur and an adjacent

carbonyl in addition to hydrogen and fluorine, so a value near 230 Hz is what this particular carbon requires. Both aromatic alternatives are excluded by the same datum: an aromatic C–F carbon is quaternary and resonates near δ 158, while an aromatic CH *ortho* to fluorine would show a two-bond coupling of 20–27 Hz.

The molecular formula independently confirms the composition. The product carries one oxygen more than the aryl fluoride while retaining the same seven hydrogens, and therefore the same six degrees of unsaturation. Of those seven hydrogens, five belong to the ethyl ester, leaving two on the ring system – the same number carried by the stannane **13**, in which H occupies C3 and C5 and $SnMe_3$ occupies C6. Because fluorine and one ring proton are mutually coupled and hence share a carbon, fluorine cannot be at C6; yet C6 must acquire a substituent on losing tin, and oxygen is the only atom available. Oxygen at C6 and fluorine at C5 is the only arrangement consistent with the formula.

The signal at δ 186.7, reported in earlier processing of these data as the ester carbonyl with a five-bond coupling of 13.5 Hz, is the ring ketone. No ester carbonyl in this series resonates there – –those of **7**, **13**, **13′** and **14** all fall between δ 162.2 and δ 162.8 – –and a five-bond C–F coupling of that magnitude has no precedent, whereas a two-bond coupling between a carbonyl and an adjacent C–F of 13.5 Hz is entirely ordinary.

S8 Copies of spectra

Spectra are reproduced from the original characterization data. Compound numbers follow the main text.

Figure	Compound	Spectrum
S1	**5**	^{1}H NMR (500 MHz, DMF-d_7)
S2	**6**	^{1}H NMR (500 MHz, $CDCl_3$)
S3	**6**	^{13}C NMR (125 MHz, $CDCl_3$)
S4	**6**	HRESIMS
S5	**6**	LREIMS
S6	**6**	HREIMS
S7	**7**	^{1}H NMR (500 MHz, $CDCl_3$)
S8	**7**	^{13}C NMR (125 MHz, $CDCl_3$)
S9	**7**	HRESIMS
S10	**8**	^{1}H NMR (500 MHz, DMSO-d_6)
S11	**8**	^{13}C NMR (125 MHz, DMSO-d_6)
S12	**8**	HRESIMS
S13	**10**	Normal-phase HPLC trace (CH_2Cl_2, 3 mL min^{-1}, 366 nm)
S14	**10**	^{1}H NMR (500 MHz, $CDCl_3$), HPLC-purified

S15	**10**	^{13}C NMR (125 MHz, $CDCl_3$), HPLC-purified
S16	**10**	HRESIMS
S17	**10**	LREIMS
S18	**10**	HREIMS
S19	indan-1,3-dione	^{1}H NMR (500 MHz, $CDCl_3$)
S20	**11** (attempted)	^{1}H NMR of the crude product from Me_6Sn_2/$Pd(PPh_3)_2Cl_2$ in 1,4-dioxane
S21	**11** (attempted)	^{1}H NMR of the crude product from Me_6Sn_2/$Pd(PPh_3)_4$ in THF
S22	**18**	^{1}H NMR (500 MHz, $CDCl_3$)
S23	**18**	^{1}H NMR, aromatic region expanded
S24	**13**	Normal-phase HPLC trace (EtOAc/hexanes 1:19, 254 nm)
S25	**13**	^{1}H NMR (500 MHz, $CDCl_3$), HPLC-purified
S26	**13**	^{13}C NMR (125 MHz, $CDCl_3$), HPLC-purified
S27	**13**	LREIMS
S28	**13**	HREIMS, $[M]^+$ region
S29	**13**	HREIMS, $[M - CH_3]^+$ region
S30	**13′**	Normal-phase HPLC trace (EtOAc/hexanes 1:19, 254 nm)
S31	**13′**	^{1}H NMR (500 MHz, $CDCl_3$), HPLC-purified
S32	**13′**	^{13}C NMR (125 MHz, $CDCl_3$), HPLC-purified
S33	**13′**	LREIMS
S34	**13′**	HREIMS, $[M]^+$ region
S35	**13′**	HREIMS, $[M - C_4H_9]^+$ region
S36	**14**	^{1}H NMR (500 MHz, $CDCl_3$)
S37	**14**	^{13}C NMR (125 MHz, $CDCl_3$)
S38	**14**	LREIMS
S39	**14**	HREIMS
S40	**16**	^{1}H NMR (500 MHz, $CDCl_3$), from **13**, before HPLC
S41	**16**	^{19}F NMR (470 MHz, $CDCl_3$), from **13**, before HPLC
S42	**16**	LREIMS, from **13**
S43	**16**	HREIMS, from **13**, showing $[M]^+$ and the absence of the aryl fluoride ion
S44	**16**	Normal-phase HPLC trace (EtOAc/hexanes 1.5:8.5, 366 nm), from **13′**
S45	**16**	^{1}H NMR (500 MHz, $CDCl_3$), HPLC-purified, from **13′**
S46	**16**	^{13}C NMR (125 MHz, $CDCl_3$), HPLC-purified, from **13′**
S47	**16**	^{19}F NMR (470 MHz, $CDCl_3$), HPLC-purified, from **13′**
S48	thieno[3,2-*b*]thiophene-2-carboxylic acid	^{1}H NMR (500 MHz, CD_3OD/$CDCl_3$ 2:1)
S49	**4**	^{1}H NMR (500 MHz, $CDCl_3$)
S50	**4**	^{13}C NMR (125 MHz, $CDCl_3$)
S51	**4**	HRESIMS
S52	**4′**	^{1}H NMR (500 MHz, $CDCl_3$)
S53	**15**	^{1}H NMR (400 MHz, DMSO-d_6), recrystallized from MeOH
S54	**15**	^{1}H NMR (400 MHz, DMSO-d_6), after chromatography
S55	**17** (attempted)	^{1}H NMR of the crude acidified reaction mixture
S56	**17** (attempted)	^{1}H NMR after flash chromatography
S57	**17** (attempted)	^{19}F NMR after flash chromatography, showing only the triflate singlet at δ −77.0
S58	**17** (attempted)	HRESIMS after flash chromatography
S59	**17** (attempted)	Reverse-phase HPLC trace ($MeCN/H_2O$ 1:1, 366 nm)
S60	**17** (attempted)	^{1}H NMR of HPLC fraction 14-1

S61	**17** (attempted)	^{1}H NMR of HPLC fraction 14-1, aromatic region expanded
S62	**17**, **15**, thieno[3,2-*b*]thiophene-2-carboxylic acid	TLC co-spotting comparison ($MeOH/CH_2Cl_2$ 2:8)
S63	**10** / **11** (attempted)	Stacked ^{1}H NMR spectra: (a) **10**; (b) crude product from $Me_6Sn_2/Pd(PPh_3)_2Cl_2$; (c) crude product from $Me_6Sn_2/Pd(PPh_3)_4$. The C6 methylene singlet of **10** near δ 3.5 is absent in (b) and (c)
S64	F-TEDA-PF_6	^{1}H NMR (acetone-d_6)
S65	F-TEDA-PF_6	^{19}F NMR (acetone-d_6)

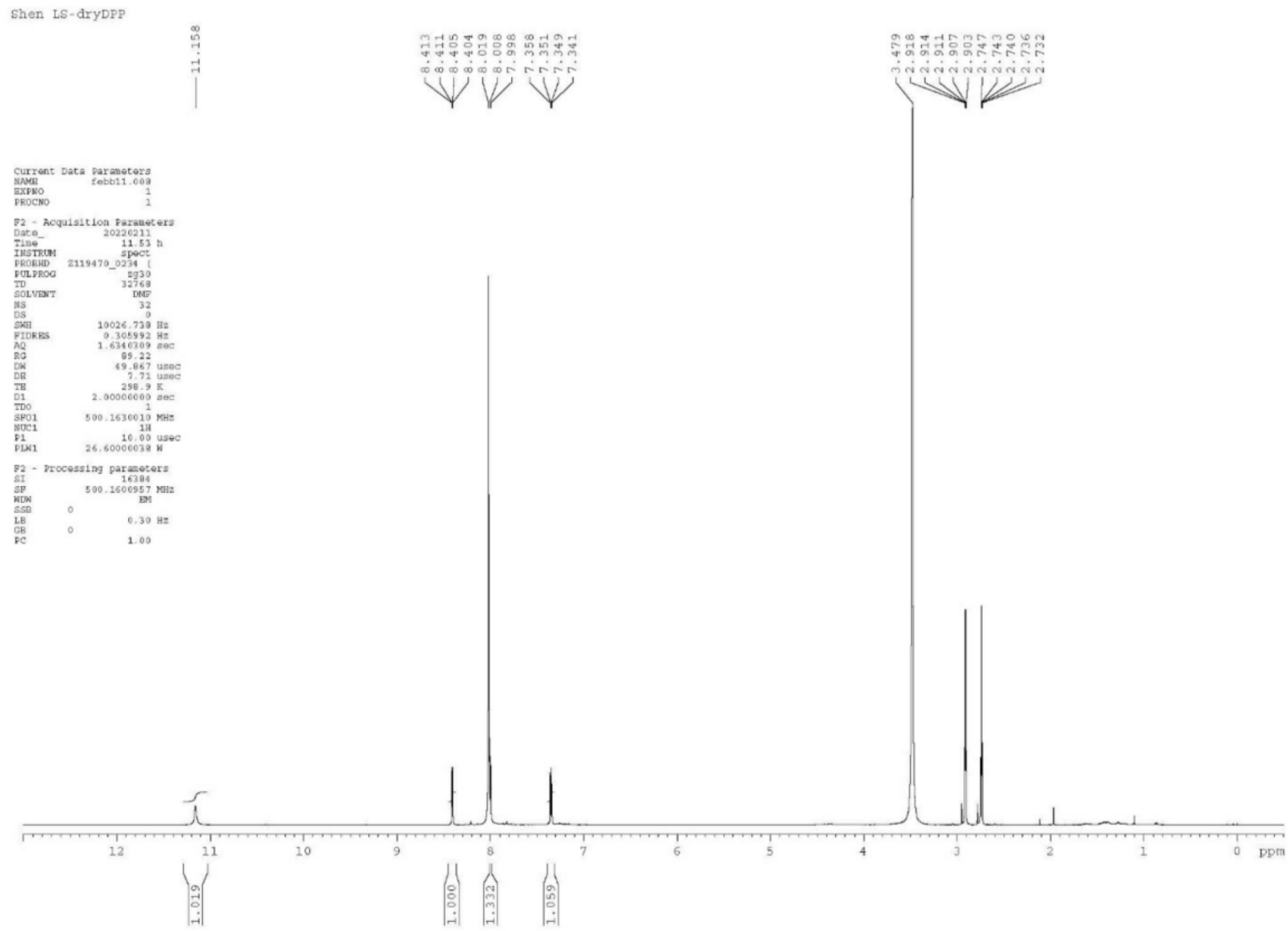

Figure S1: 5 — ^{1}H NMR (500 MHz, DMF-d_7).

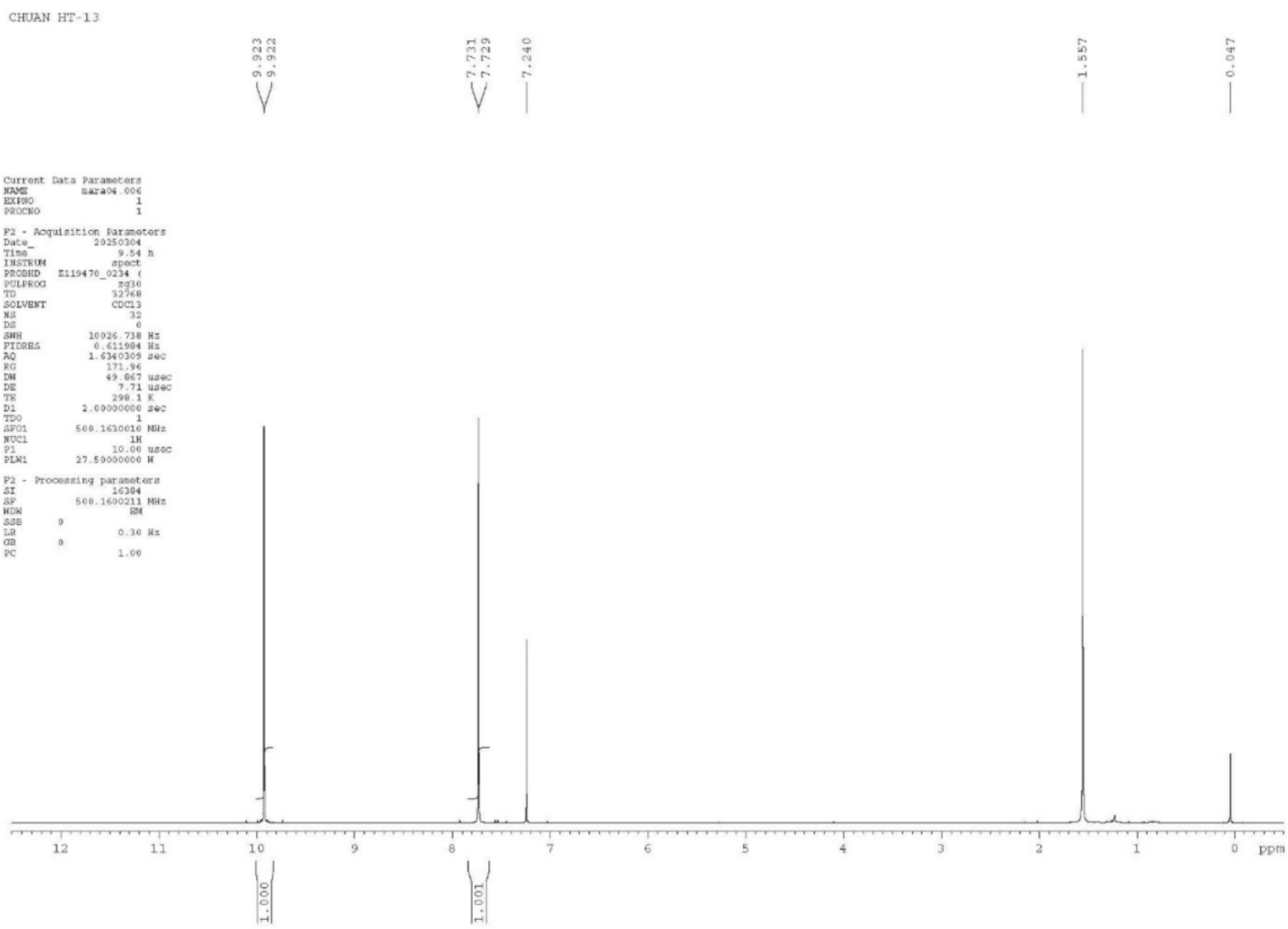

Figure S2: 6 — ^{1}H NMR (500 MHz, $CDCl_3$).

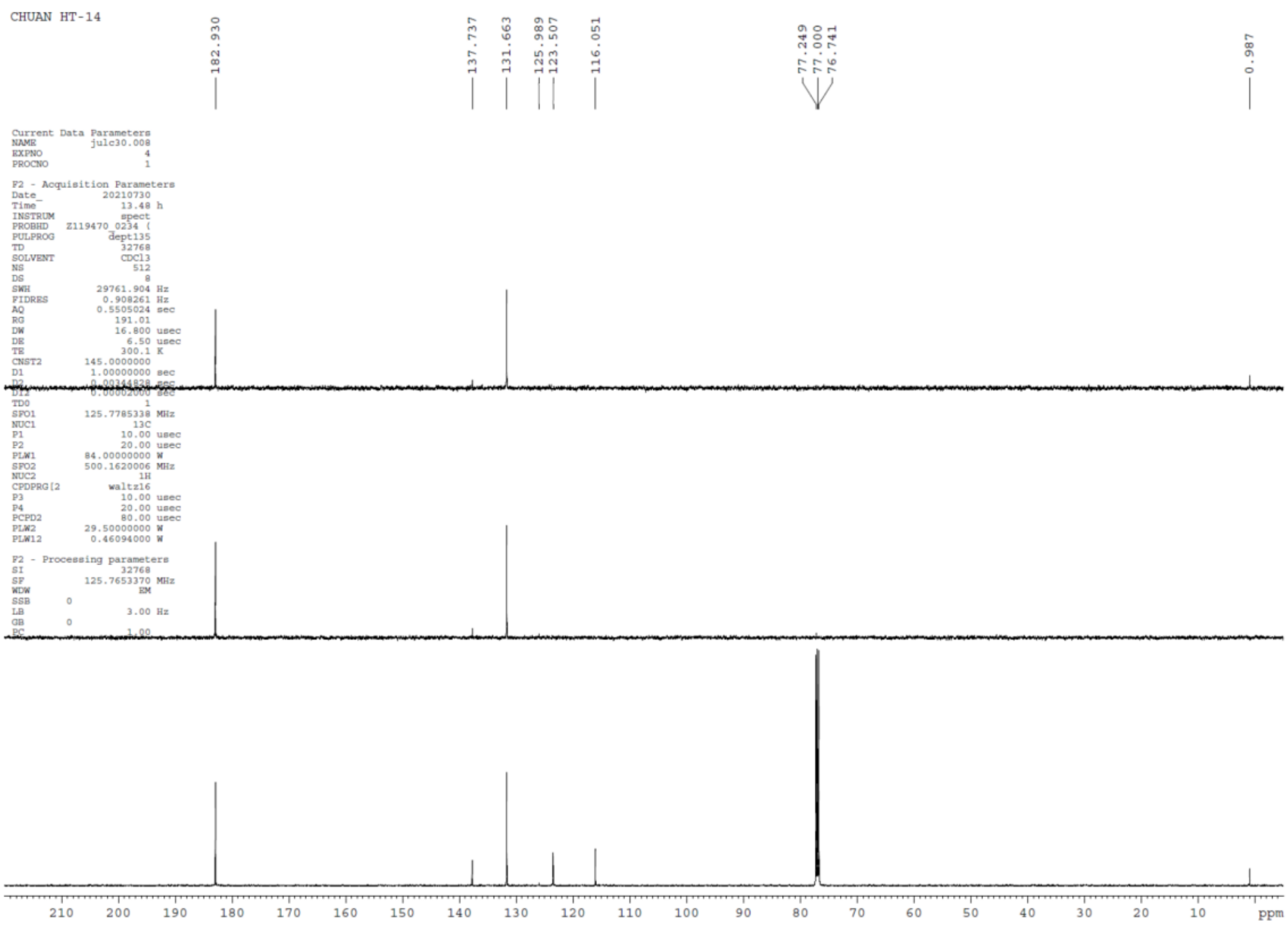

Figure S3: 6 — ^{13}C NMR (125 MHz, $CDCl_3$).

Display Report

Analysis Info

Analysis Name	D:\Data\nctu service\data\2024\20241227\CHUAN HT-13_GD4_01_45383.d	Acquisition Date	12/27/2024 3:29:15 PM
Method	Small molecule 200-1500.m	Operator	NCTU
Sample Name	CHUAN HT-13	Instrument	impact HD 1819696.00164
Comment			

Acquisition Parameter

Source Type	ESI	Ion Polarity	Positive	Set Nebulizer	1.0 Bar
Focus	Active	Set Capillary	4500 V	Set Dry Heater	200 °C
Scan Begin	200 m/z	Set End Plate Offset	-500 V	Set Dry Gas	6.0 l/min
Scan End	1500 m/z	Set Charging Voltage	2000 V	Set Divert Valve	Waste
		Set Corona	0 nA	Set APCI Heater	0 °C

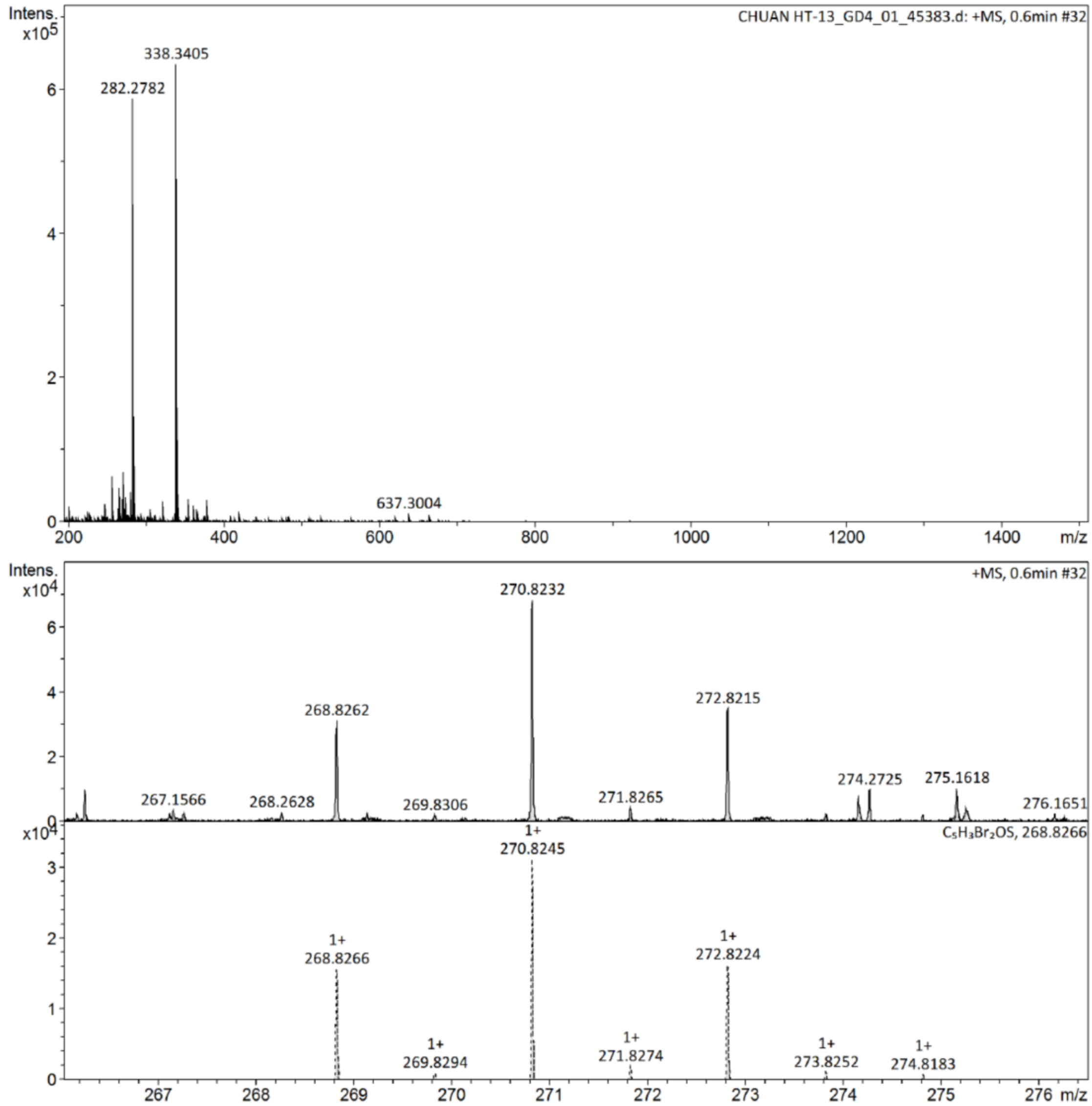

Figure S4: 6 — HRESIMS.

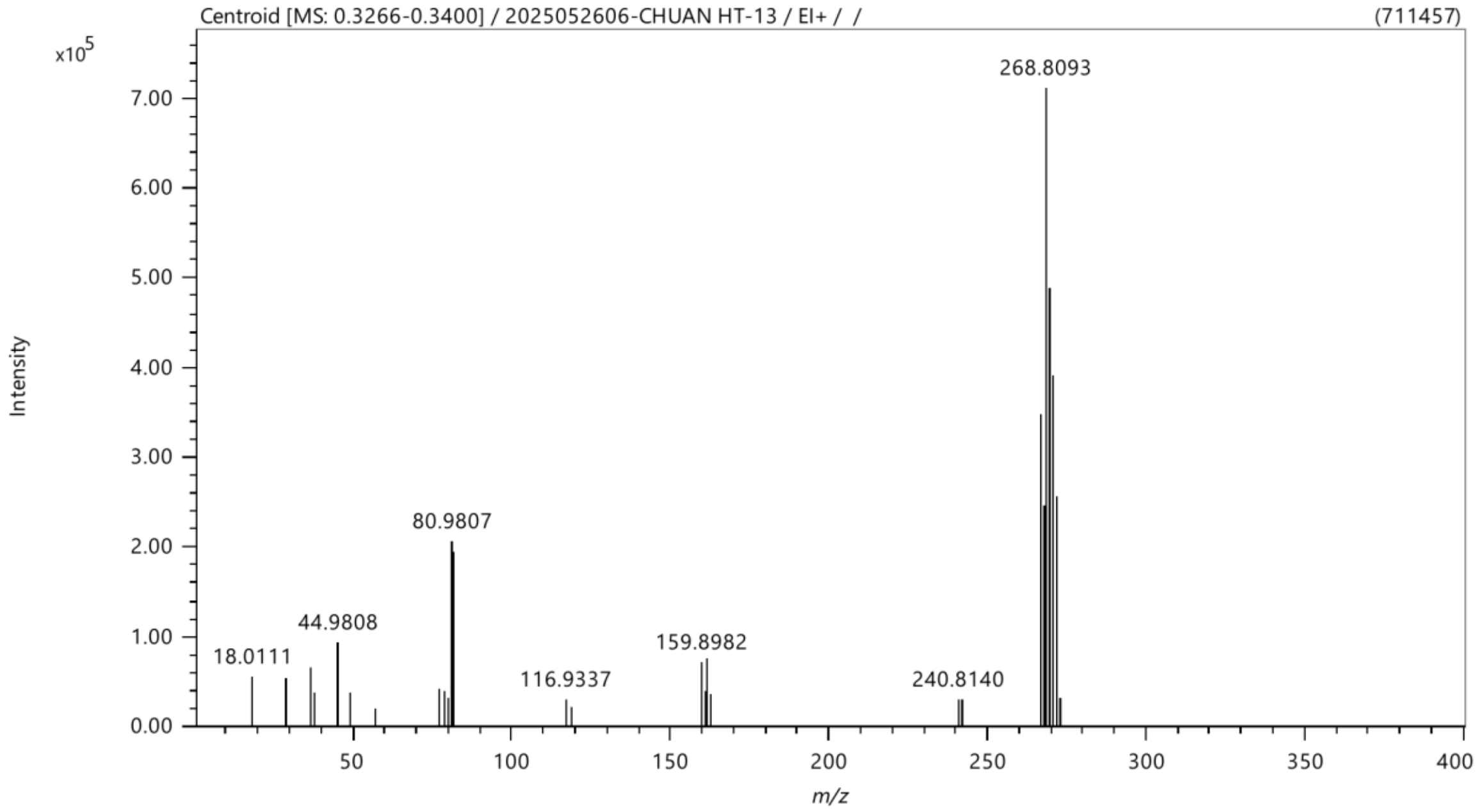

Figure S5: 6 — LREIMS.

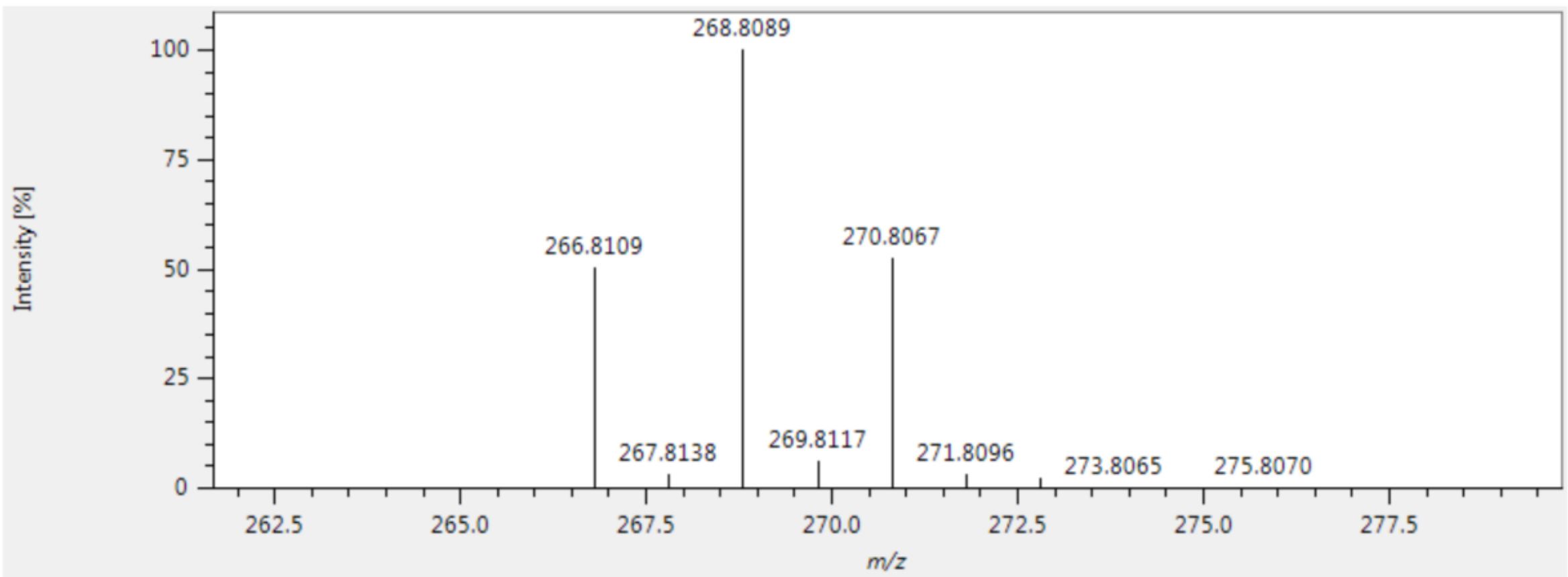

Mass	Intensity	Formula	Calculated Mass	Mass Difference [mDa]	Mass Difference [ppm]	DBE
266.81091	348257.38	C5 H O S Br2	266.81094	-0.03	-0.09	4.5

Figure S6: 6 — HREIMS.

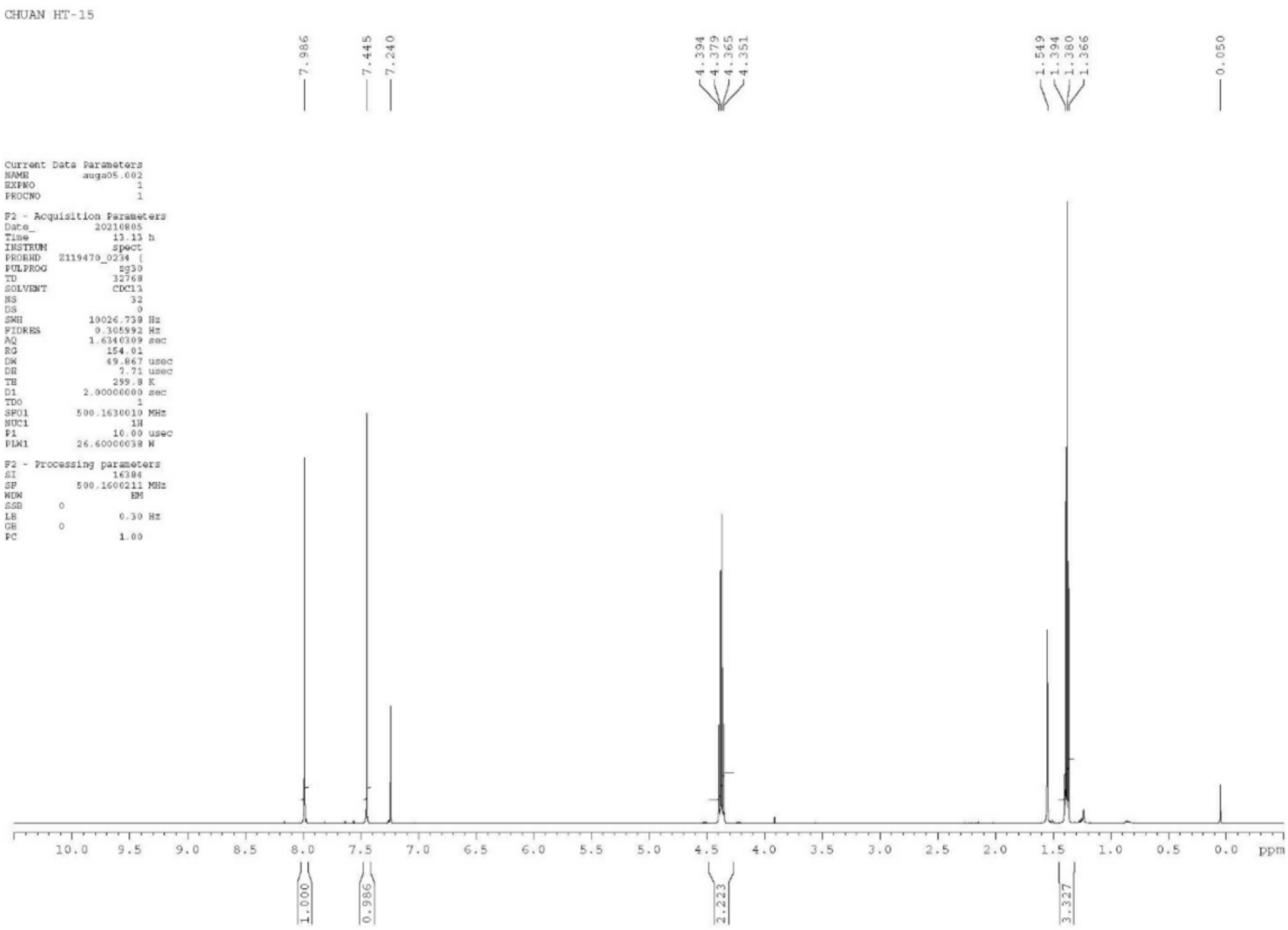

Figure S7: 7 — ^{1}H NMR (500 MHz, $CDCl_3$).

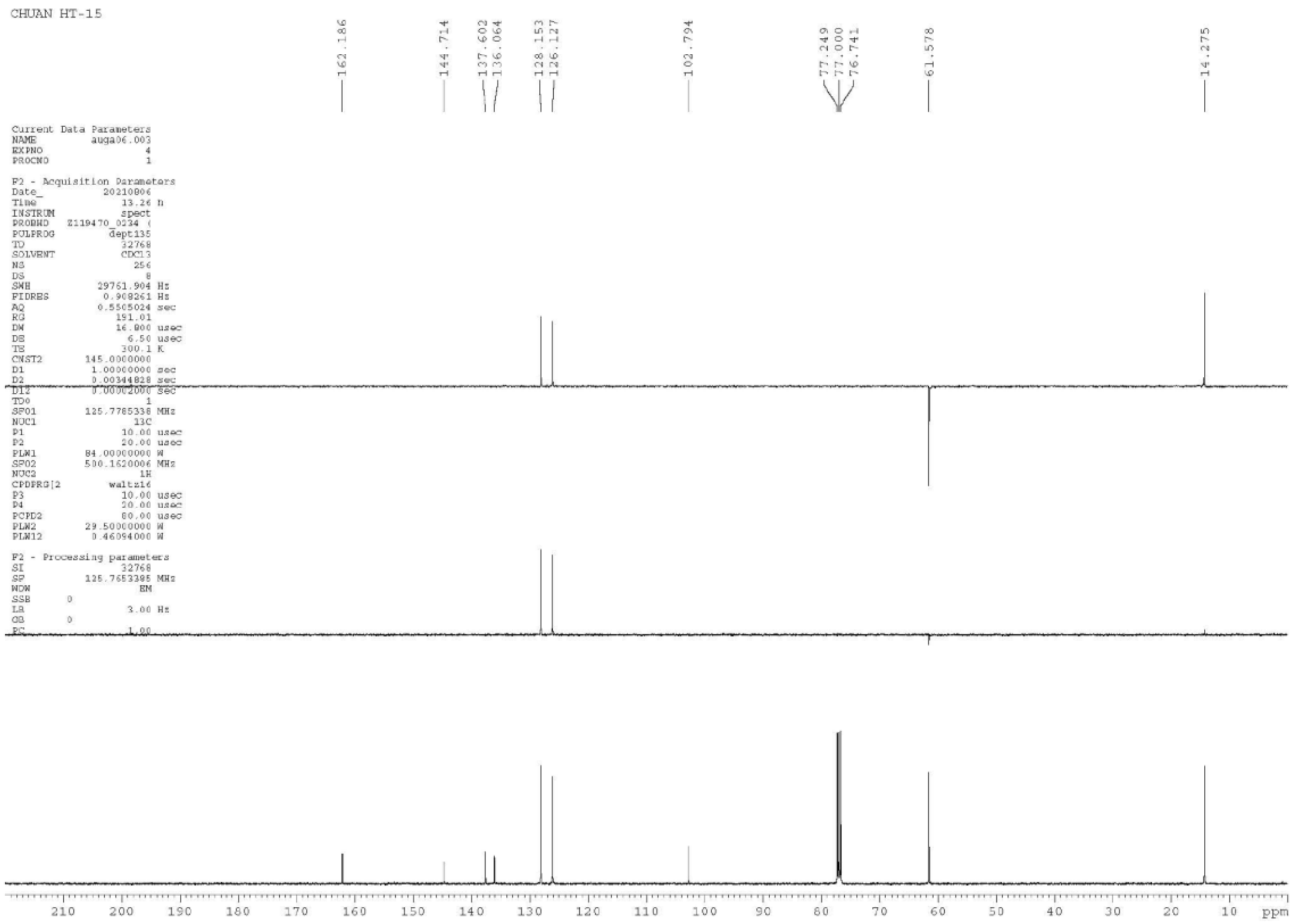

Figure S8: 7 — ^{13}C NMR (125 MHz, $CDCl_3$).

Display Report

Analysis Info

Analysis Name	D:\Data\nctu service\data\2024\20240920\CHUAN-HT-15_GC7_01_43824.d	Acquisition Date	9/20/2024 1:29:51 PM
Method	Small molecule.m	Operator	NCTU
Sample Name	CHUAN-HT-15	Instrument	impact HD 1819696.00164
Comment			

Acquisition Parameter

Source Type	ESI	Ion Polarity	Positive	Set Nebulizer	1.0 Bar
Focus	Active	Set Capillary	4500 V	Set Dry Heater	200 °C
Scan Begin	50 m/z	Set End Plate Offset	-500 V	Set Dry Gas	6.0 l/min
Scan End	1500 m/z	Set Charging Voltage	2000 V	Set Divert Valve	Waste
		Set Corona	0 nA	Set APCI Heater	0 °C

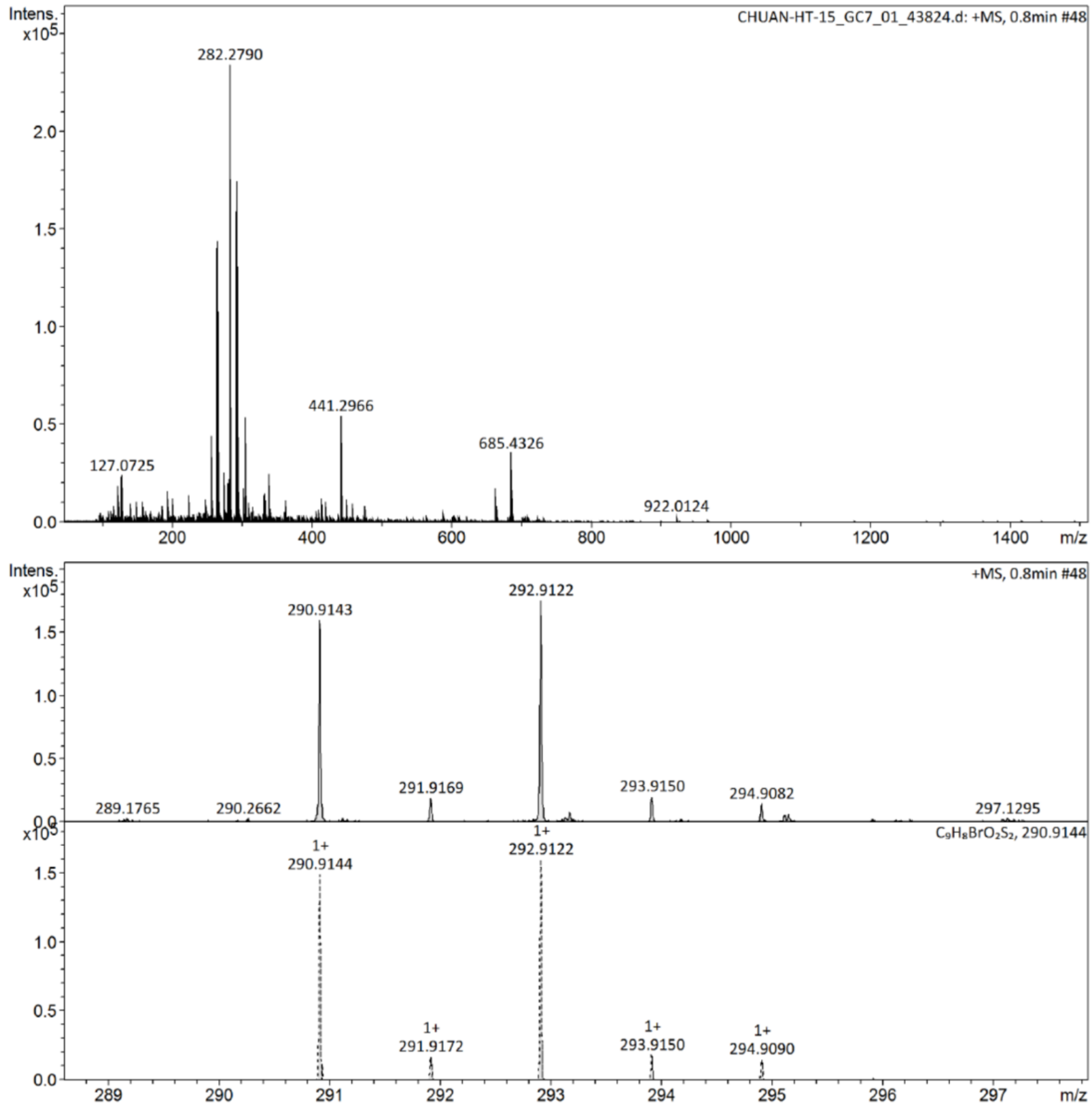

Figure S9: 7 — HRESIMS.

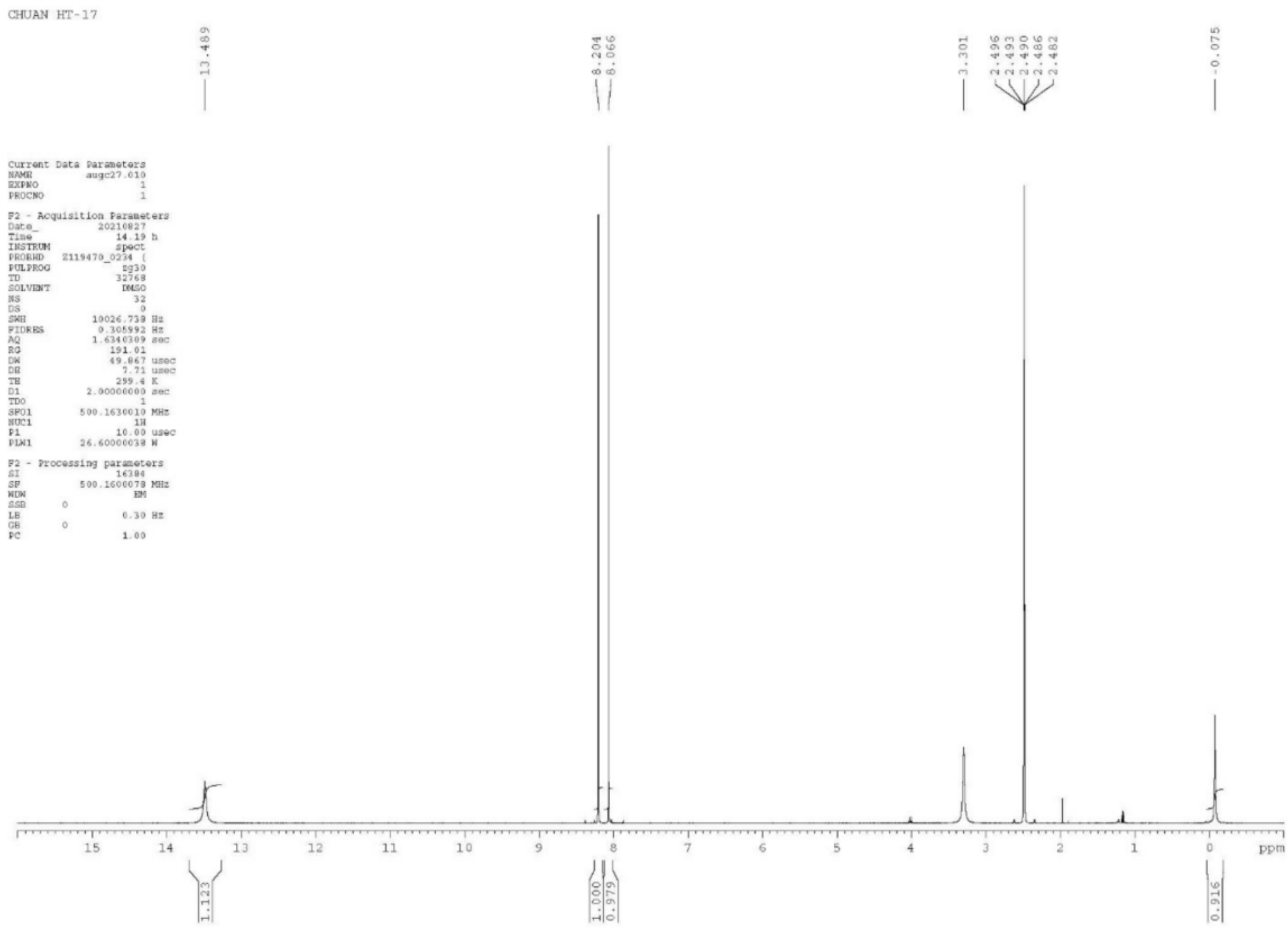

Figure S10: 8 — ^{1}H NMR (500 MHz, DMSO-d_6).

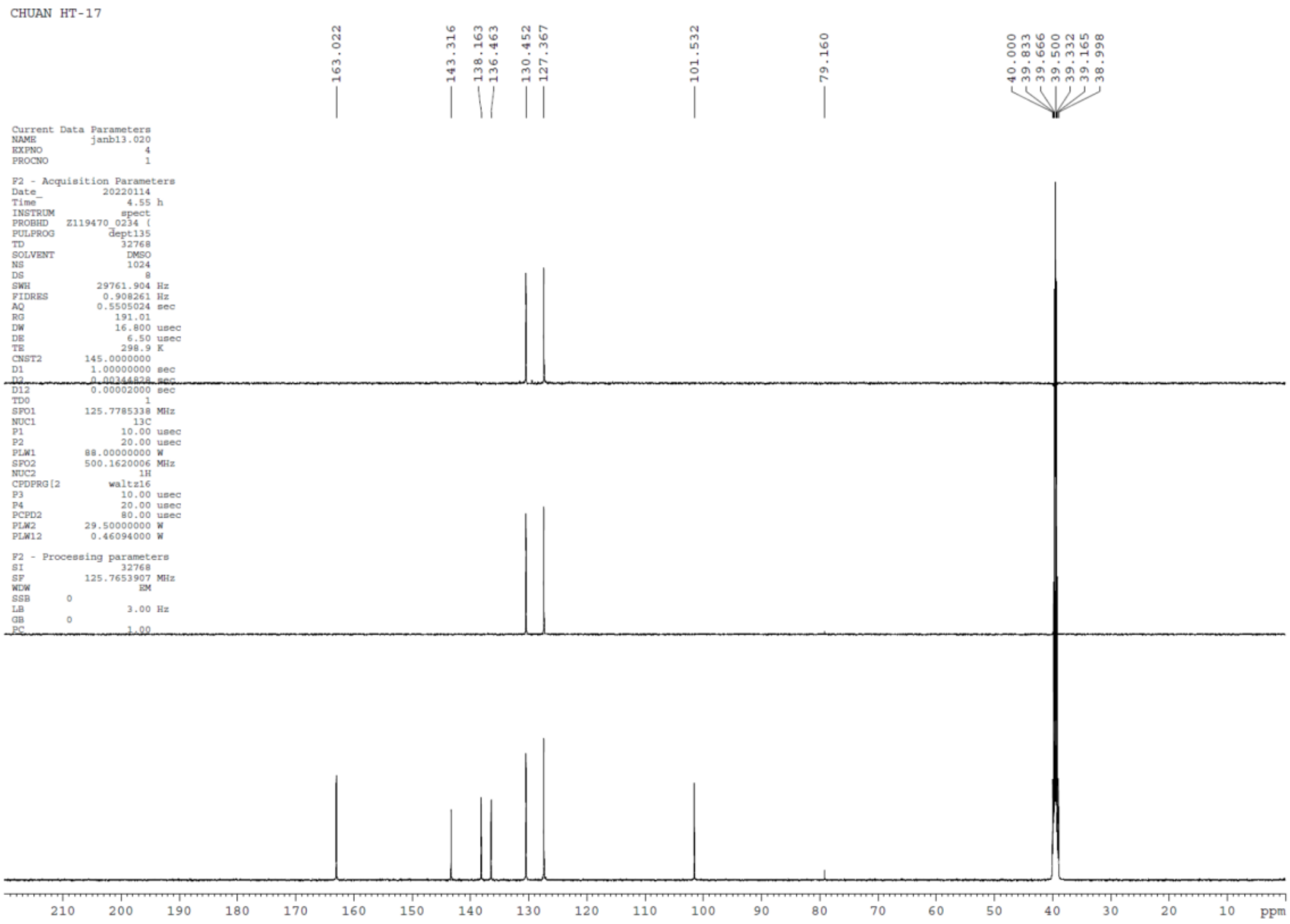

Figure S11: 8 — ^{13}C NMR (125 MHz, DMSO-d_6).

Data:CHUAN HT-17-
Comment:
Description:
Ionization Mode:ESI-
History:Average(MS[1] 0.29..0.34)

Acquired:1/10/2022 2:51:15 PM
Operator:AccuTOF
m/z Calibration File:2021201-TFANa_...
Created:1/10/2022 3:39:57 PM
Created by:AccuTOF

Charge number:1 Tolerance:300.00[ppm], 250.00 .. 250.... Unsaturation Number:-100.0 .. 200.0 (...
Element:^{12}C:7 .. 7, ^{1}H:0 .. 4, ^{79}Br:1 .. 1, ^{23}Na:0 .. 1, ^{16}O:2 .. 2, ^{32}S:2 .. 2

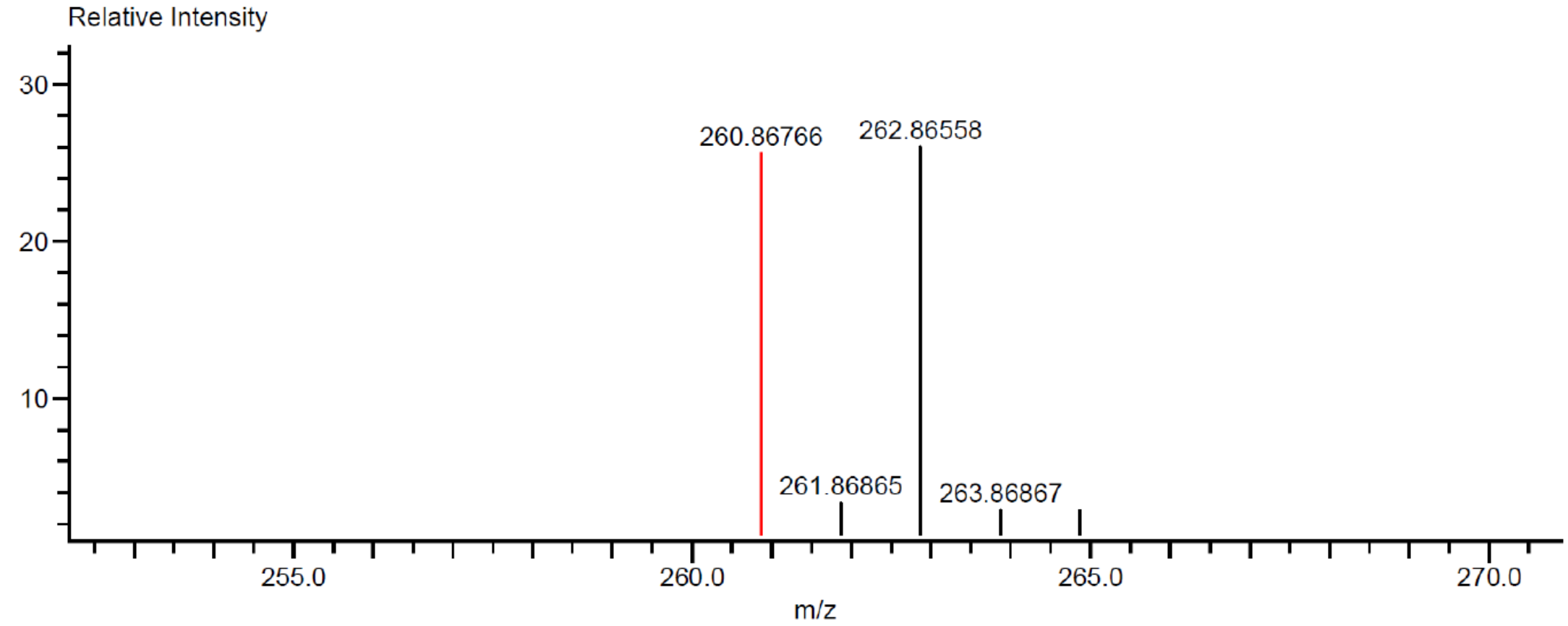

Mass	Intensity	Calc. Mass	Mass Difference [mDa]	Mass Difference [ppm]	Possible Formula
260.86766	9904.58	260.86796	-0.30	-1.14	$^{12}C_7{}^{1}H_2{}^{79}Br_1{}^{16}O_2{}^{32}S_2$

Figure S12: 8 — HRESIMS.

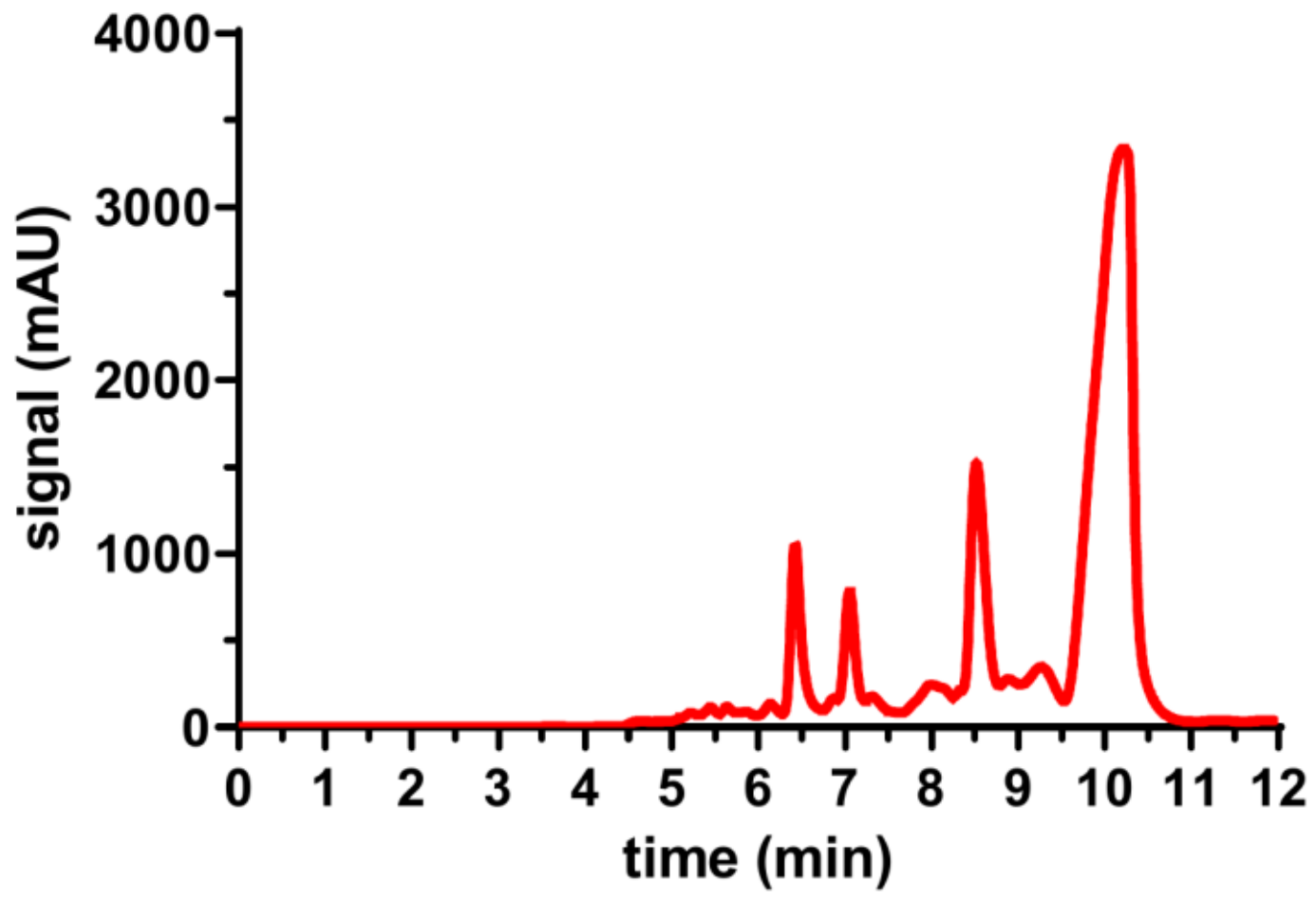

Figure S13: 10 — normal-phase HPLC trace (CH_2Cl_2, 3 mL min^{-1}, 366 nm).

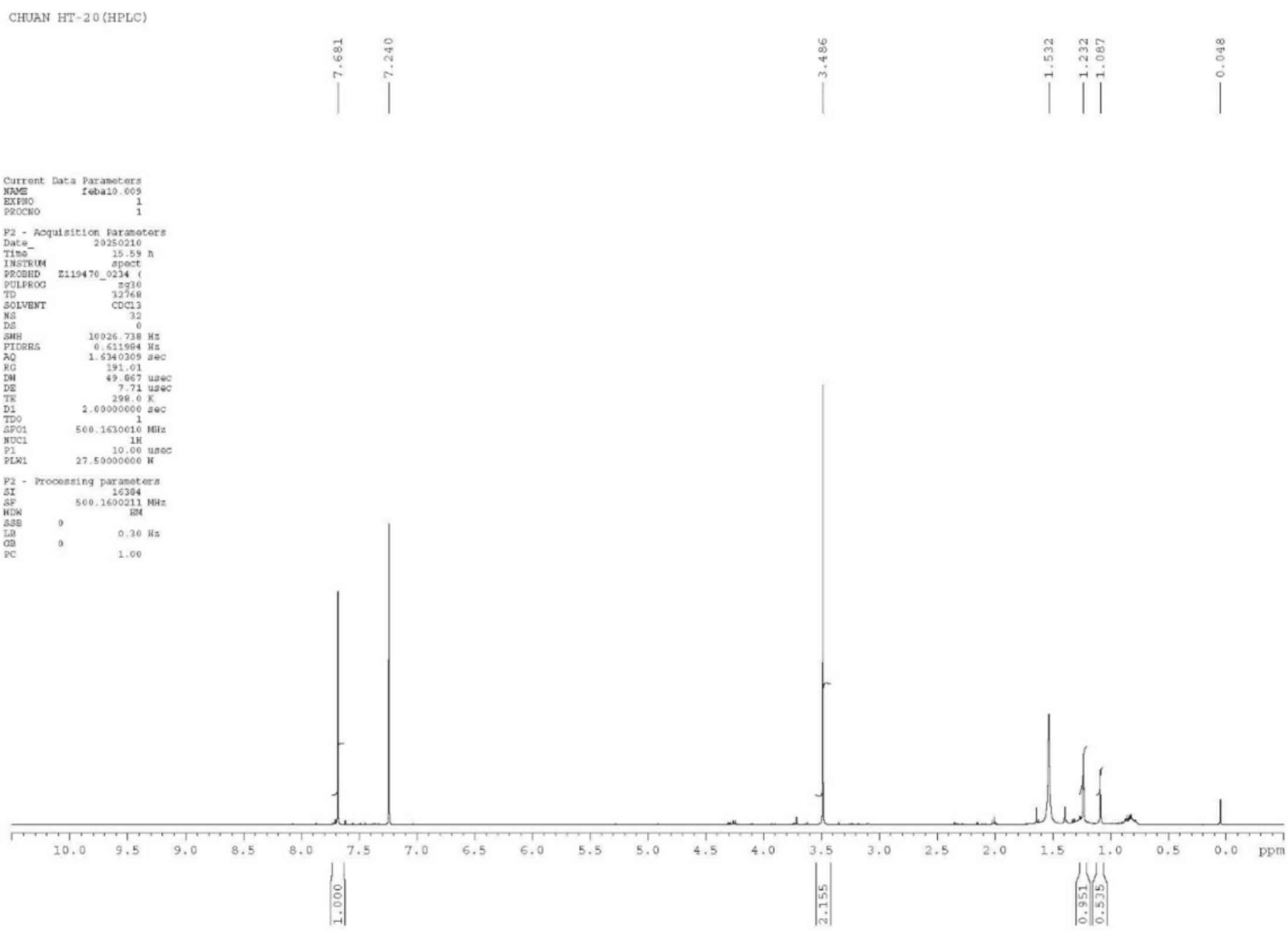

Figure S14: 10 — ^{1}H NMR (500 MHz, $CDCl_3$), HPLC-purified.

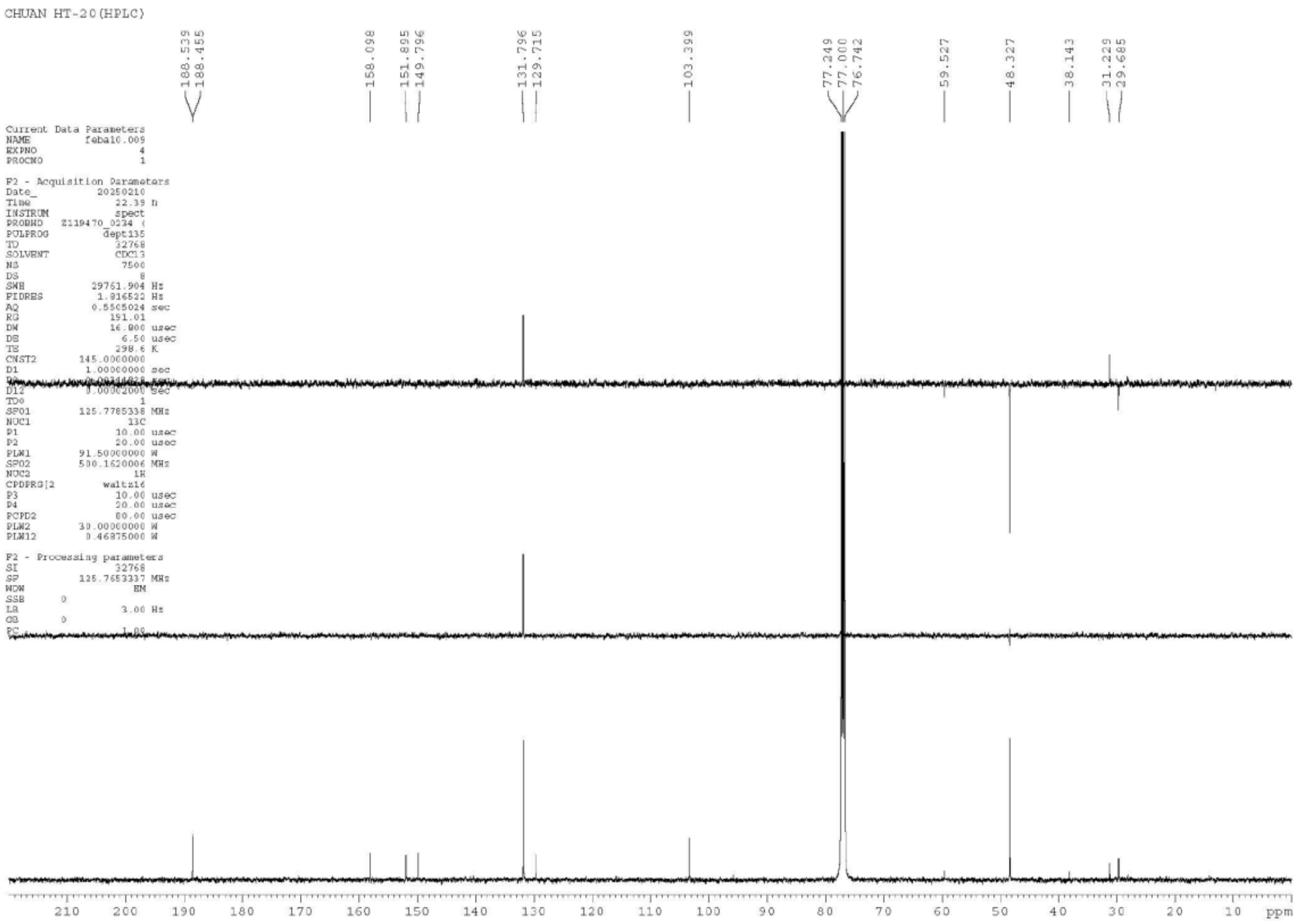

Figure S15: 10 — ^{13}C NMR (125 MHz, $CDCl_3$), HPLC-purified.

Display Report

Analysis Info

Analysis Name	D:\Data\nctu service\data\2025\20250107\CHUAN HT-20_GA1_01_45517.d	Acquisition Date	1/7/2025 12:04:03 PM
Method	Small molecule.m	Operator	NCTU
Sample Name	CHUAN HT-20	Instrument	impact HD 1819696.00164
Comment			

Acquisition Parameter

Source Type	ESI	Ion Polarity	Positive	Set Nebulizer	1.0 Bar
Focus	Active	Set Capillary	4500 V	Set Dry Heater	200 °C
Scan Begin	50 m/z	Set End Plate Offset	-500 V	Set Dry Gas	6.0 l/min
Scan End	1500 m/z	Set Charging Voltage	2000 V	Set Divert Valve	Waste
		Set Corona	0 nA	Set APCI Heater	0 °C

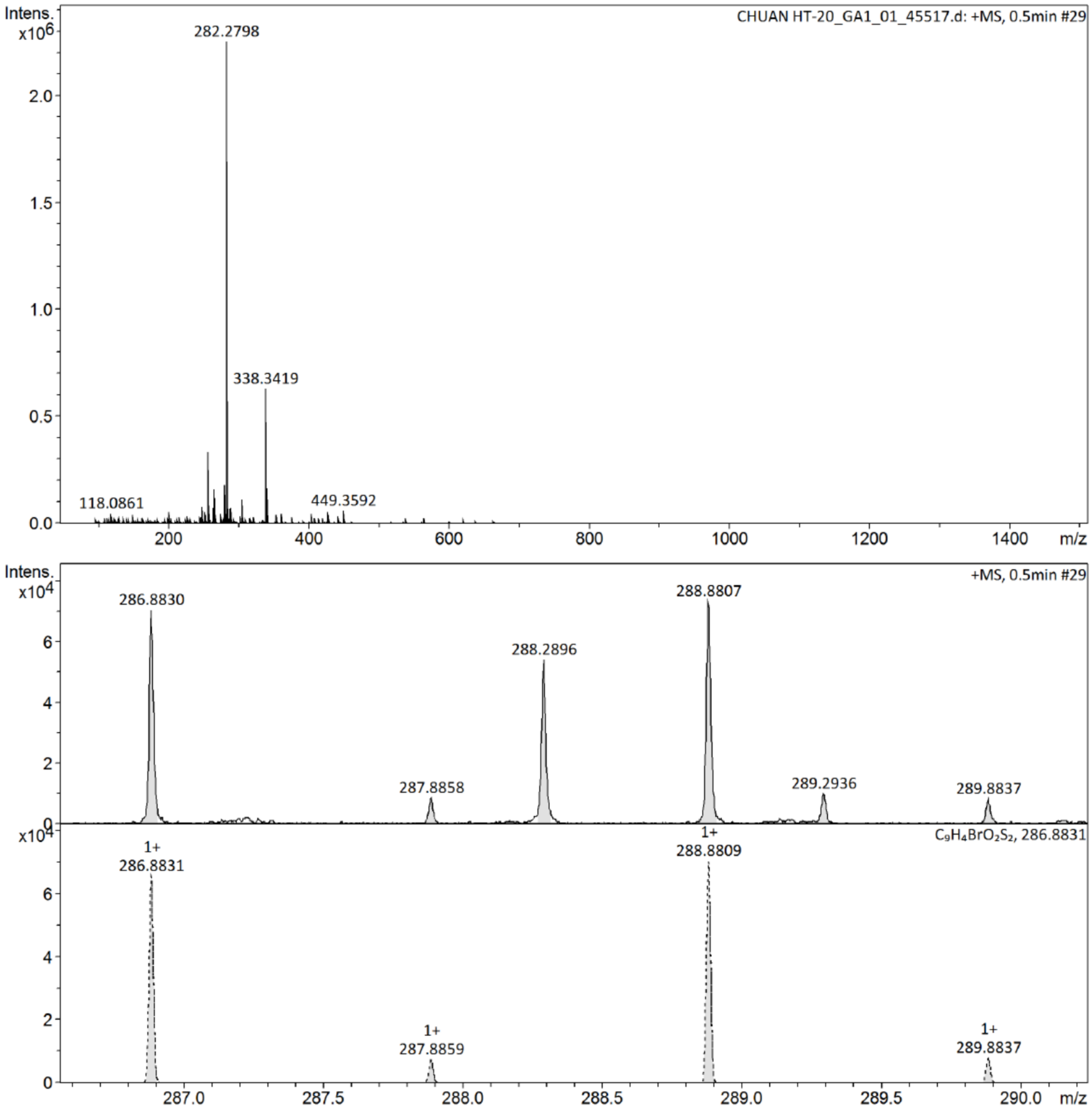

Figure S16: 10 — HRESIMS.

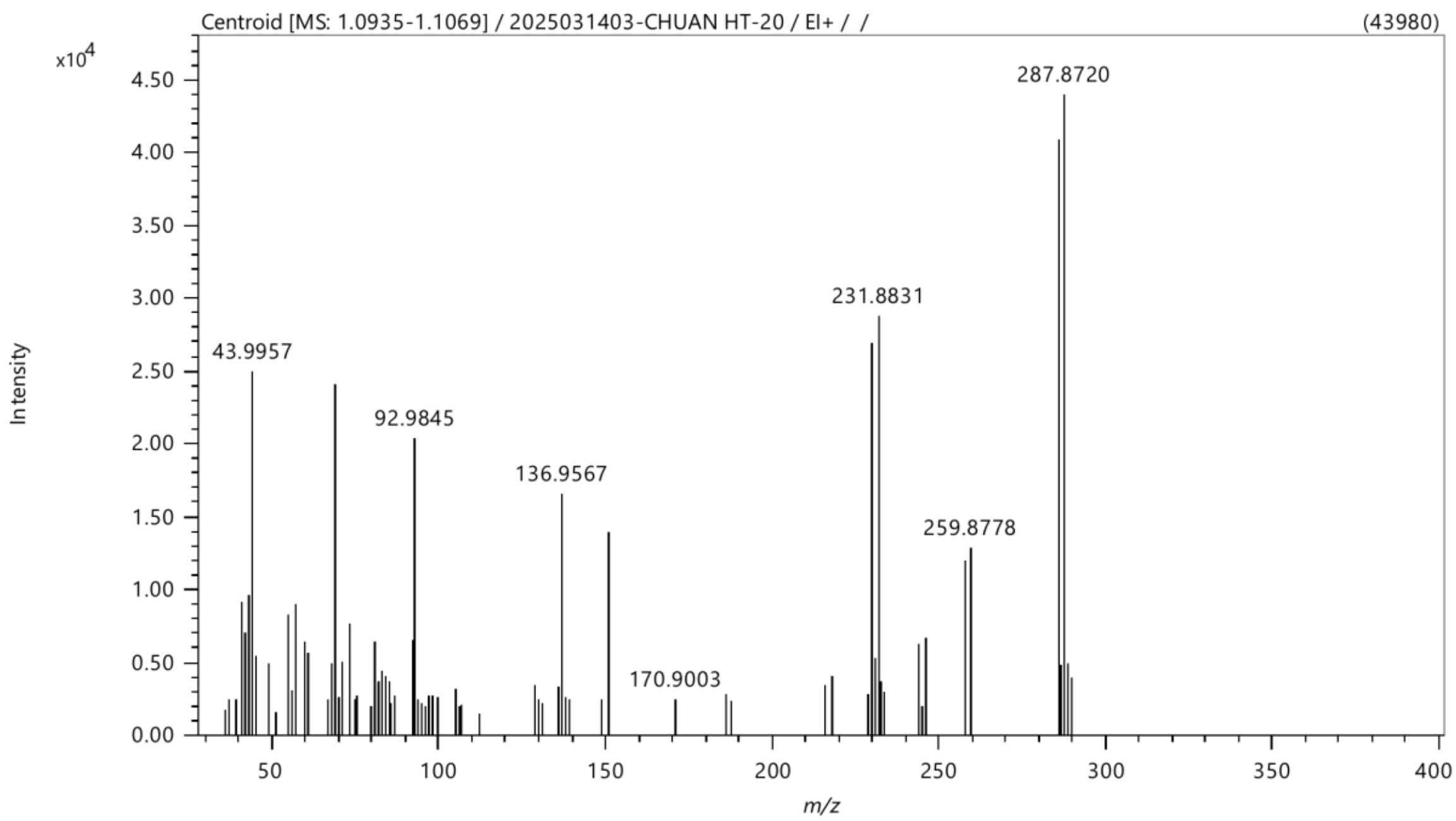

Figure S17: 10 — LREIMS.

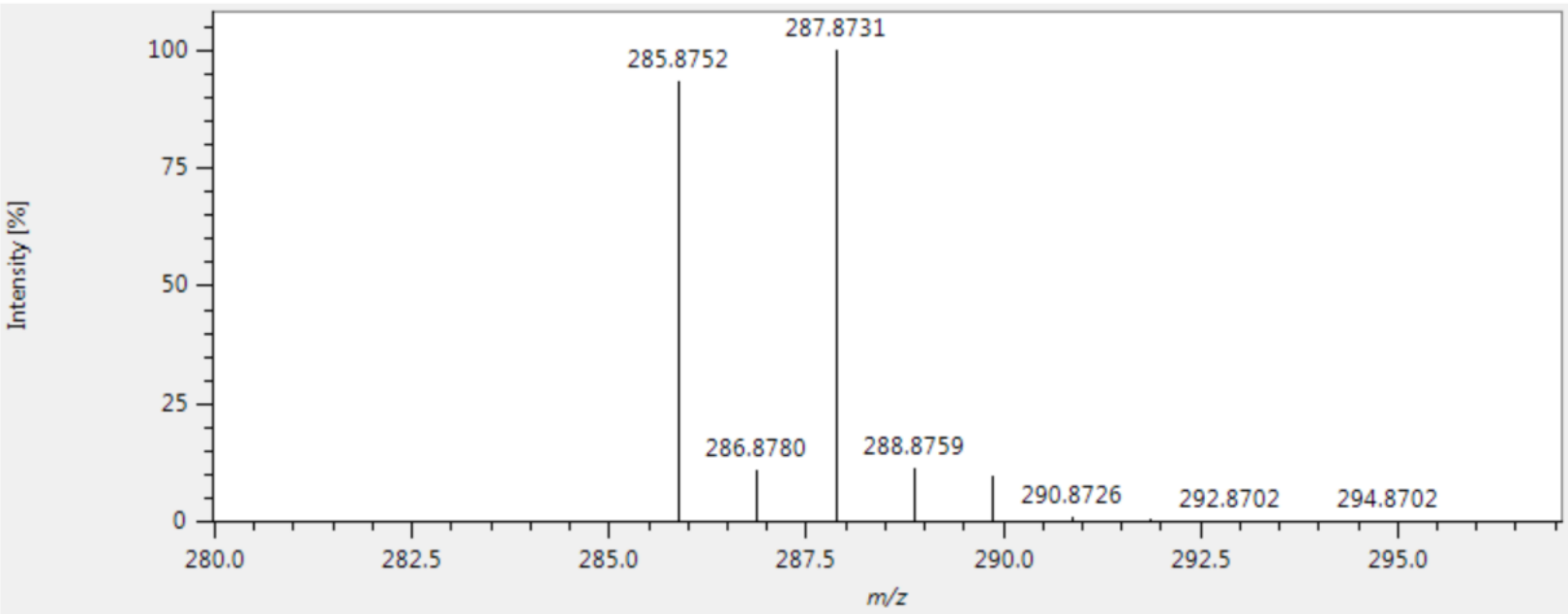

Mass	Intensity	Formula	Calculated Mass	Mass Difference [mDa]	Mass Difference [ppm]	DBE
285.87443	40951.75	C9 H3 O2 S2 Br	285.87523	-0.81	-2.83	8.0

Figure S18: 10 — HREIMS.

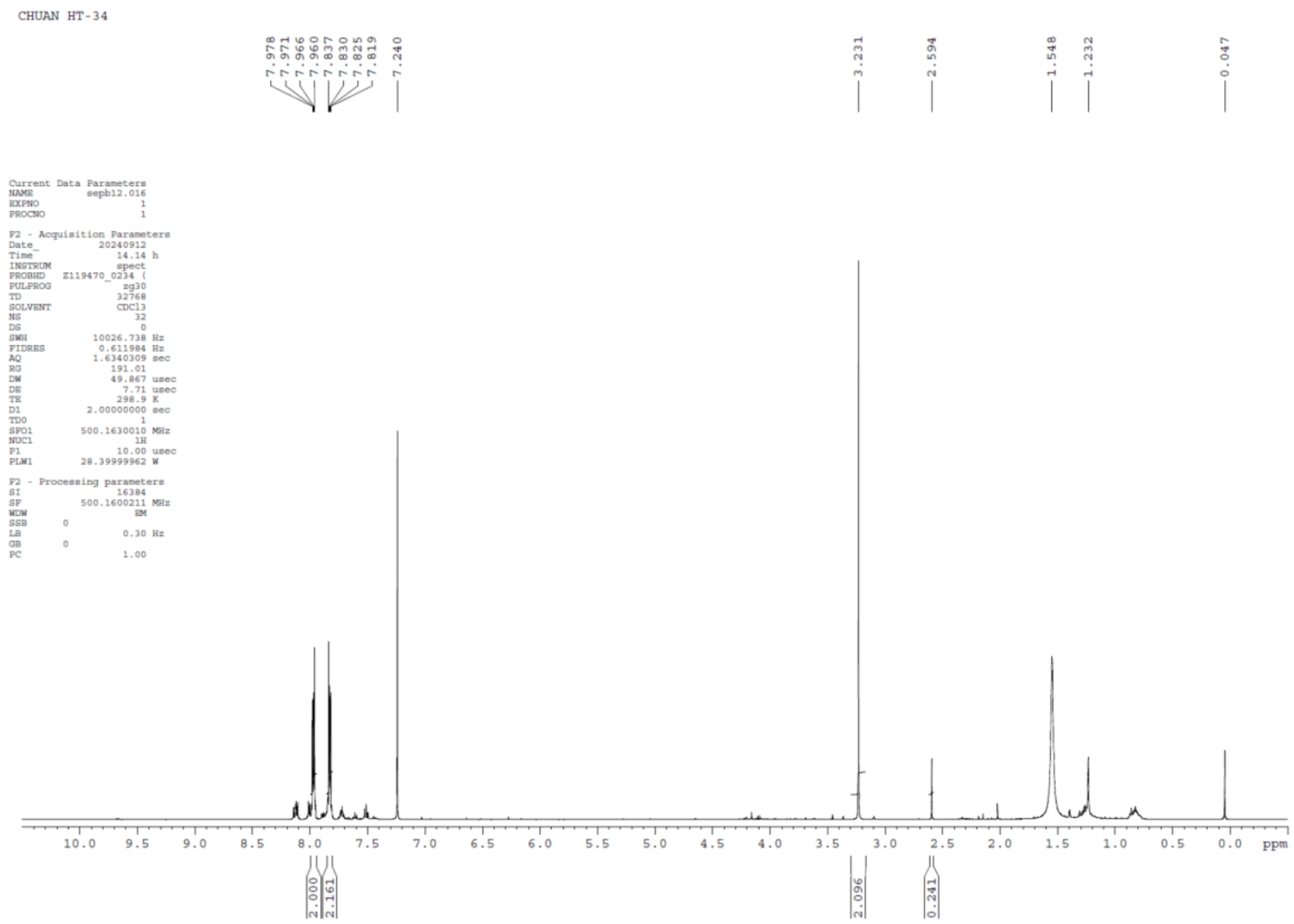

Figure S19: Indan-1,3-dione — ^1H NMR (500 MHz, $CDCl_3$).

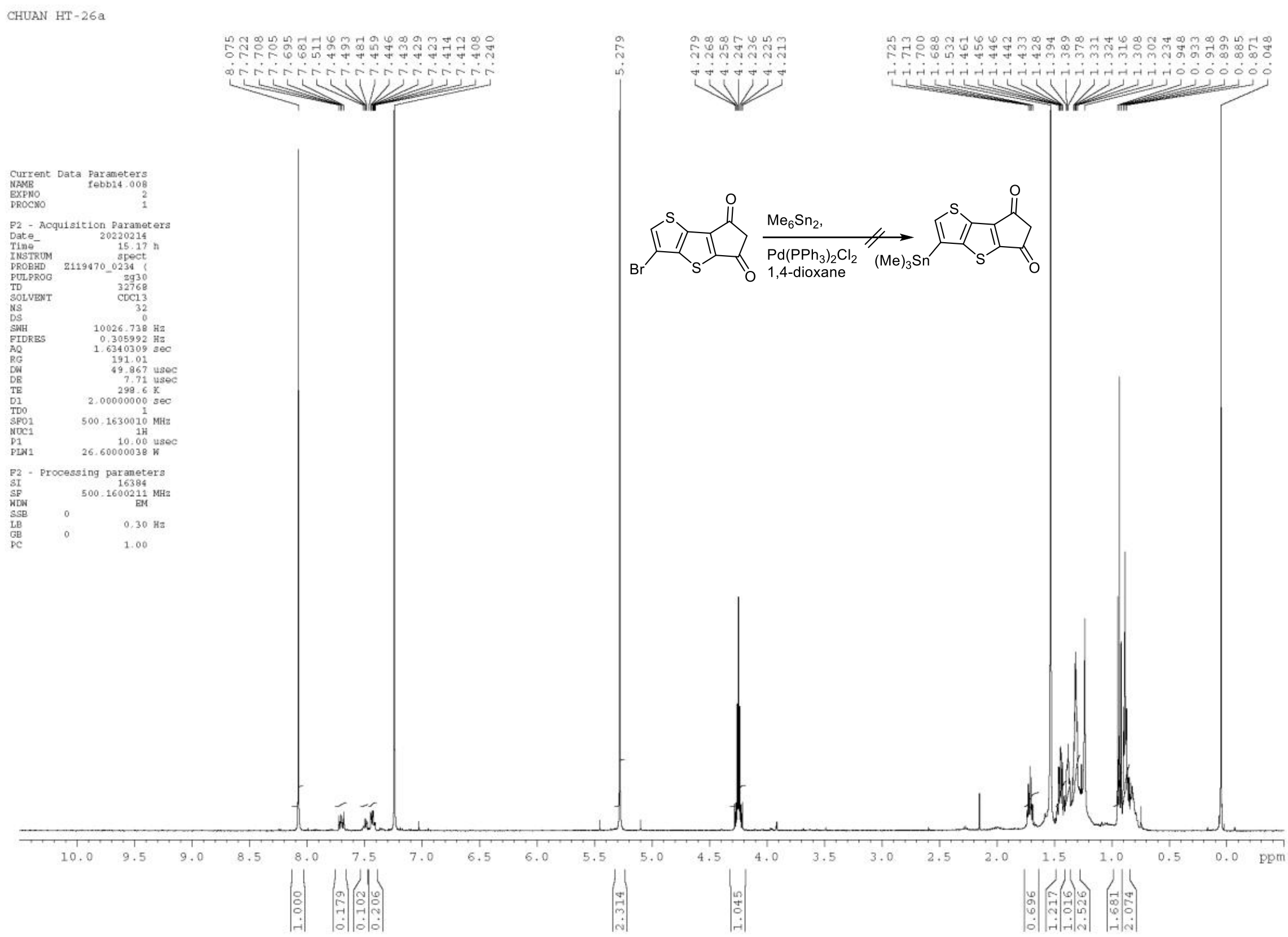

Figure S20: 11 (attempted) — ^{1}H NMR of the crude product from Me_6Sn_2/$Pd(PPh_3)_2Cl_2$ in 1,4-dioxane.

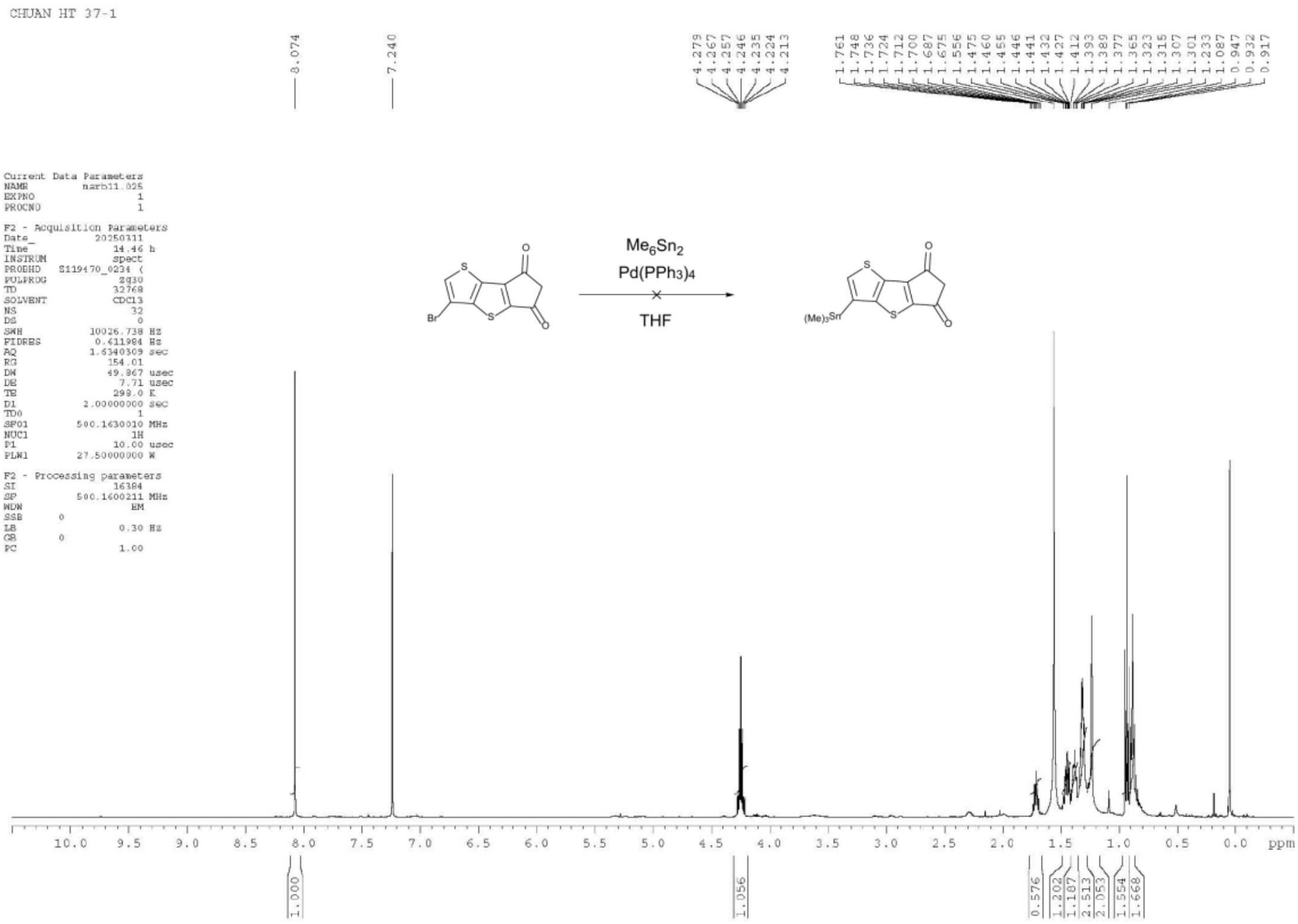

Figure S21: 11 (attempted) — ^{1}H NMR of the crude product from Me_6Sn_2/$Pd(PPh_3)_4$ in THF.

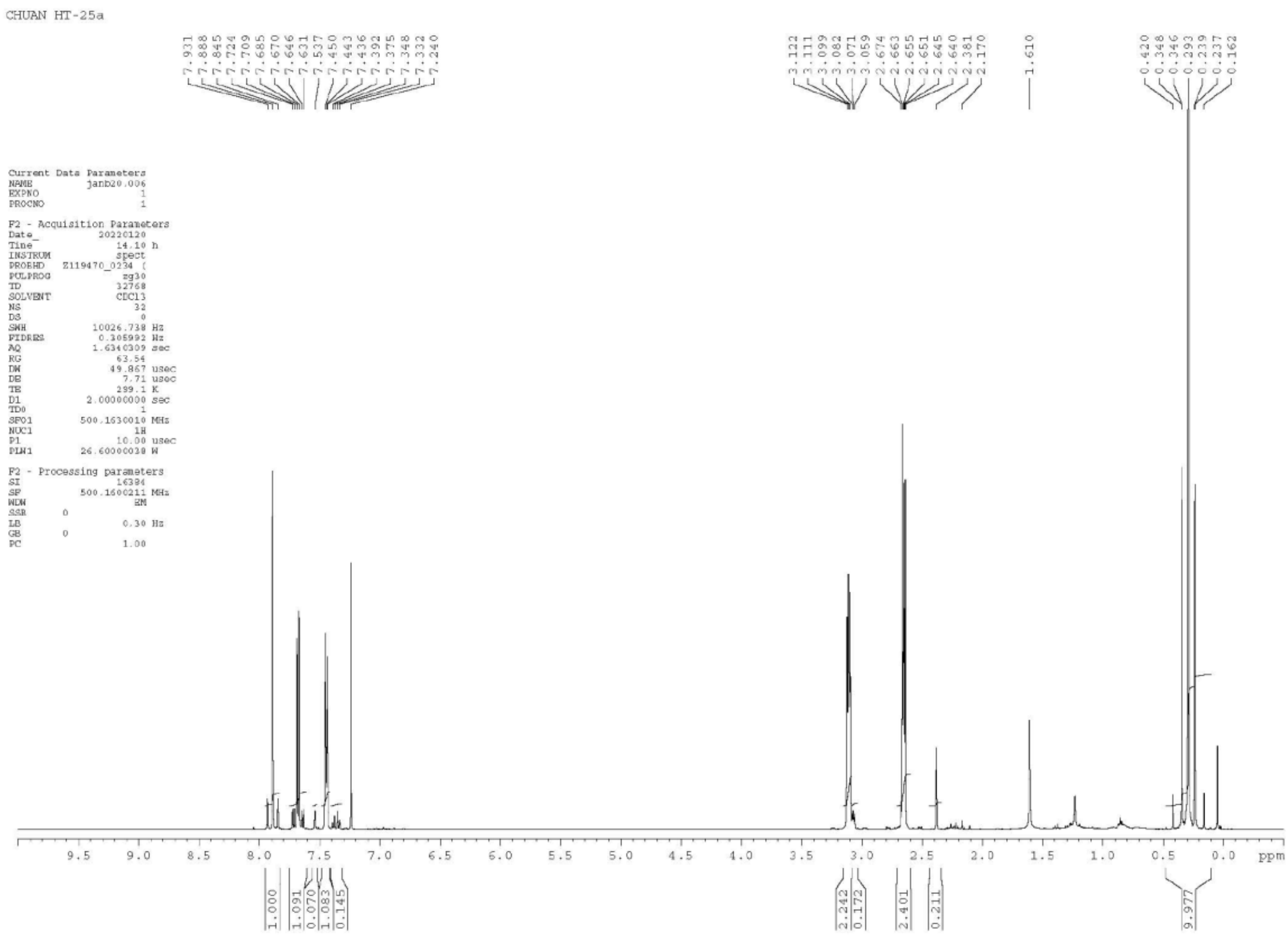

Figure S22: 18 — ^{1}H NMR (500 MHz, $CDCl_3$).

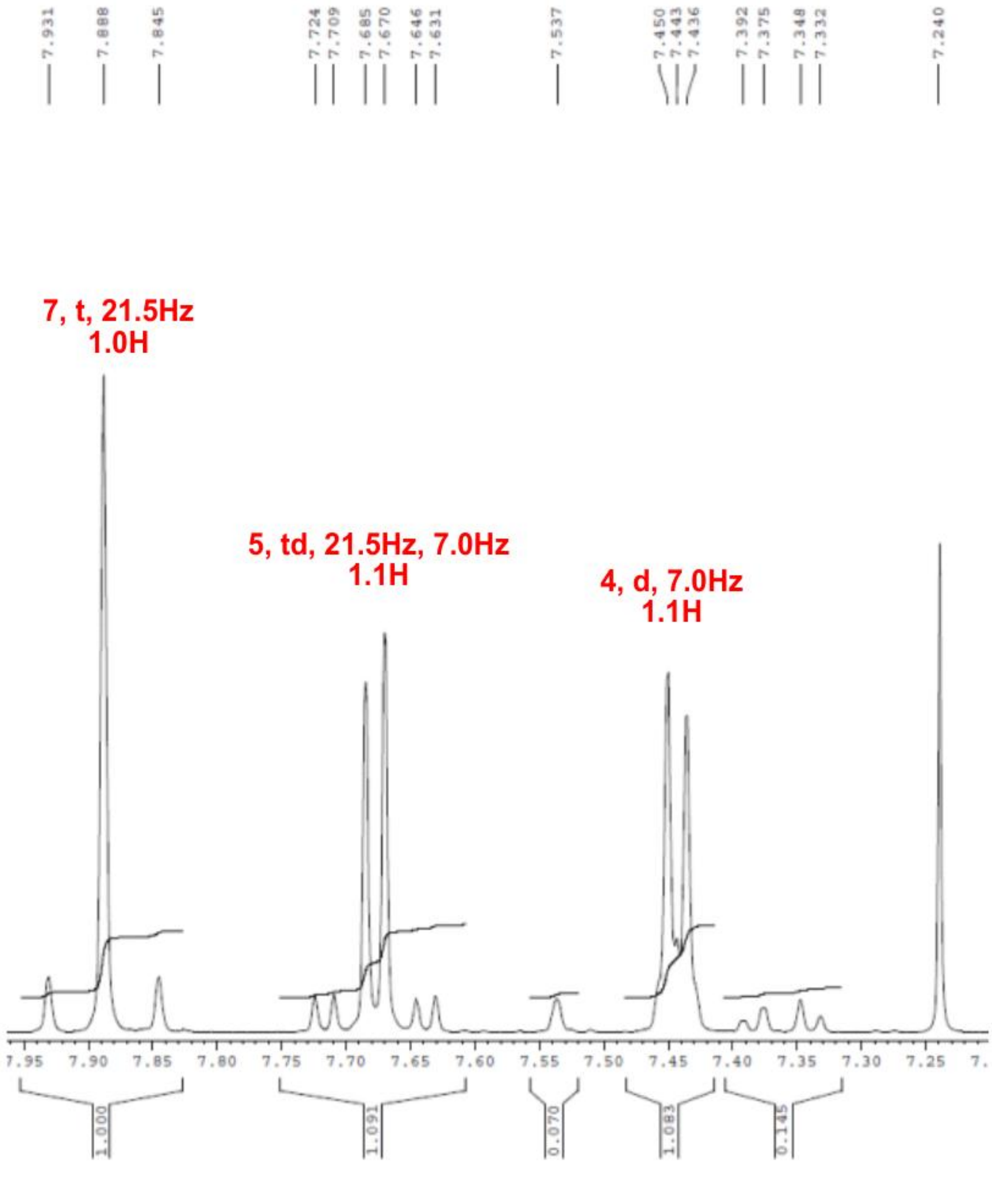

Figure S23: 18 — ^{1}H NMR, aromatic region expanded.

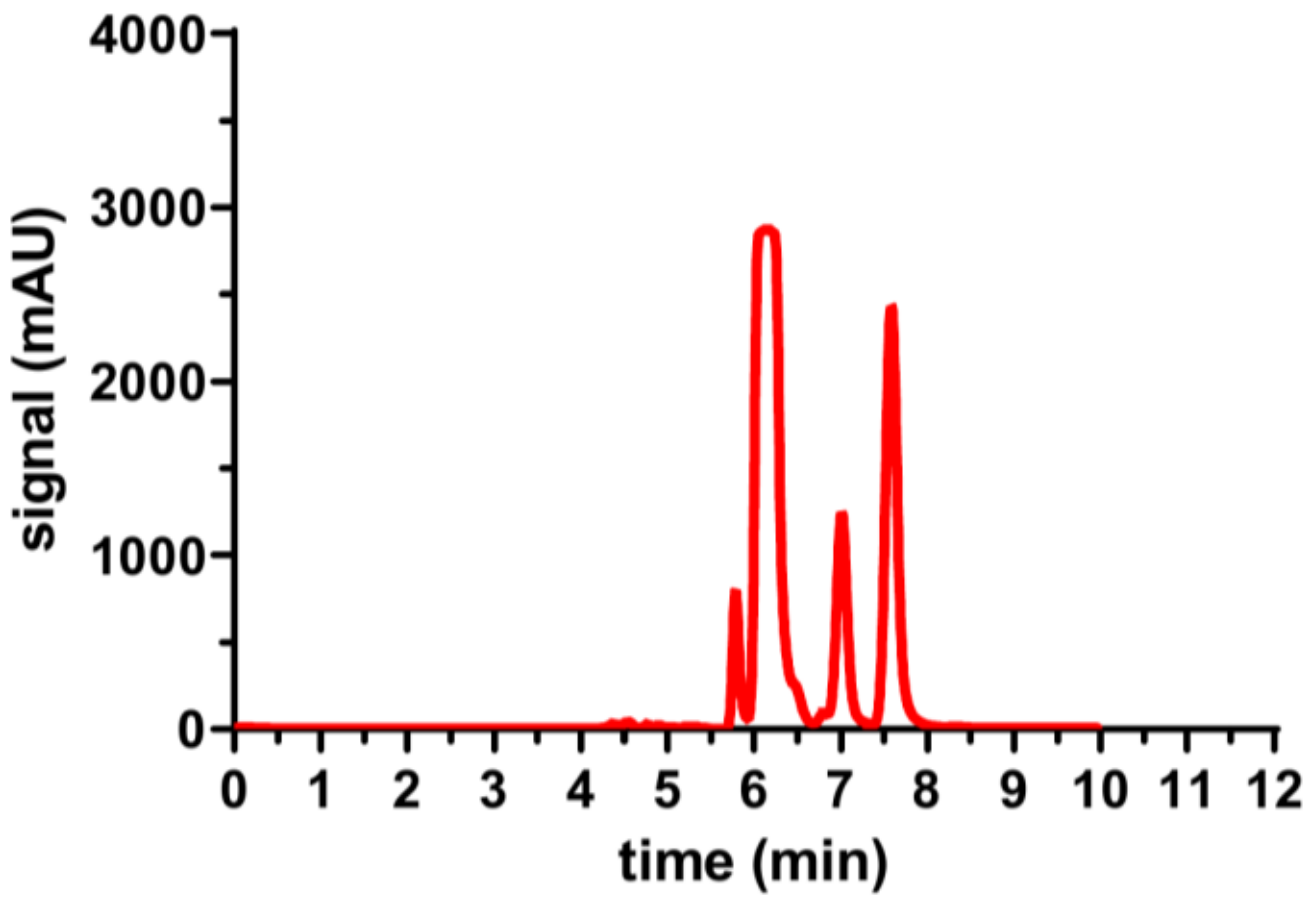

Figure S24: 13 — normal-phase HPLC trace (EtOAc/hexanes 1:19, 254 nm).

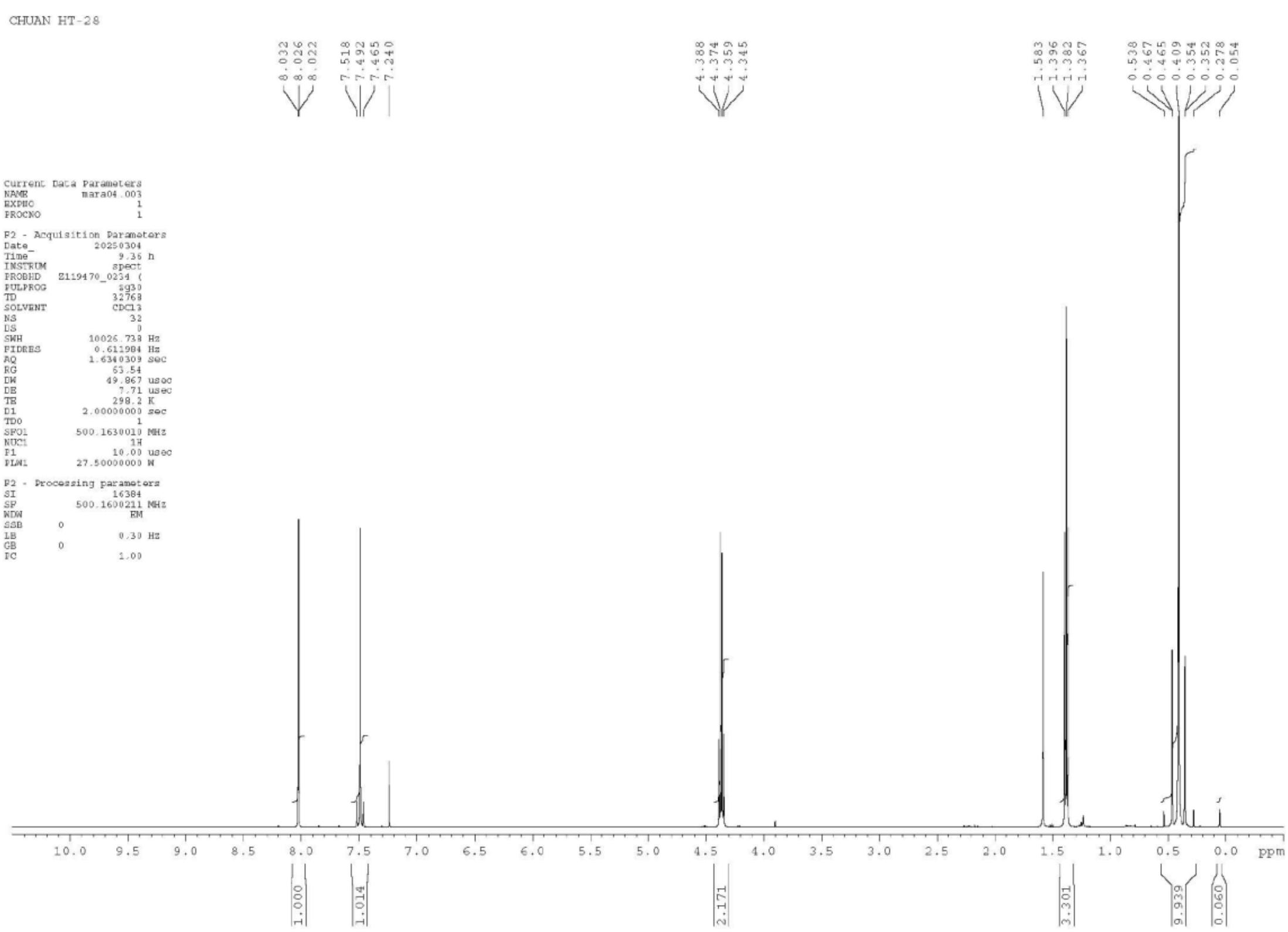

Figure S25: 13 — ^{1}H NMR (500 MHz, $CDCl_3$), HPLC-purified.

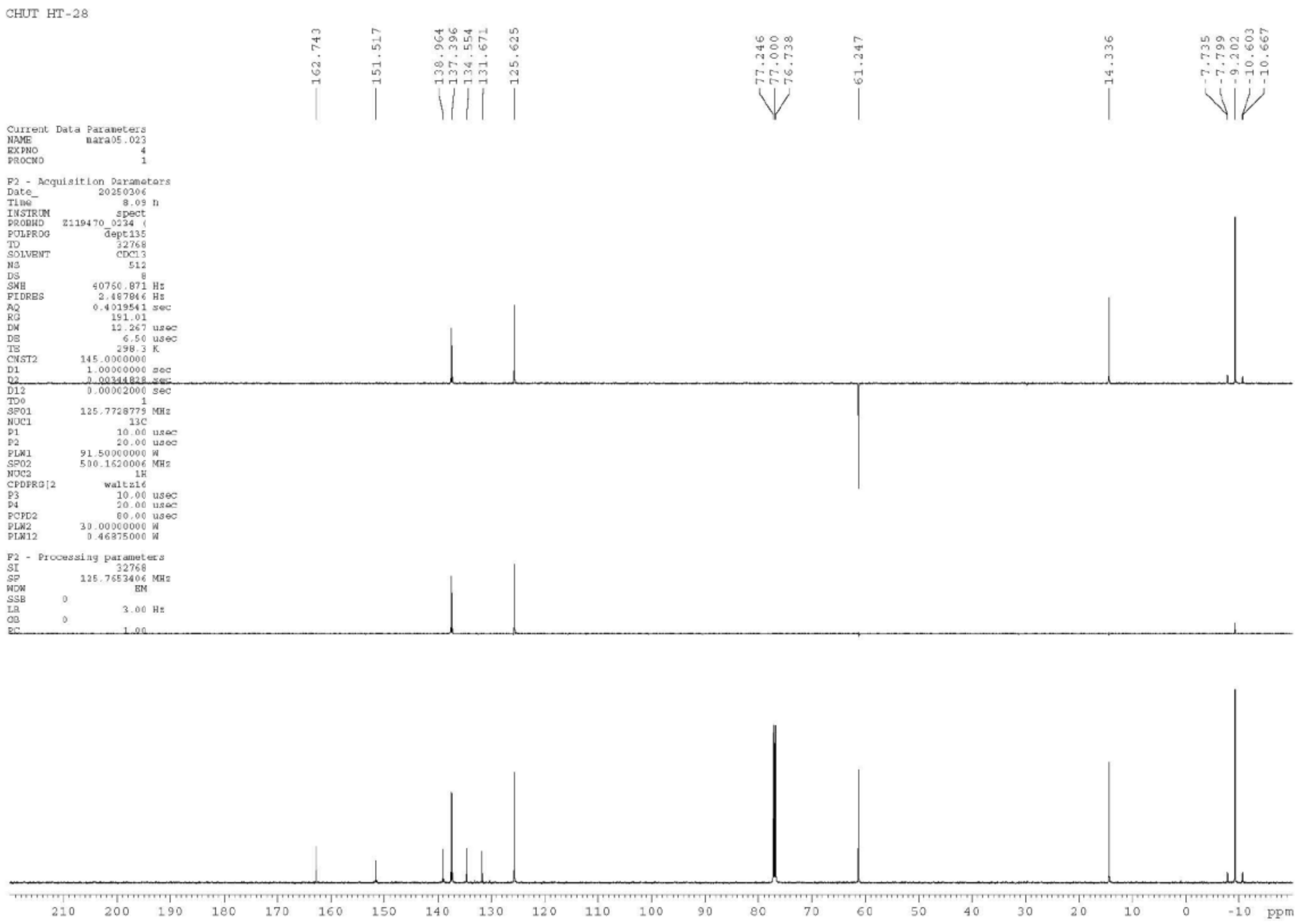

Figure S26: 13 — ^{13}C NMR (125 MHz, $CDCl_3$), HPLC-purified.

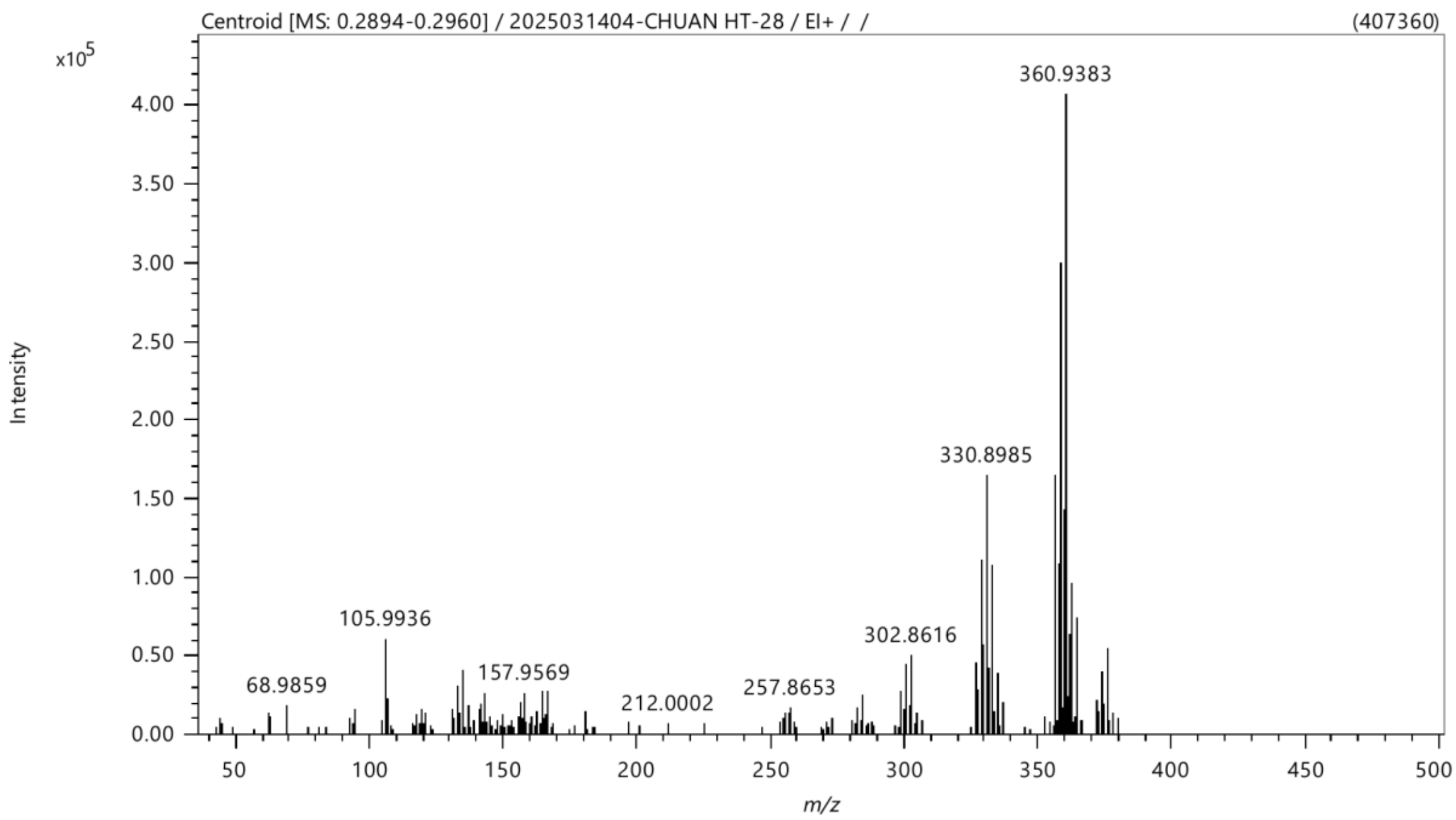

Figure S27: 13 — LREIMS.

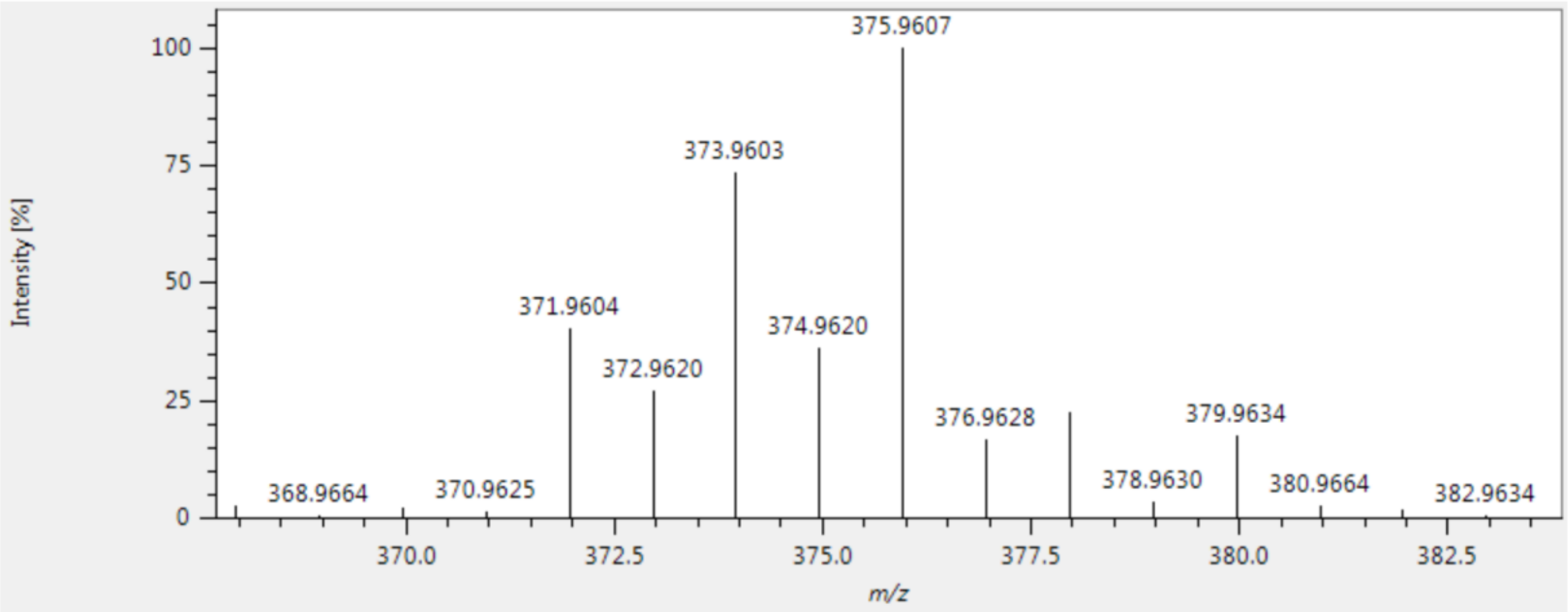

Mass	Intensity	Formula	Calculated Mass	Mass Difference [mDa]	Mass Difference [ppm]	DBE
375.96179	54392.29	C12 H16 O2 S2 Sn	375.96082	0.97	2.58	6.0

Figure S28: 13 — HREIMS, $[M]^+$ region.

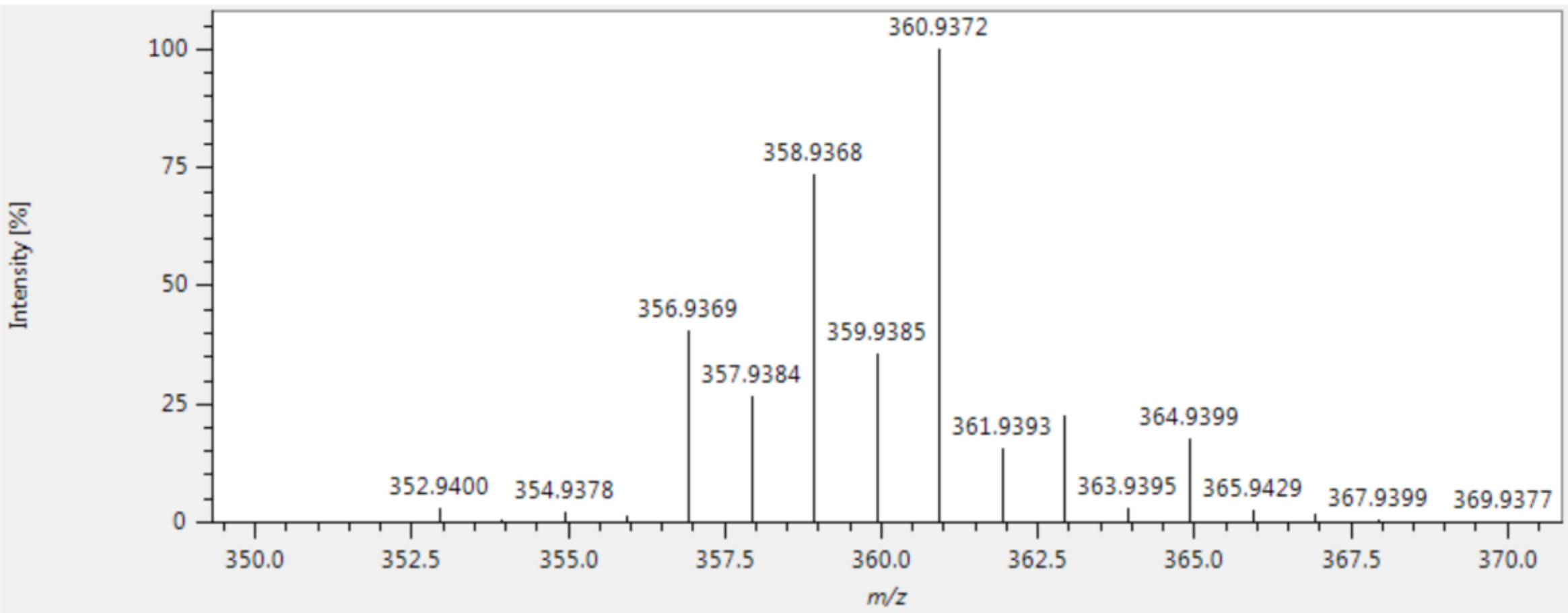

Mass	Intensity	Formula	Calculated Mass	Mass Difference [mDa]	Mass Difference [ppm]	DBE
360.93832	407360.01	C11 H13 O2 S2 Sn	360.93734	0.98	2.71	6.5

Figure S29: 13 — HREIMS, $[M-CH_3]^+$ region.

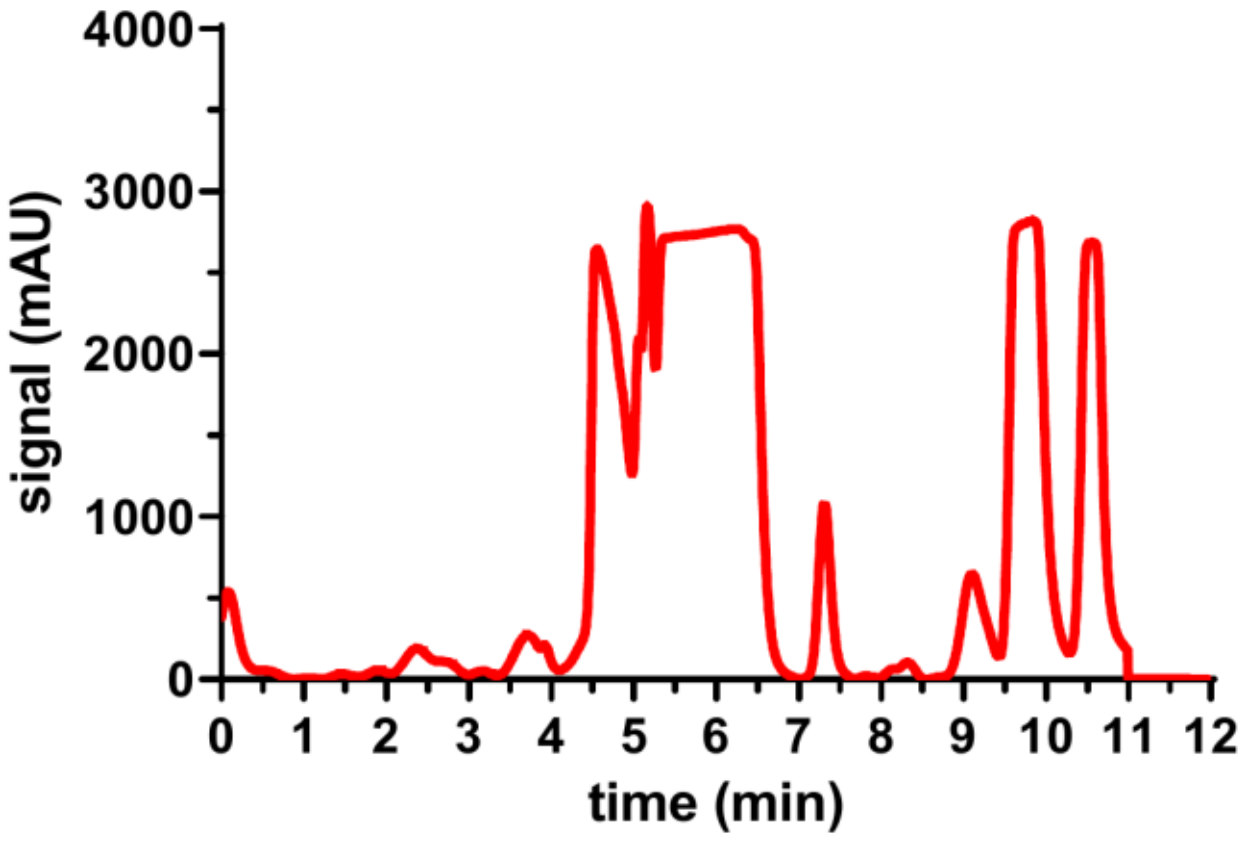

Figure S30: 13′ — normal-phase HPLC trace (EtOAc/hexanes 1:19, 254 nm).

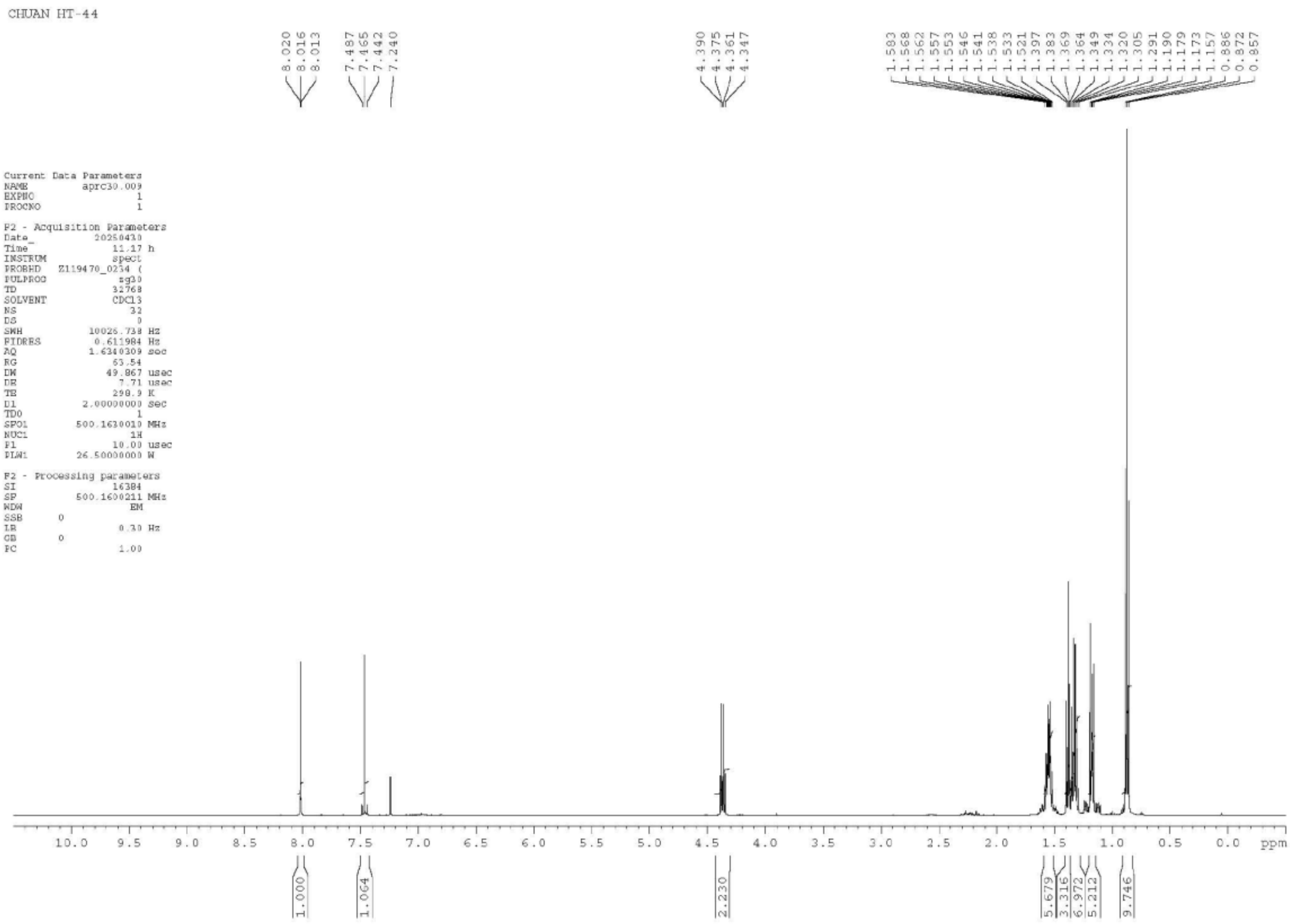

Figure S31: 13′ — ^{1}H NMR (500 MHz, $CDCl_3$), HPLC-purified.

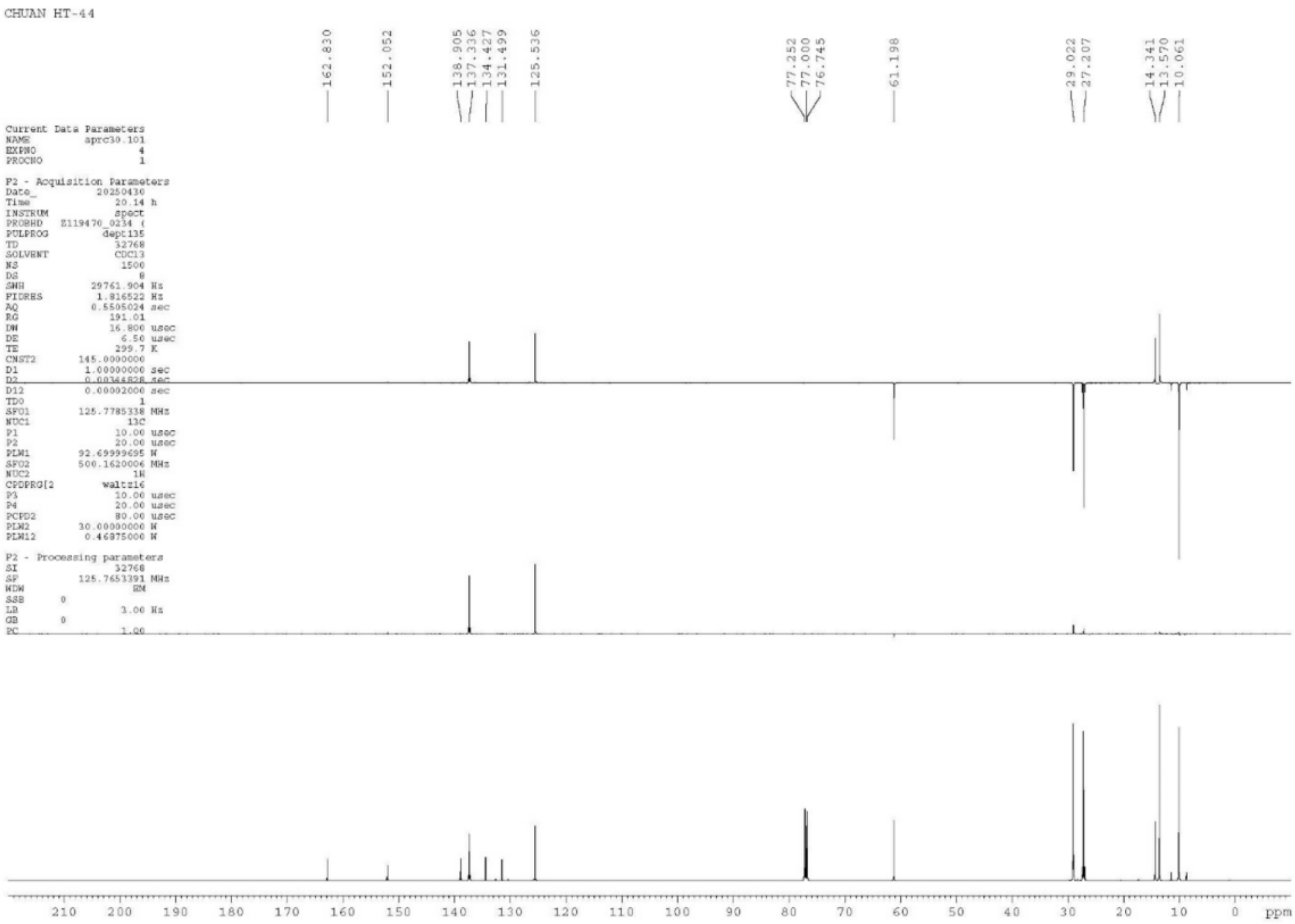

Figure S32: 13′ — ^{13}C NMR (125 MHz, $CDCl_3$), HPLC-purified.

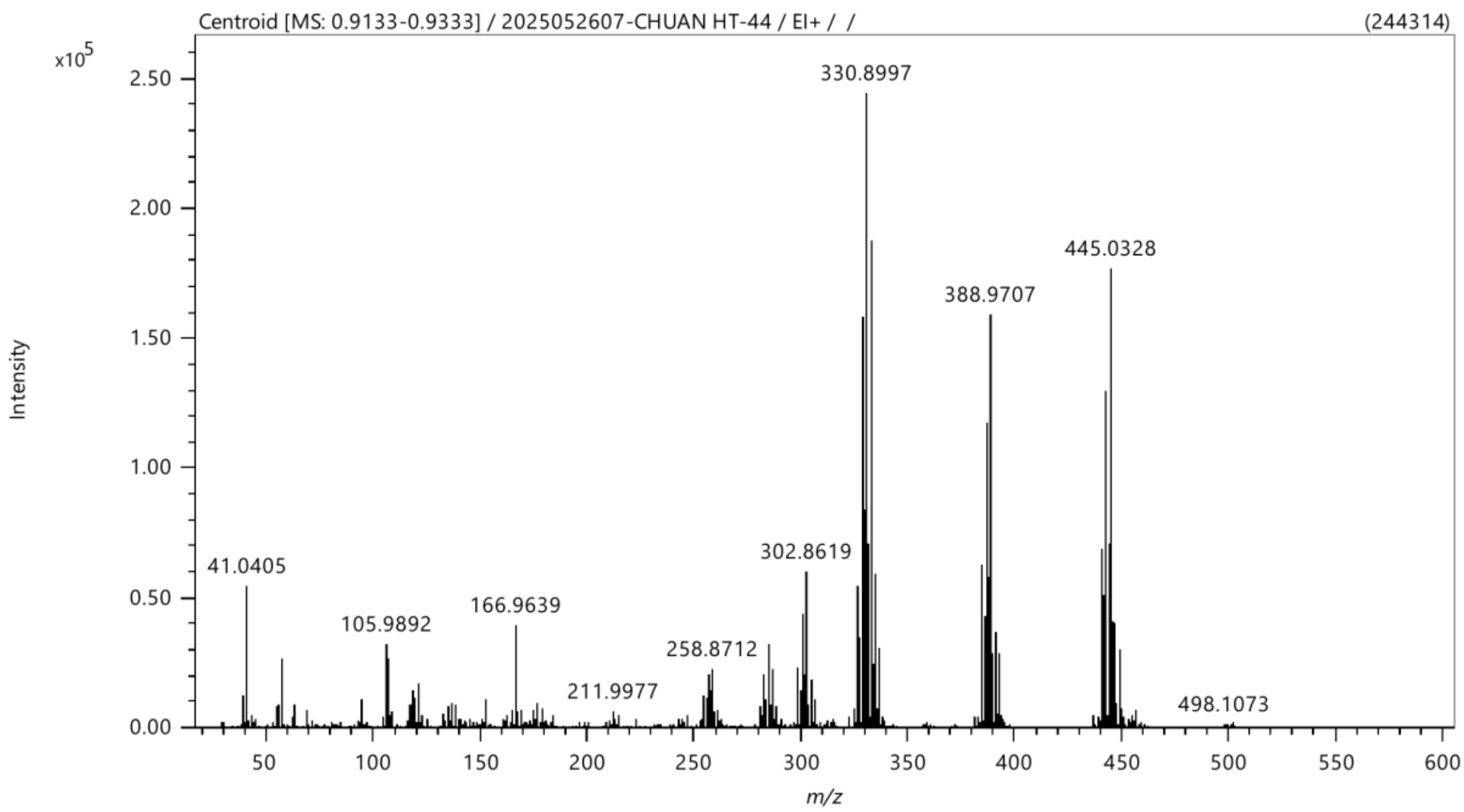

Figure S33: 13′ — LREIMS.

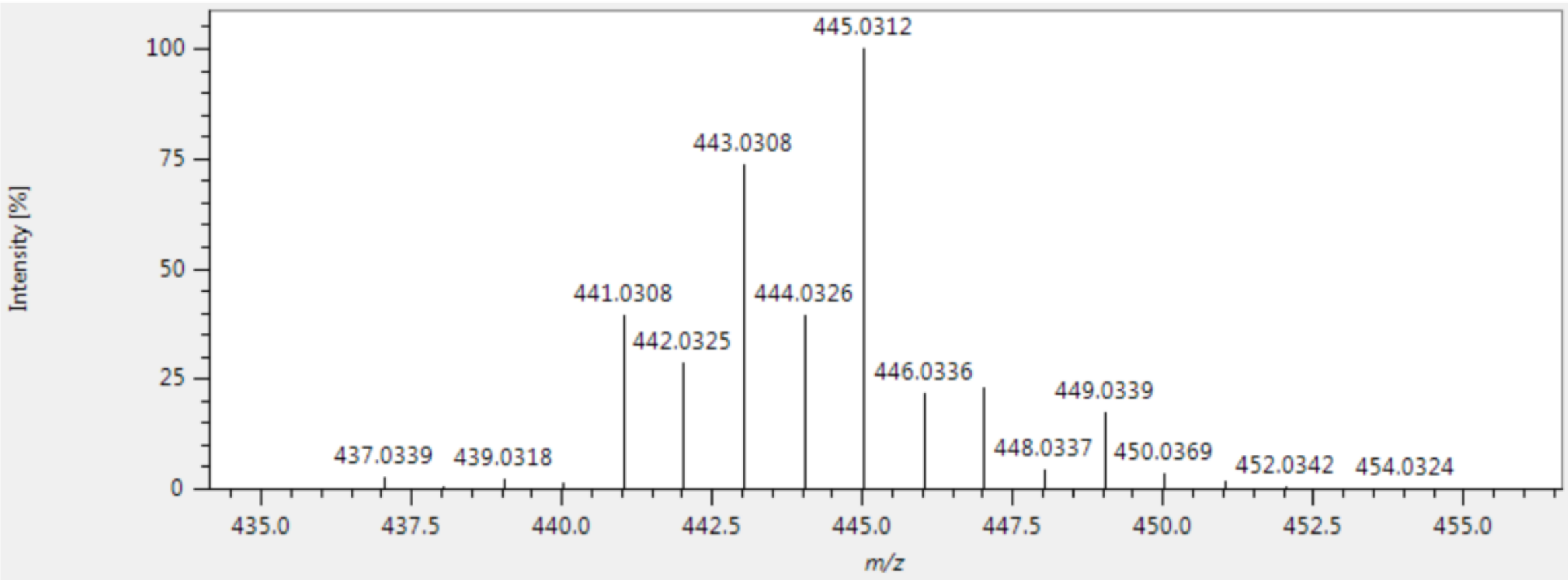

	Mass	Intensity	Formula	Calculated Mass	Mass Difference [mDa]	Mass Difference [ppm]	DBE
▸	445.03280	177029.94	C17 H25 O2 S2 Sn	445.03124	1.55	3.49	6.5

Figure S34: 13′ — HREIMS, $[M]^+$ region.

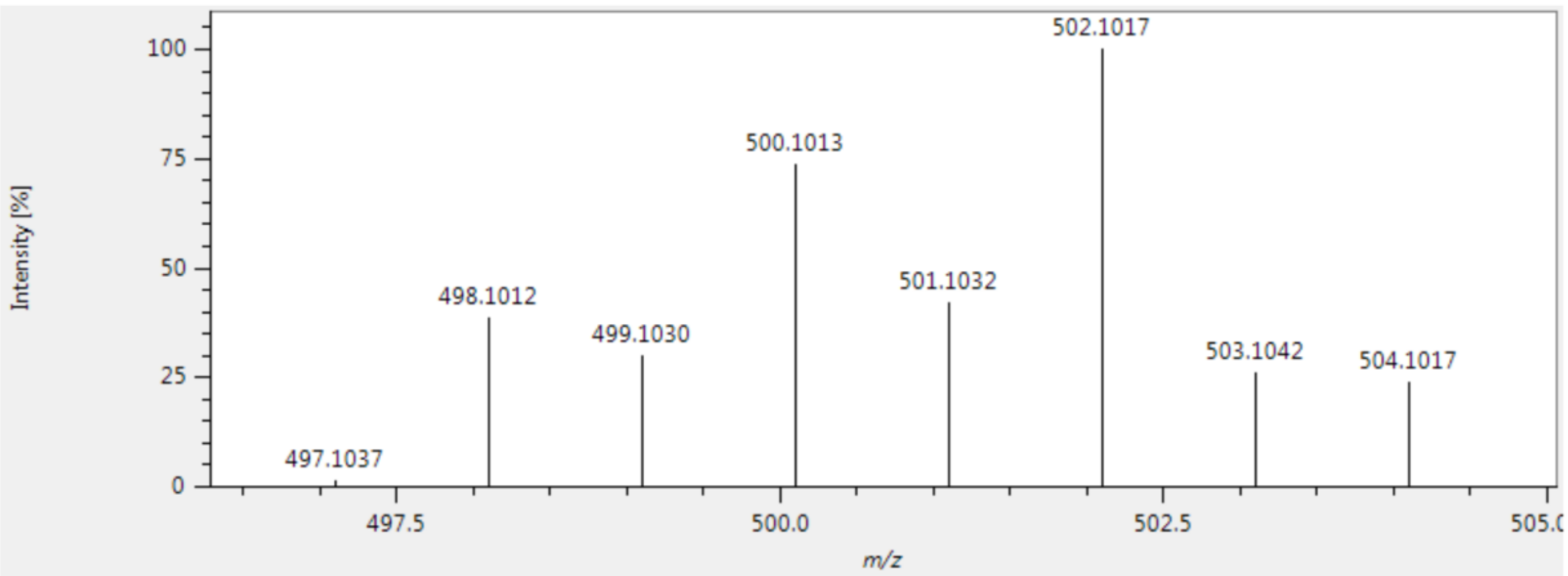

	Mass	Intensity	Formula	Calculated Mass	Mass Difference [mDa]	Mass Difference [ppm]	DBE
▸	502.10153	1841.02	C21 H34 O2 S2 Sn	502.10167	-0.14	-0.28	6.0

Figure S35: 13′ — HREIMS, $[M - C_4H_9]^+$ region.

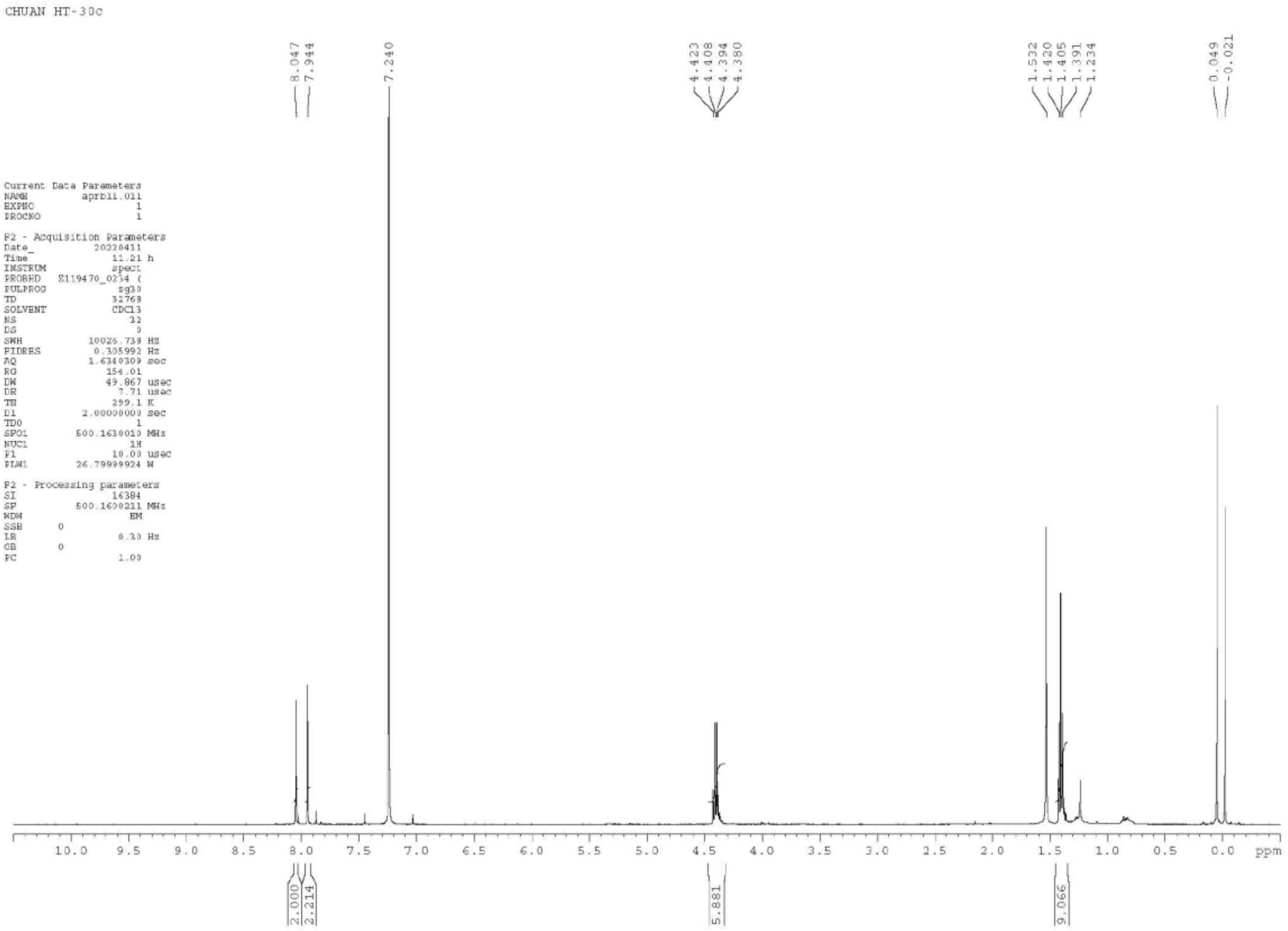

Figure S36: 14 — ^{1}H NMR (500 MHz, $CDCl_3$).

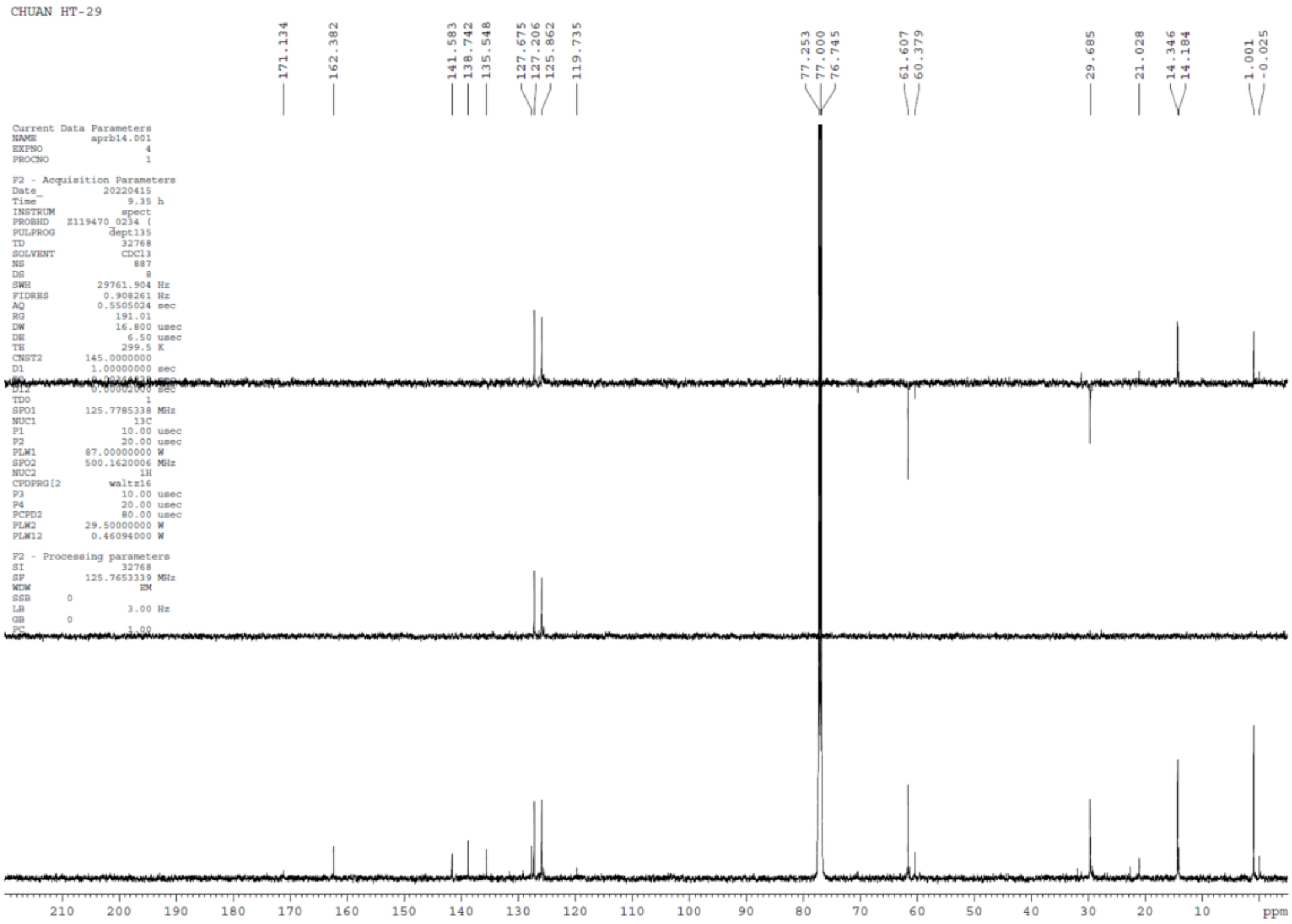

Figure S37: 14 — ^{13}C NMR (125 MHz, $CDCl_3$).

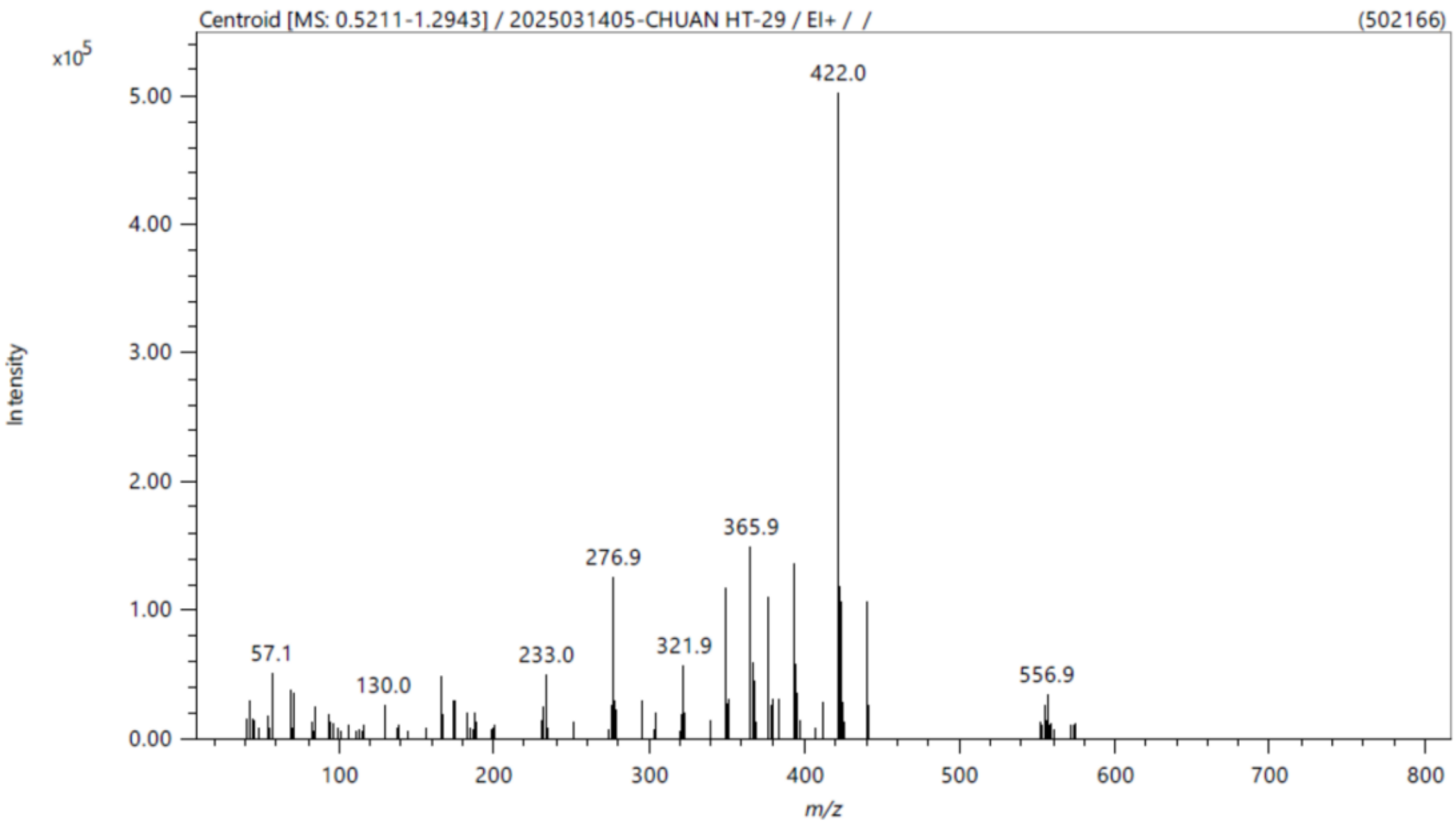

Figure S38: 14 — LREIMS.

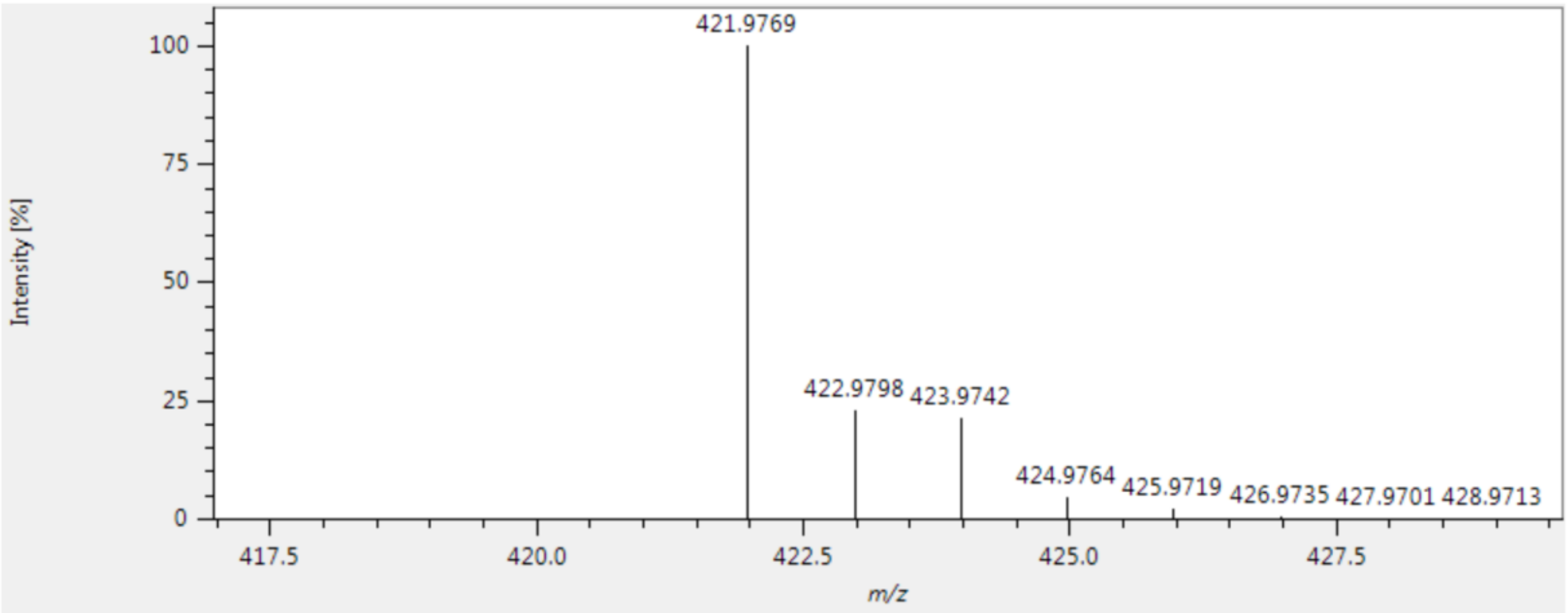

	Mass	Intensity	Formula	Calculated Mass	Mass Difference [mDa]	Mass Difference [ppm]	DBE
▶	421.97778	764562.29	C18 H14 O4 S4	421.97694	0.83	1.97	12.0

Figure S39: 14 — HREIMS.

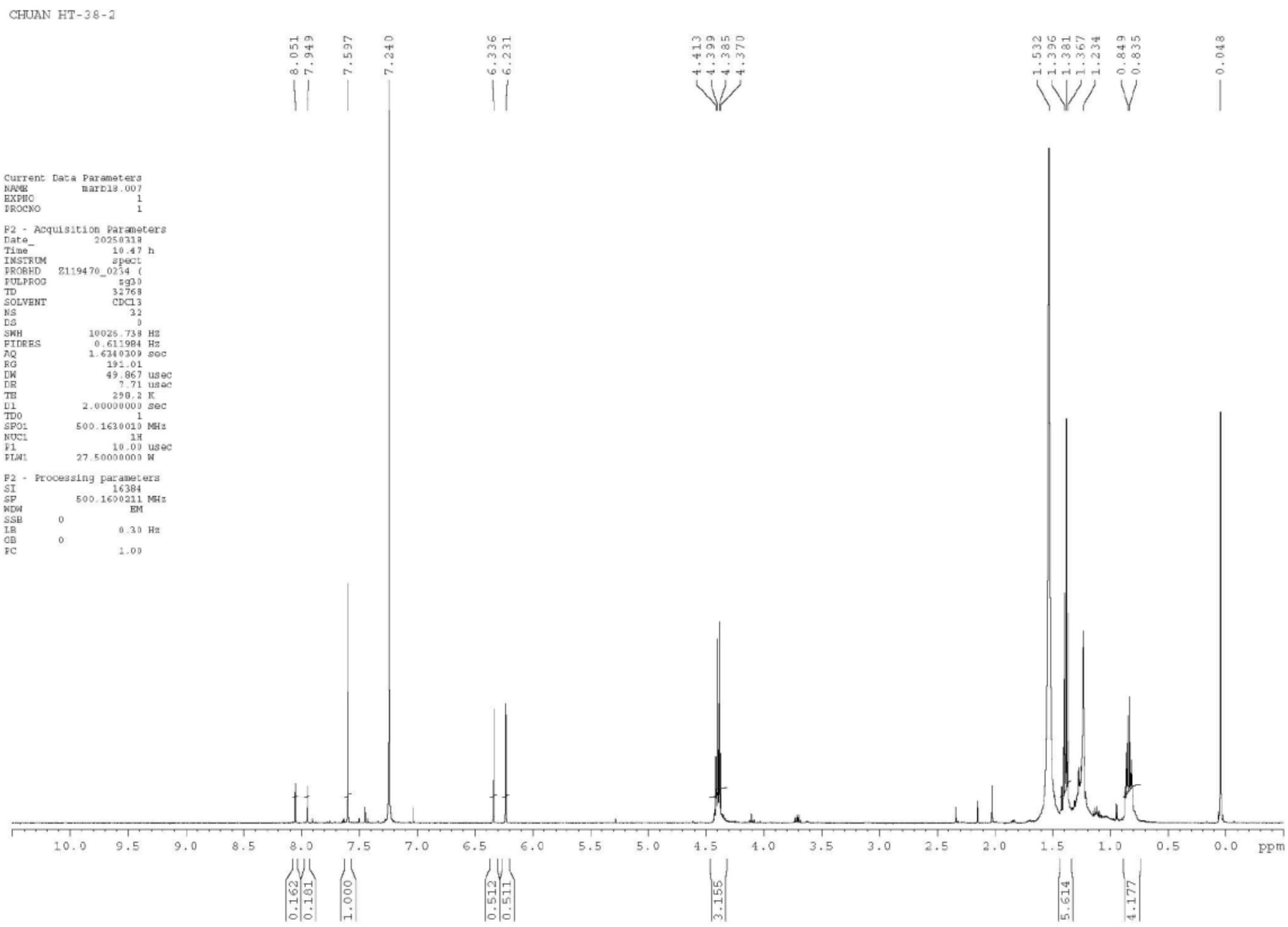

Figure S40: 16 — ^{1}H NMR (500 MHz, $CDCl_3$), from **13**, before HPLC.

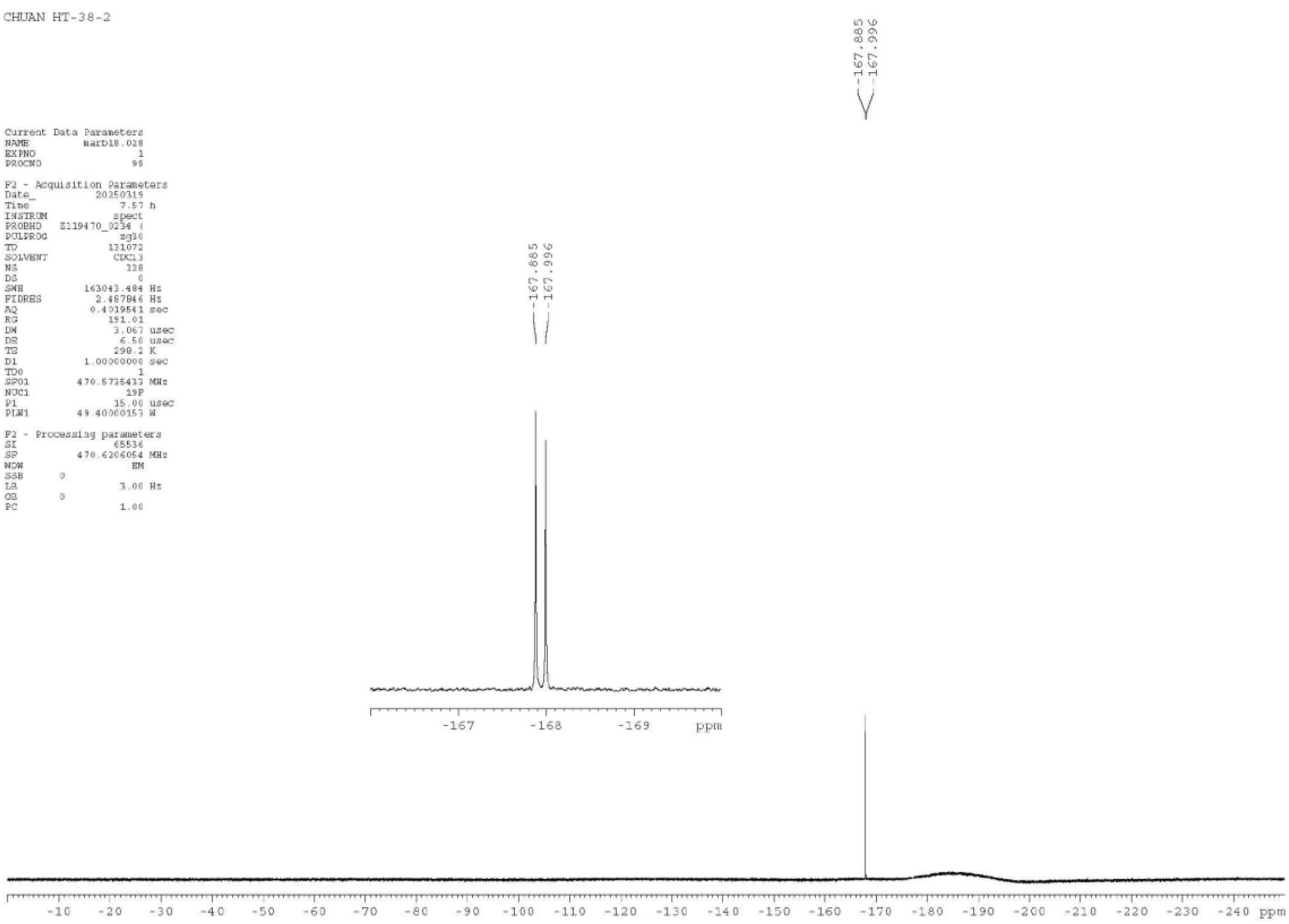

Figure S41: 16 — ^{19}F NMR (470 MHz, $CDCl_3$), from **13**, before HPLC.

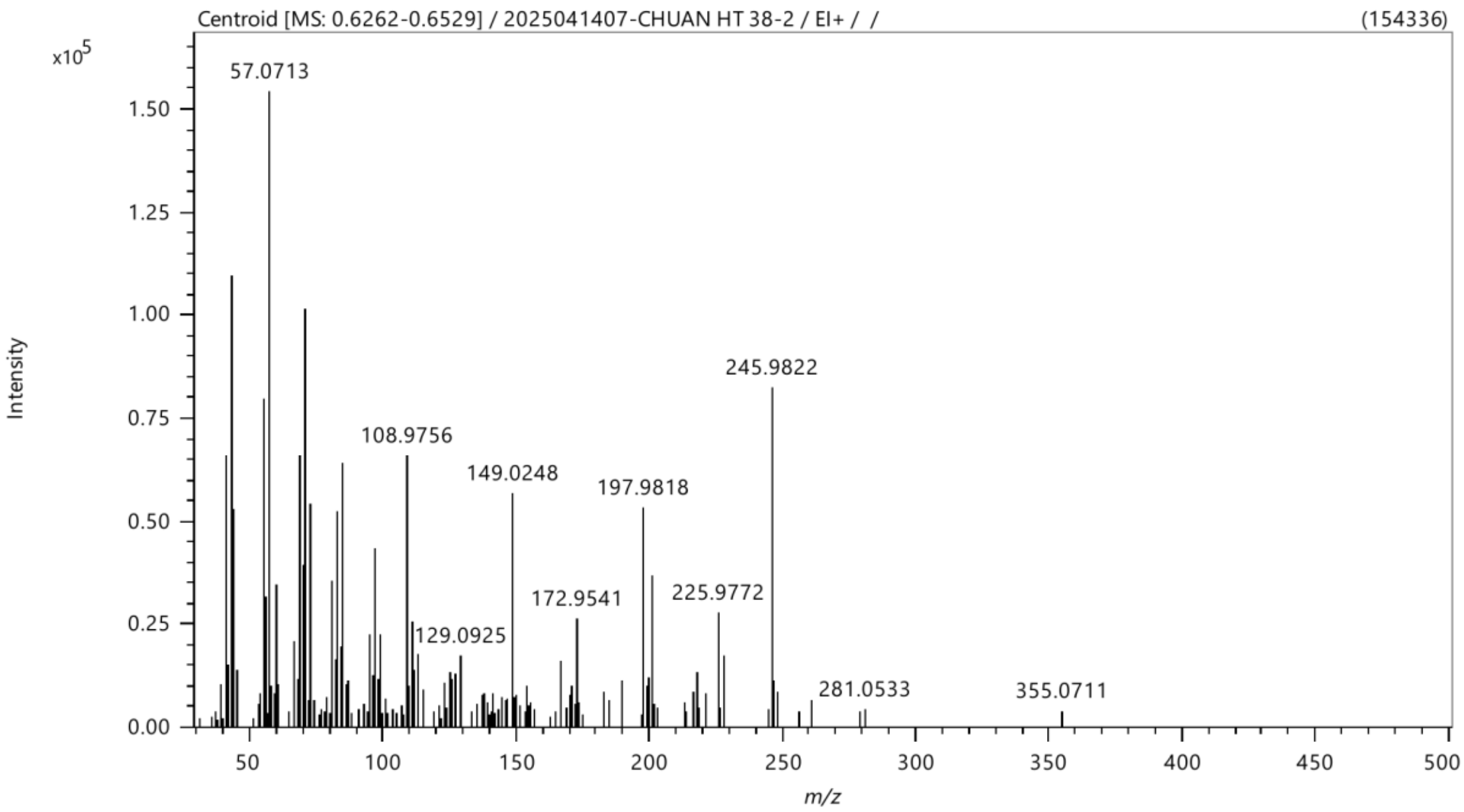

Figure S42: 16 — LREIMS, from **13**.

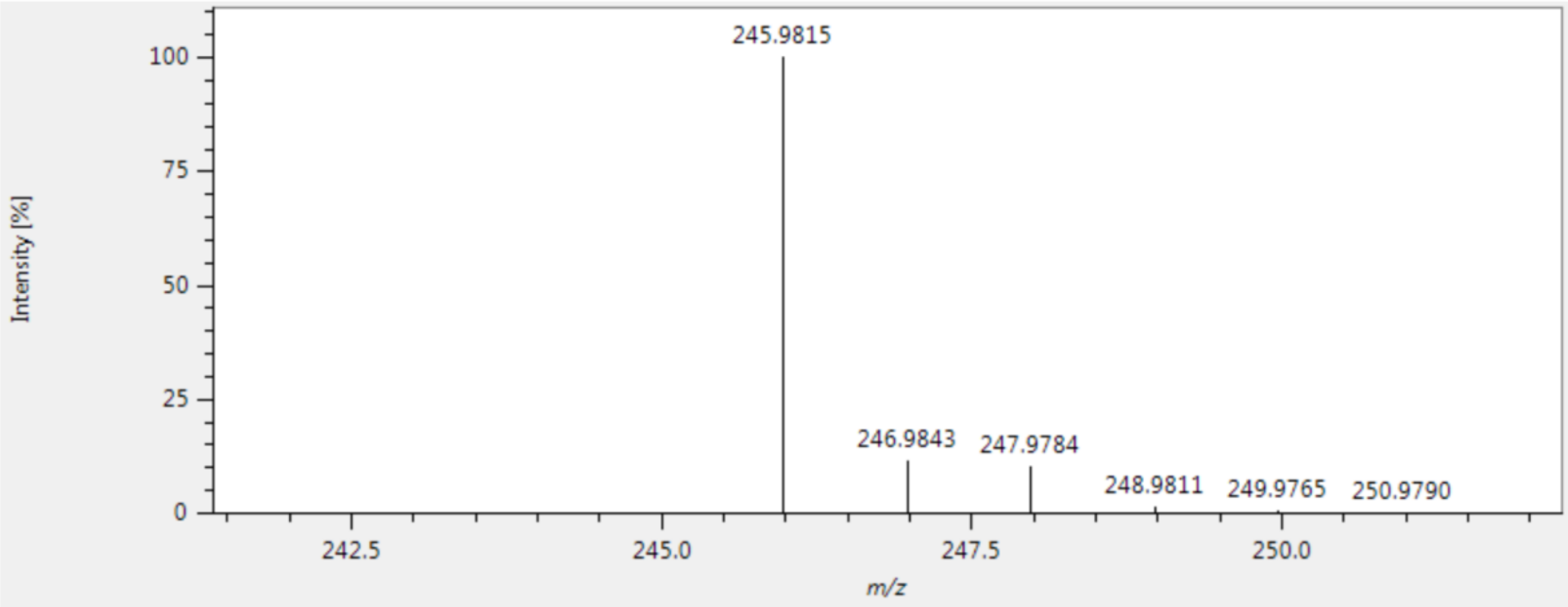

Mass	Intensity	Formula	Calculated Mass	Mass Difference [mDa]	Mass Difference [ppm]	DBE
245.98215	82186.20	C9 H7 O3 F S2	245.98152	0.64	2.59	6.0

Figure S43: 16 — HREIMS, from **13**, showing [M]$^{+}$ and the absence of the aryl fluoride ion.

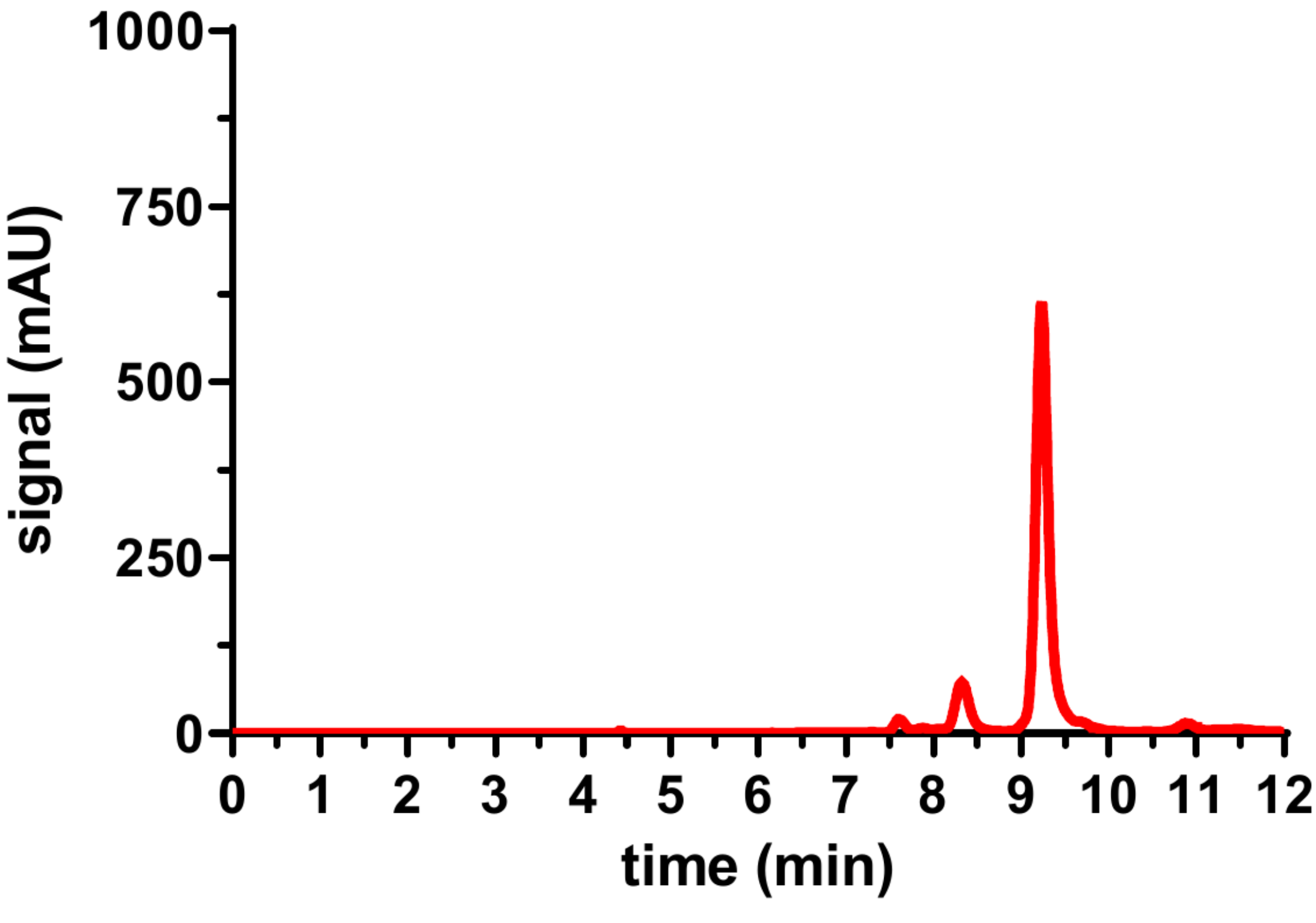

Figure S44: 16 — normal-phase HPLC trace (EtOAc/hexanes 1.5:8.5, 366 nm), from **13′**.

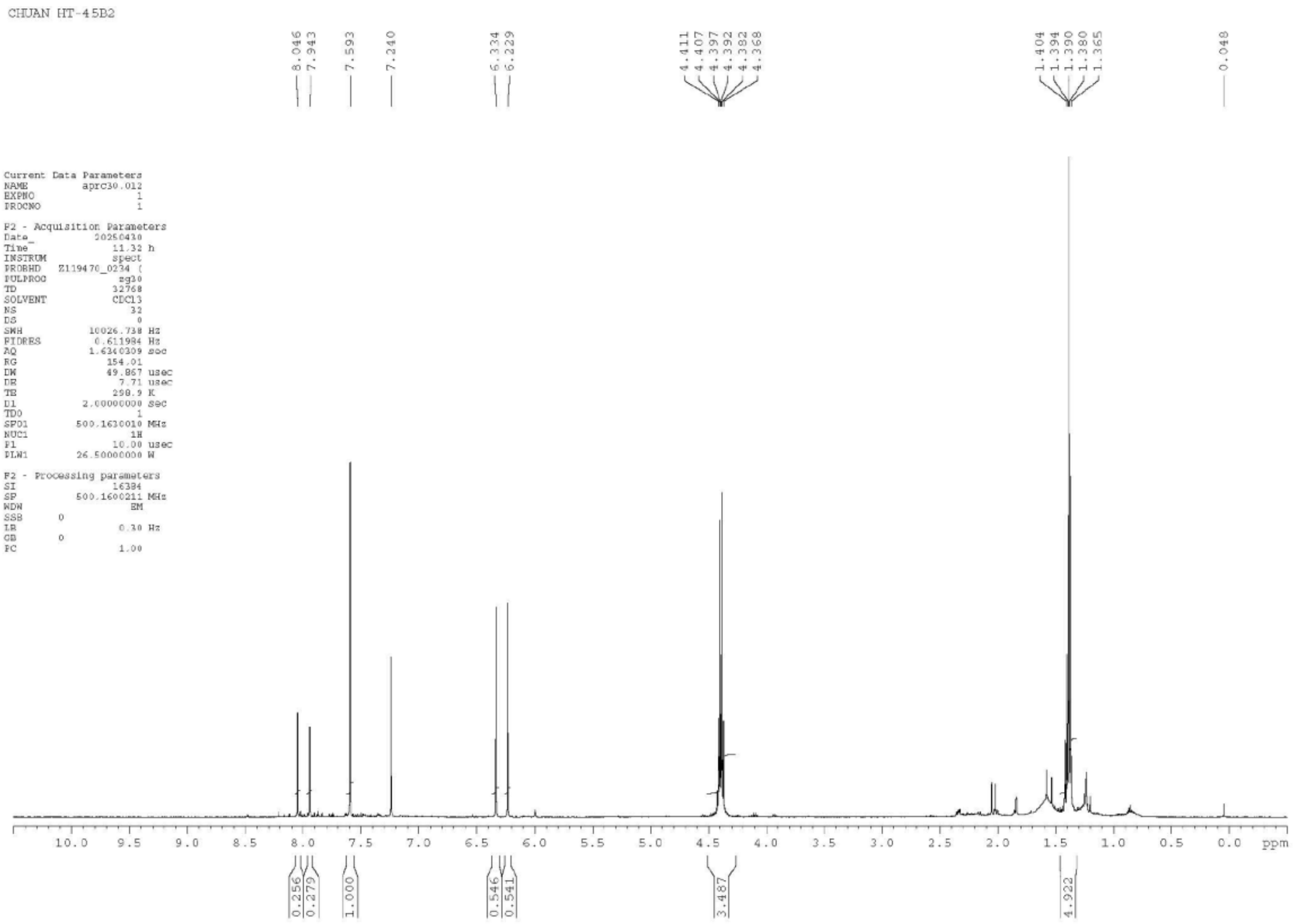

Figure S45: 16 — ^{1}H NMR (500 MHz, $CDCl_3$), HPLC-purified, from **13′**.

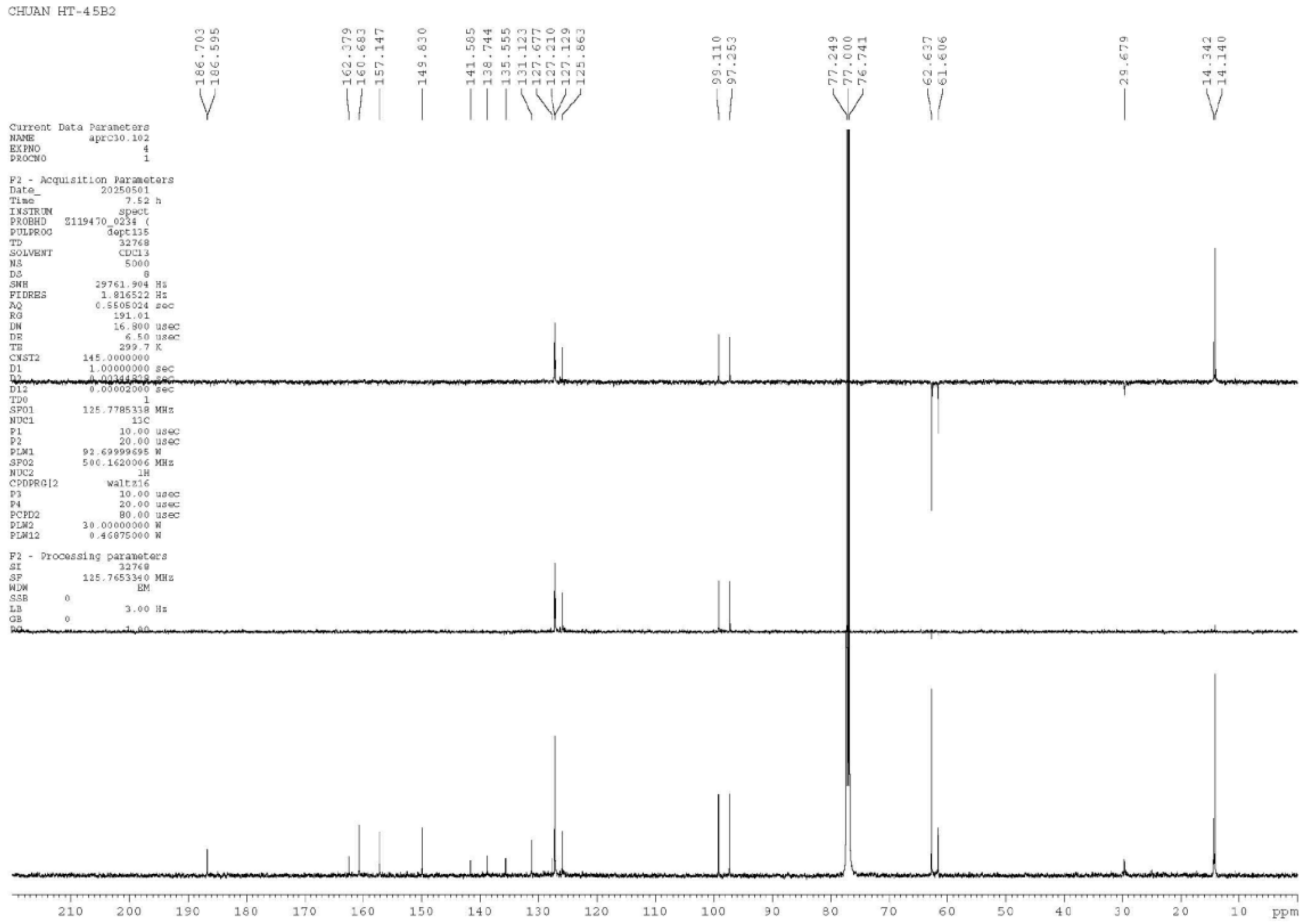

Figure S46: 16 — ^{13}C NMR (125 MHz, $CDCl_3$), HPLC-purified, from **13′**.

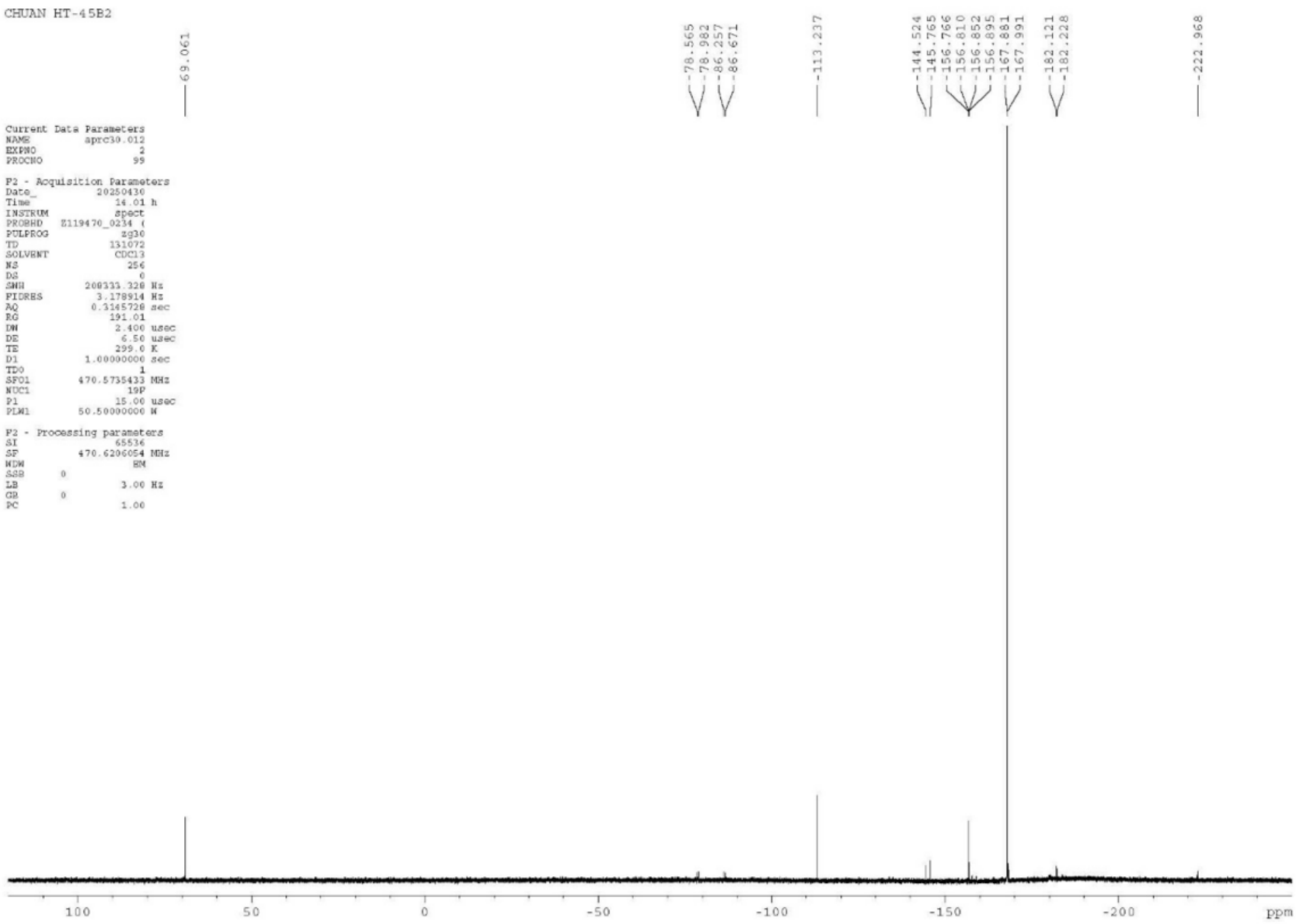

Figure S47: 16 — ^{19}F NMR (470 MHz, $CDCl_3$), HPLC-purified, from **13′**.

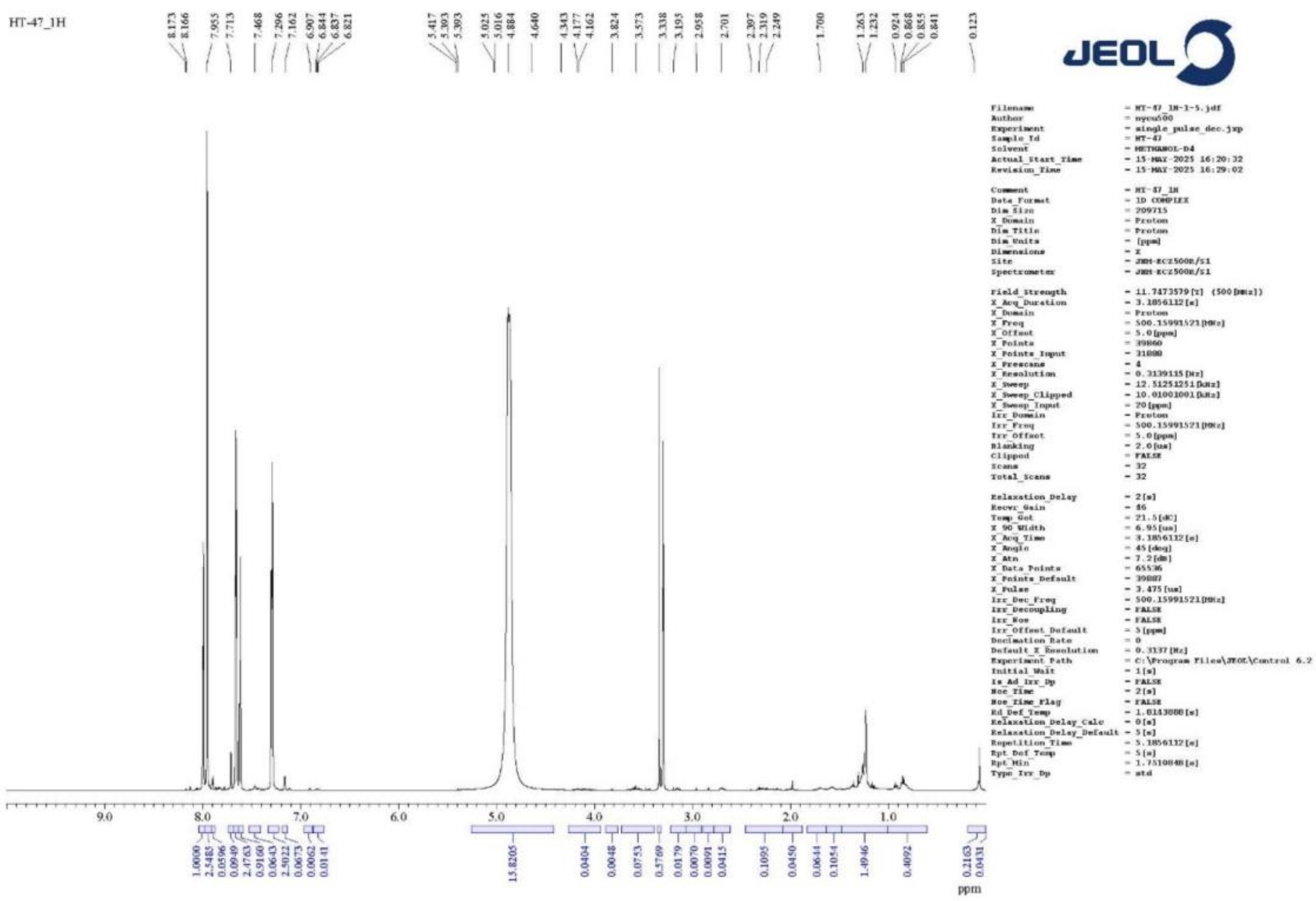

Figure S48: Thieno[3,2-*b*]thiophene-2-carboxylic acid — ^{1}H NMR (500 MHz, $CD_3OD/CDCl_3$ 2:1).

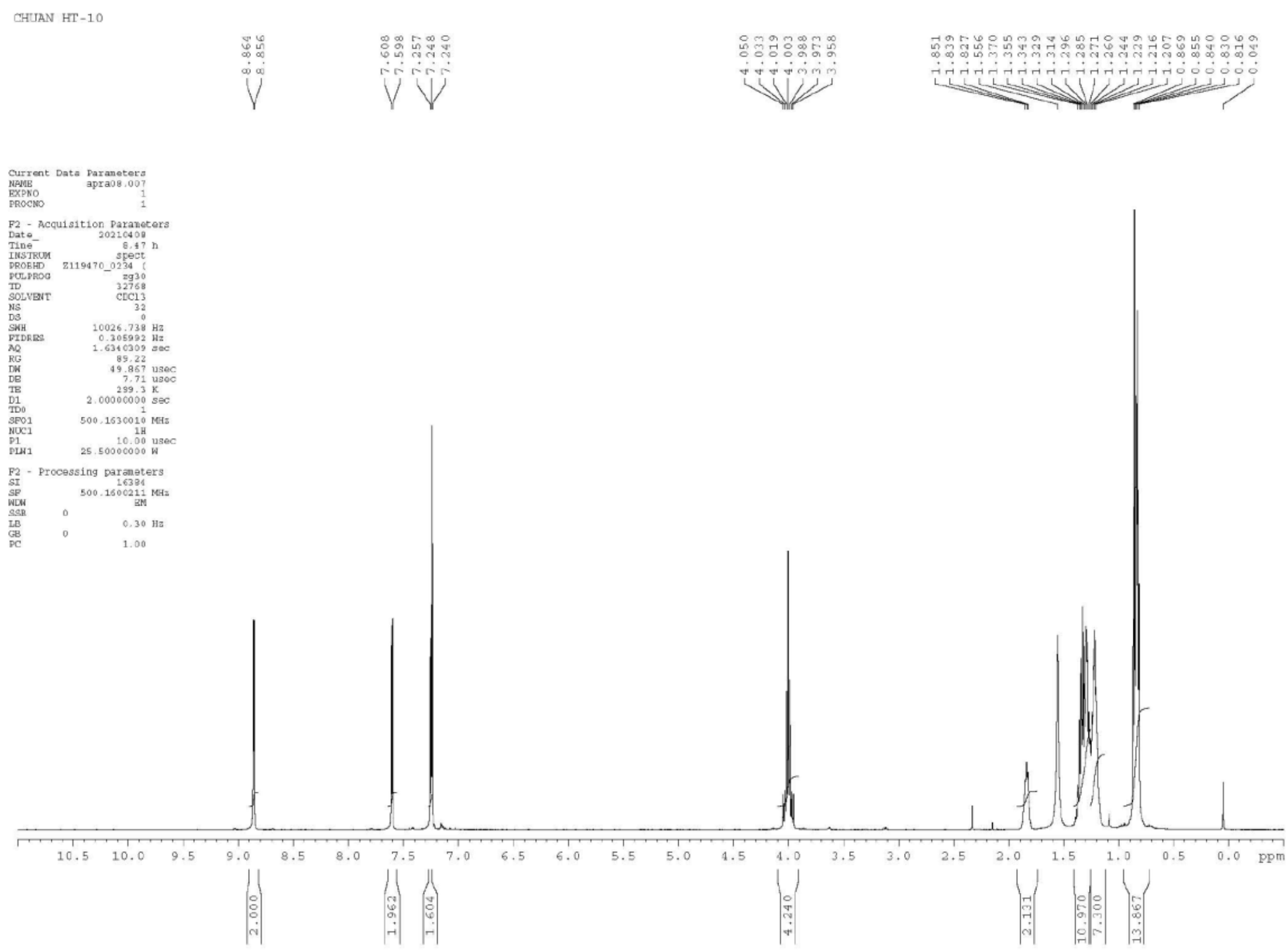

Figure S49: 4 — ^{1}H NMR (500 MHz, $CDCl_3$).

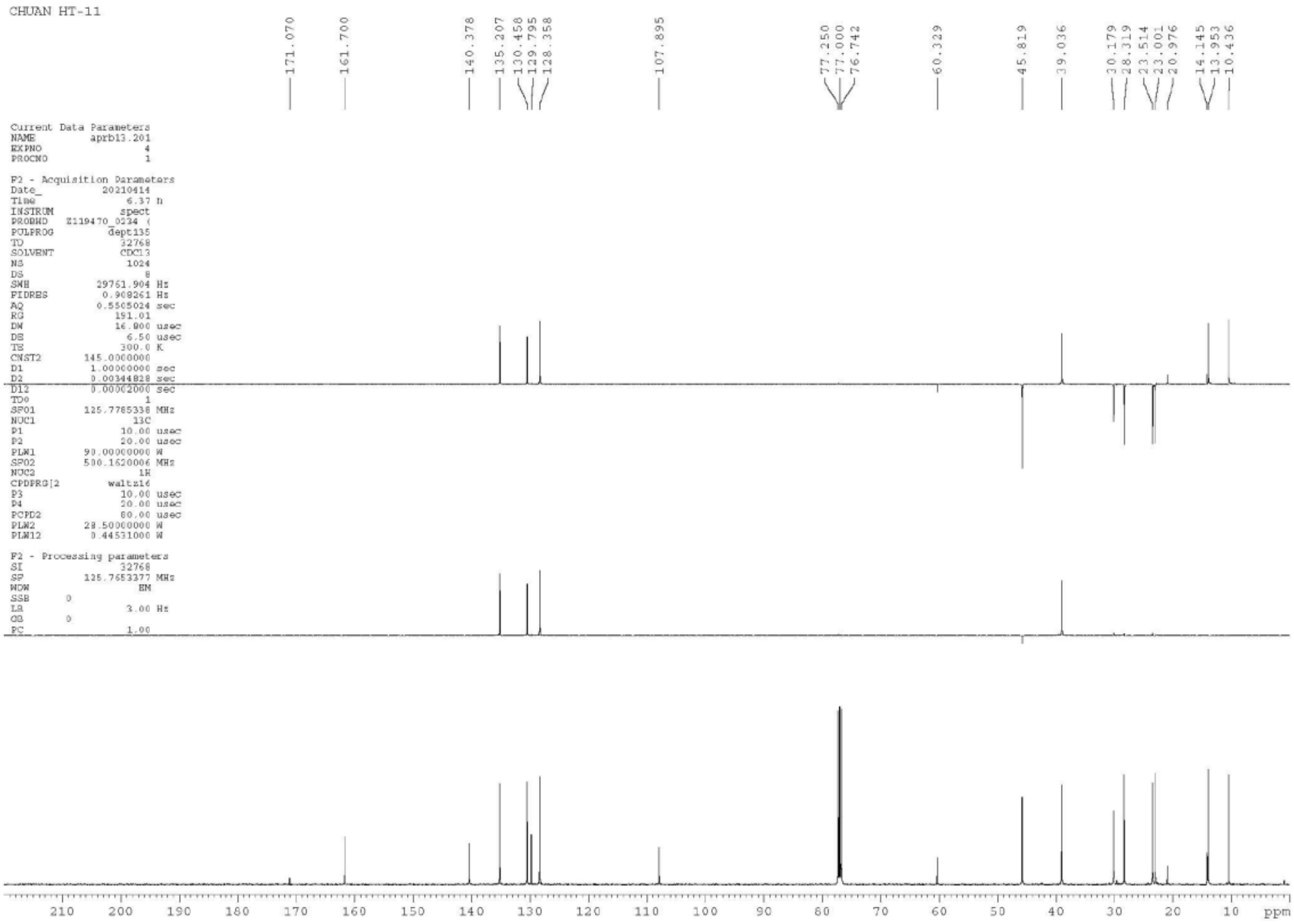

Figure S50: 4 — ^{13}C NMR (125 MHz, $CDCl_3$).

Display Report

Analysis Info

Acquisition Date 12/27/2024 3:24:46 PM

Analysis Name D:\Data\nctu service\data\2024\20241227\CHUAN HT-10_GD3_01_45382.d
Method Small molecule 200-1500.m
Operator NCTU
Sample Name CHUAN HT-10
Instrument impact HD 1819696.00164
Comment

Acquisition Parameter

Source Type	ESI	Ion Polarity	Positive	Set Nebulizer	1.0 Bar
Focus	Active	Set Capillary	4500 V	Set Dry Heater	200 °C
Scan Begin	200 m/z	Set End Plate Offset	-500 V	Set Dry Gas	6.0 l/min
Scan End	1500 m/z	Set Charging Voltage	2000 V	Set Divert Valve	Waste
		Set Corona	0 nA	Set APCI Heater	0 °C

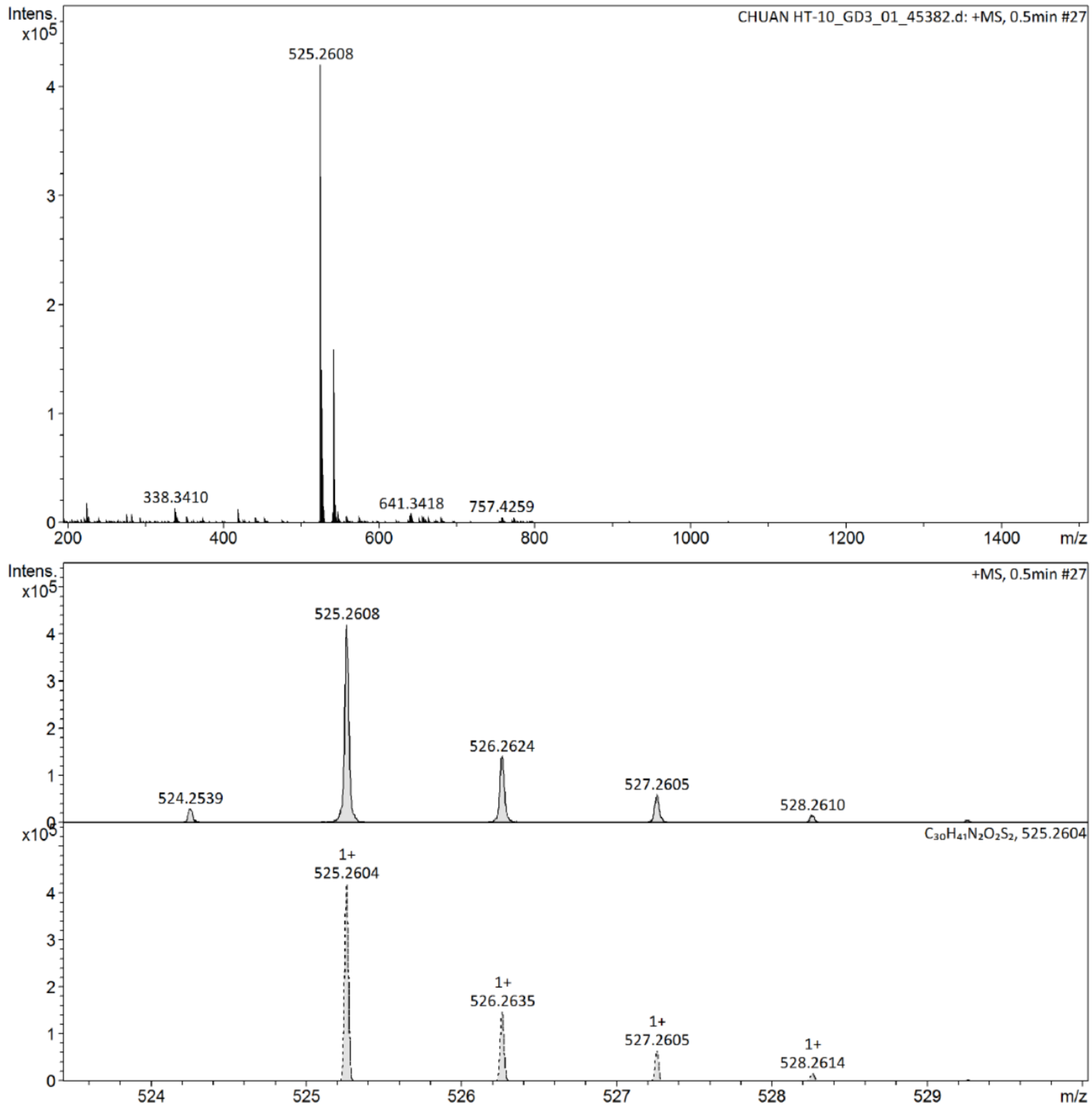

Figure S51: 4 — HRESIMS.

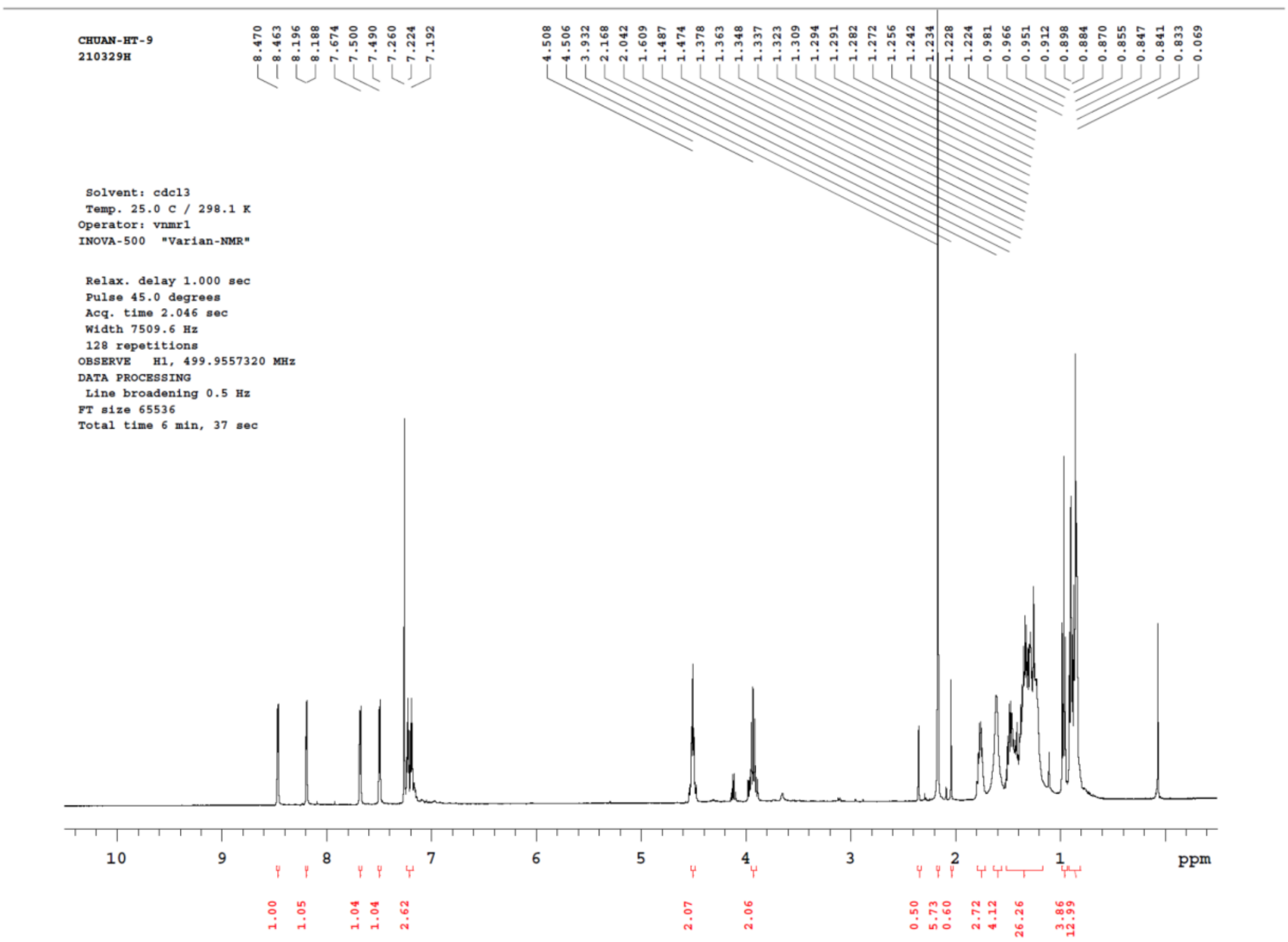

Figure S52: 4′ — ^{1}H NMR (500 MHz, $CDCl_3$).

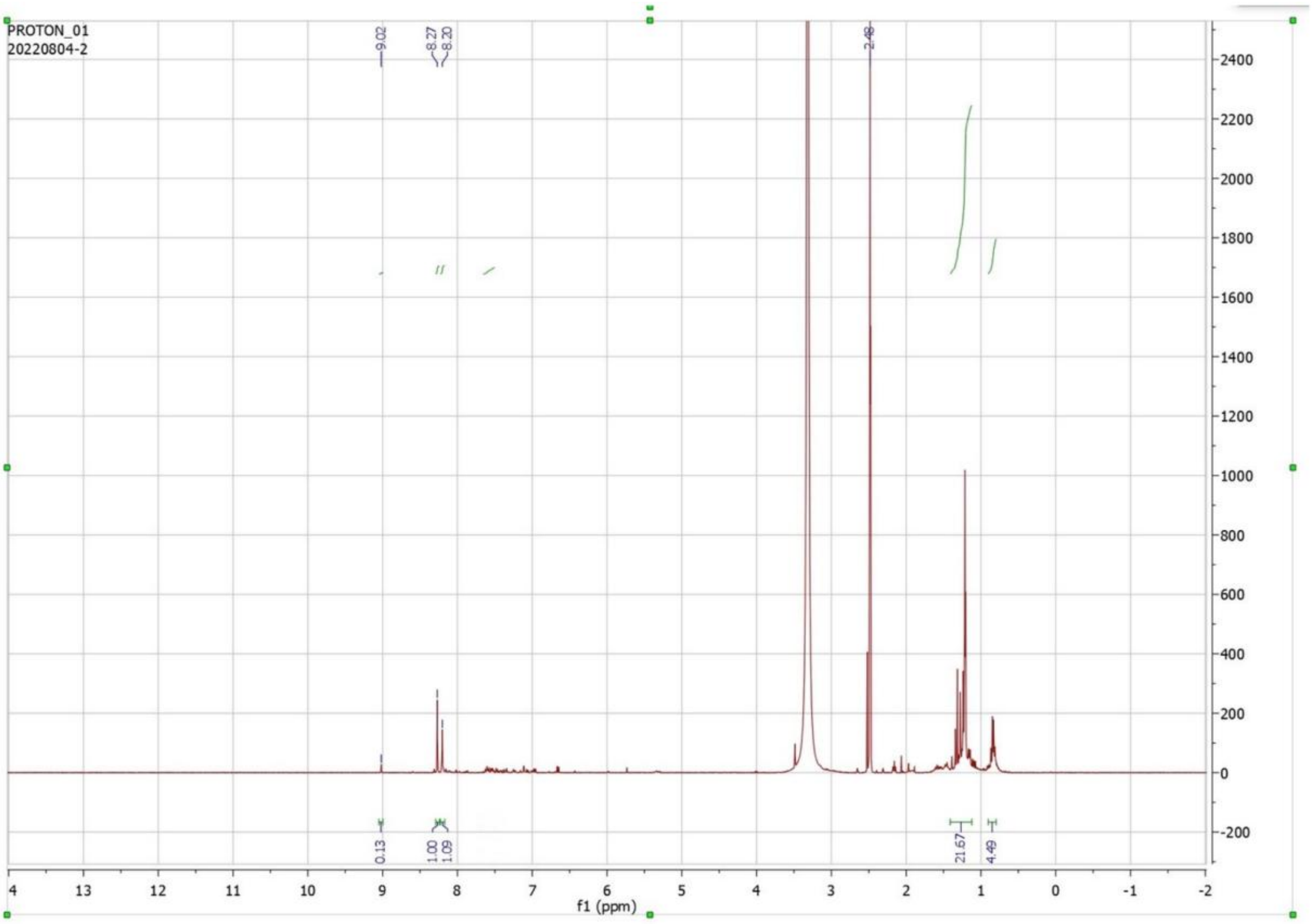

Figure S53: 15 — ^{1}H NMR (400 MHz, DMSO-d_6), recrystallized from MeOH.

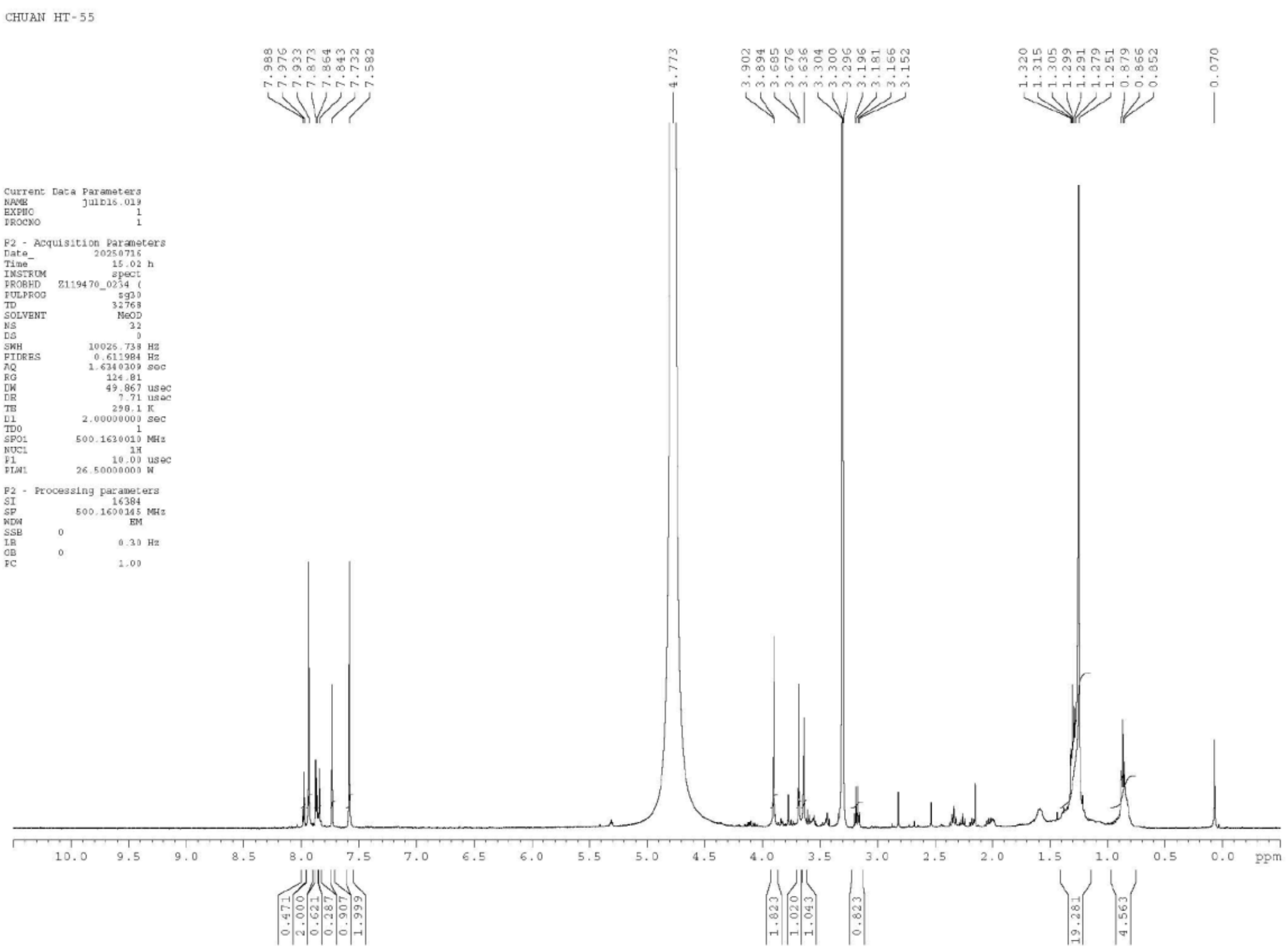

Figure S54: 15 — ^{1}H NMR (400 MHz, DMSO-d_6), after chromatography.

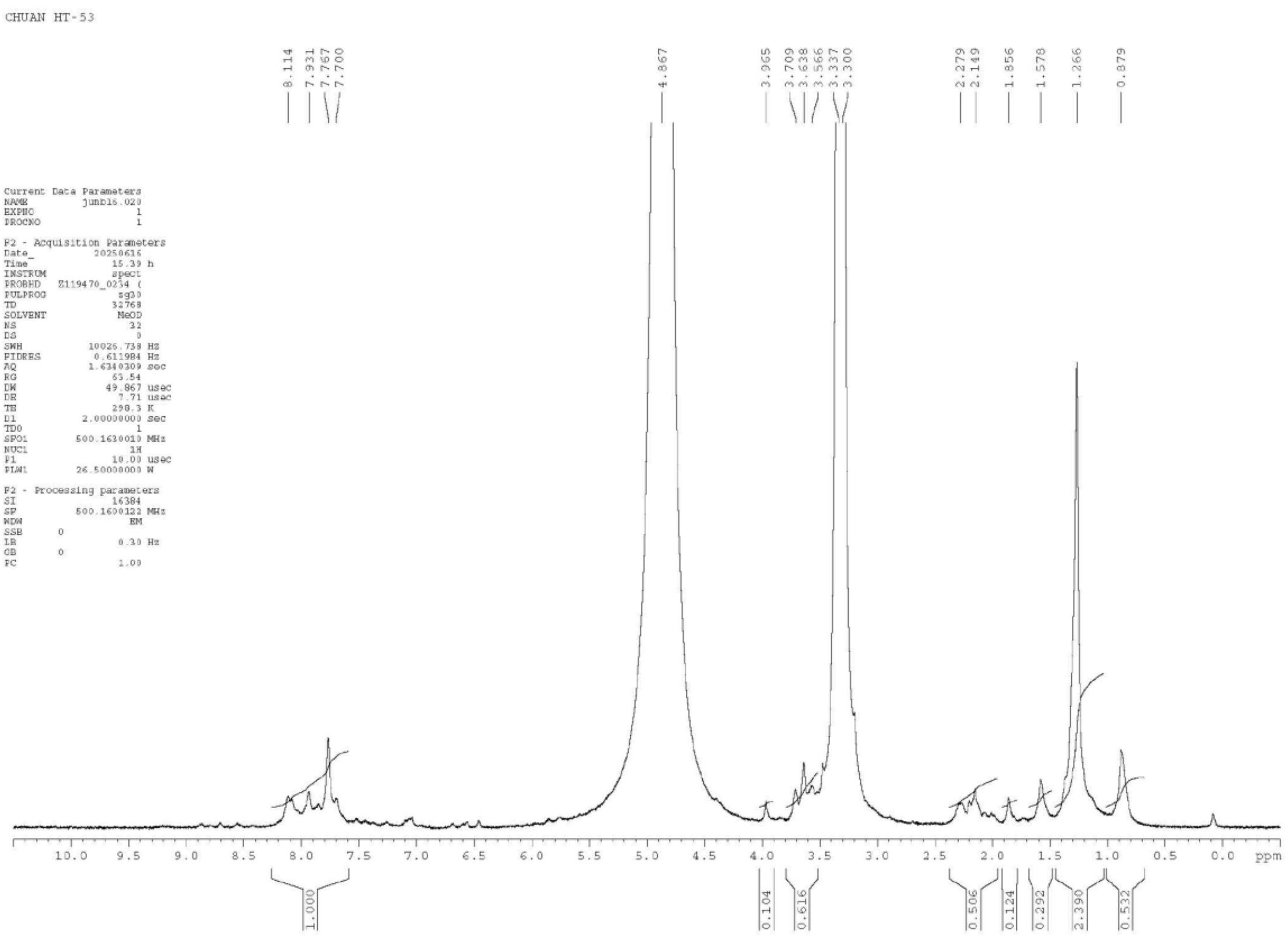

Figure S55: 17 (attempted) — ¹H NMR of the crude acidified reaction mixture.

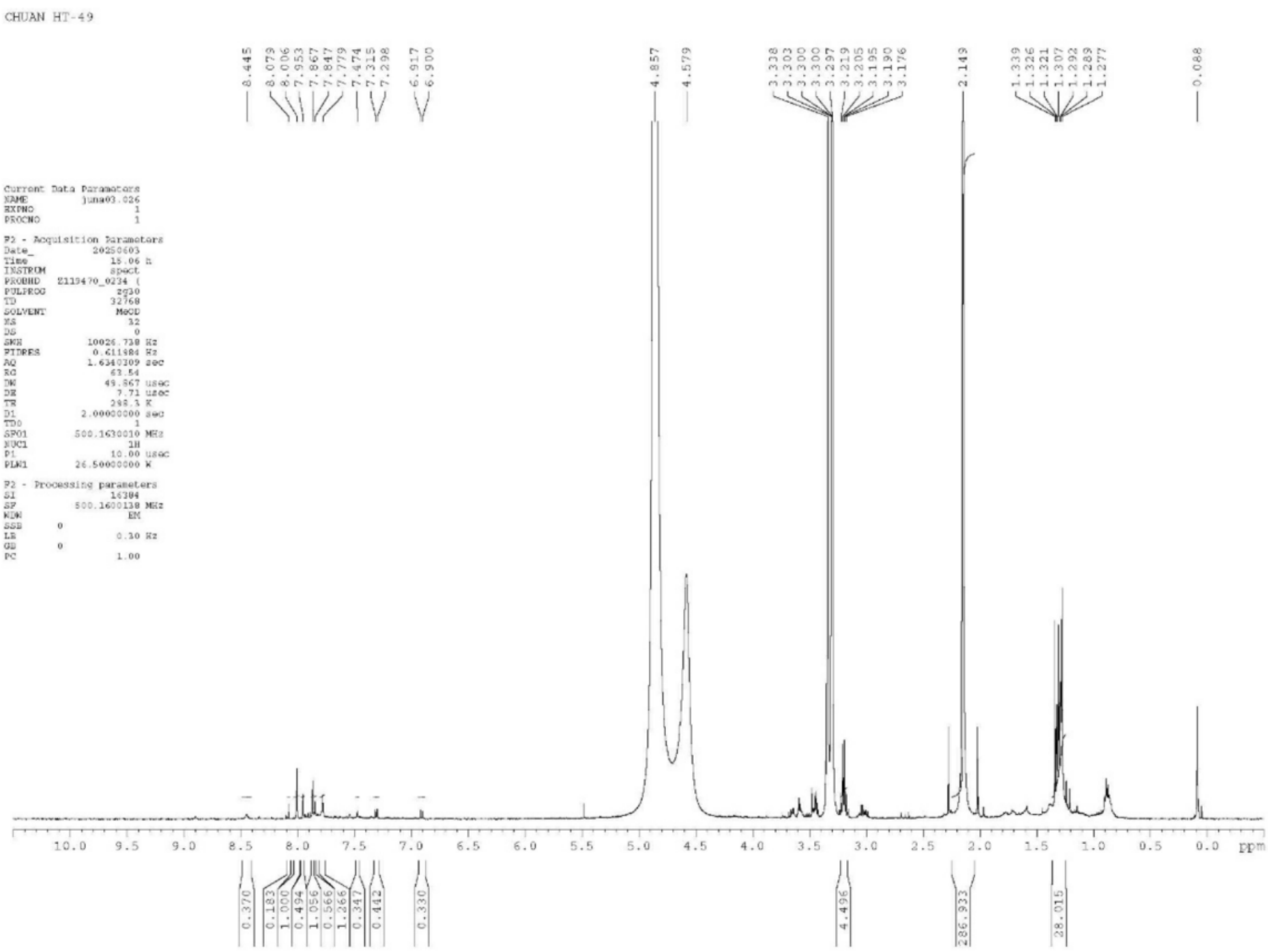

Figure S56: 17 (attempted) — ¹H NMR after flash chromatography.

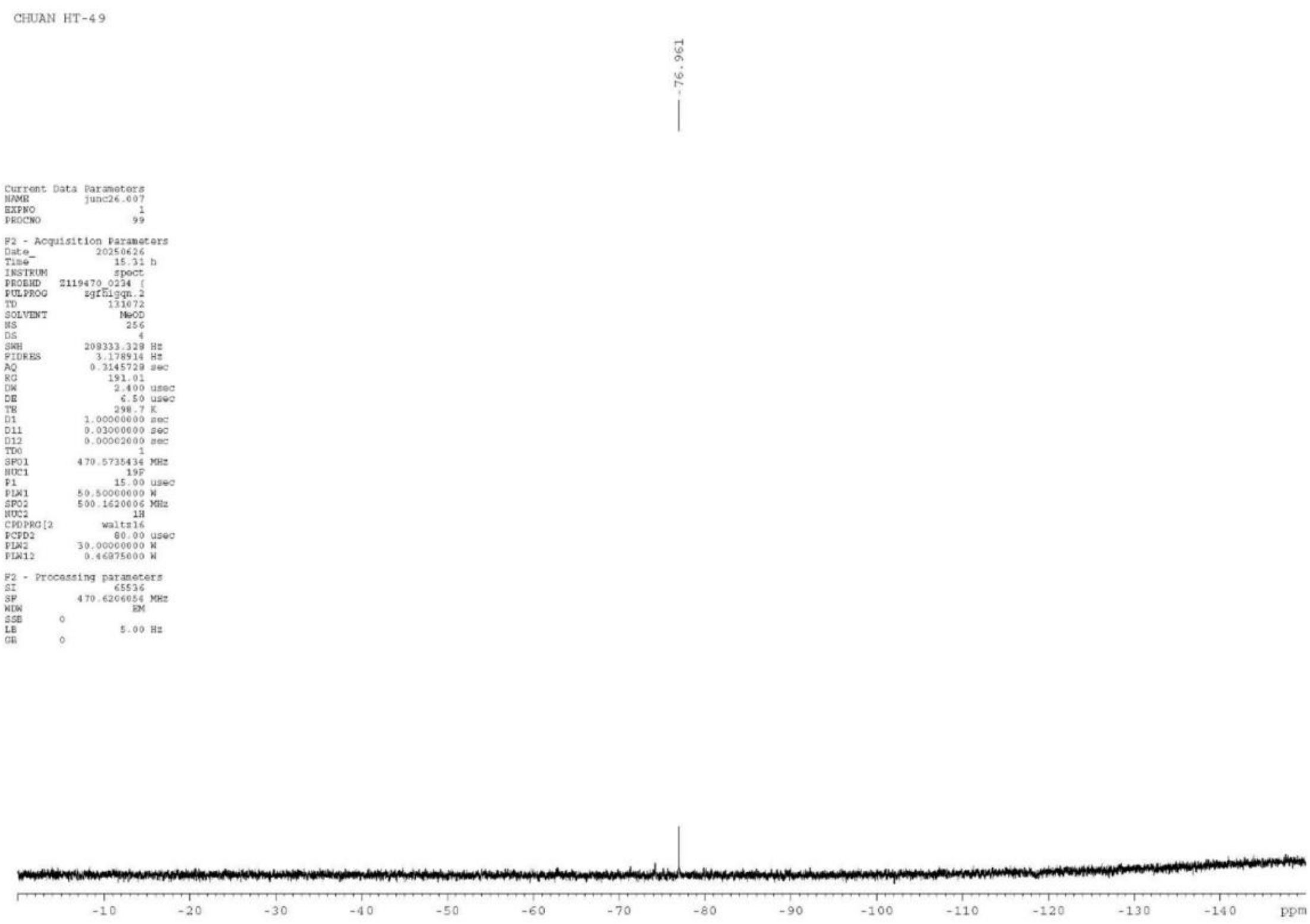

Figure S57: 17 (attempted) — ^{19}F NMR after flash chromatography, showing only the triflate singlet at δ −77.0.

Display Report

Analysis Info

		Acquisition Date	7/4/2025 4:57:10 PM
Analysis Name	D:\Data\nctu service\data\2025\new folder (2)\CHUAN HT-49_GE5_01_49119.d		
Method	Negative-MW100-1000.m	Operator	NCTU
Sample Name	CHUAN HT-49	Instrument	impact HD 1819696.00164
Comment			

Acquisition Parameter

Source Type	ESI	Ion Polarity	Negative	Set Nebulizer	1.0 Bar
Focus	Active	Set Capillary	4000 V	Set Dry Heater	200 °C
Scan Begin	50 m/z	Set End Plate Offset	-500 V	Set Dry Gas	6.0 l/min
Scan End	1500 m/z	Set Charging Voltage	2000 V	Set Divert Valve	Waste
		Set Corona	0 nA	Set APCI Heater	0 °C

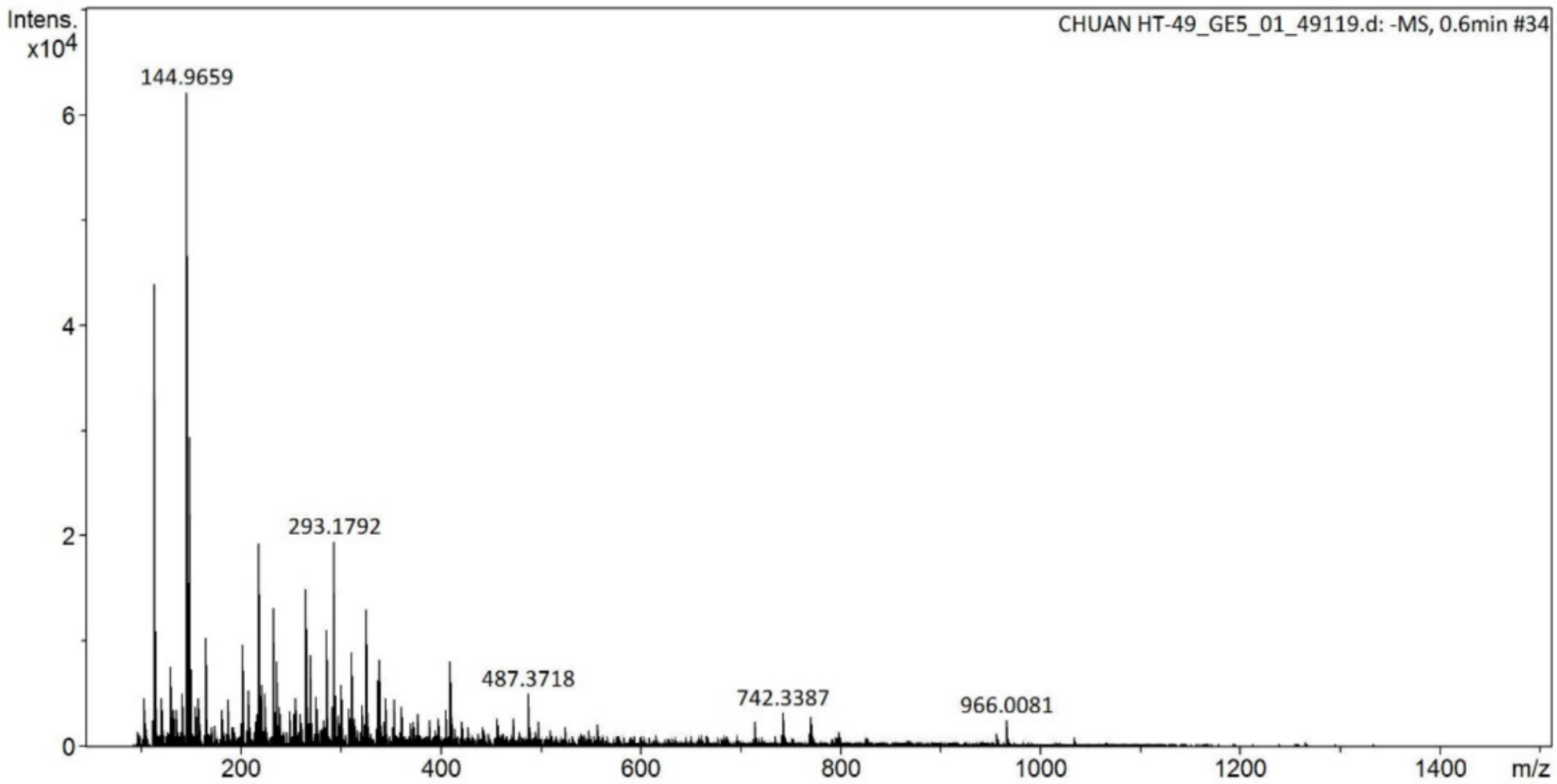

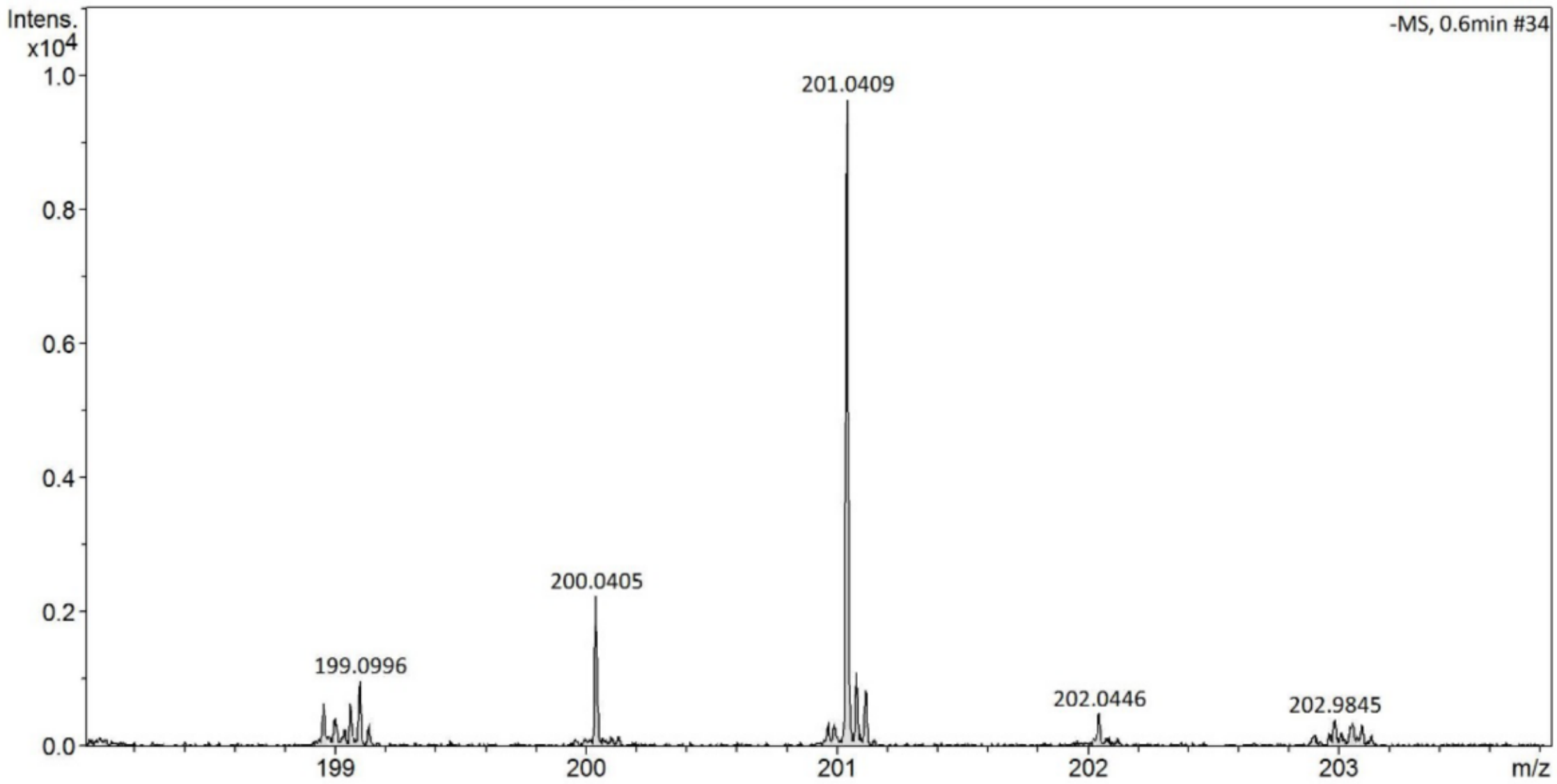

Figure S58: 17 (attempted) — HRESIMS after flash chromatography.

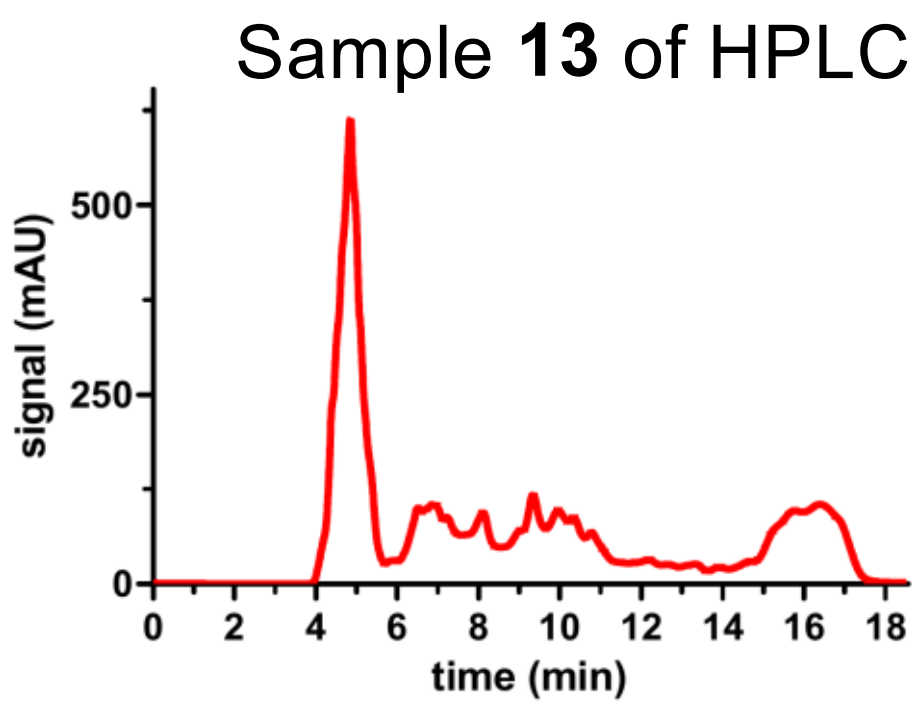

Figure S59: 17 (attempted) — reverse-phase HPLC trace (MeCN/H_2O 1:1, 366 nm).

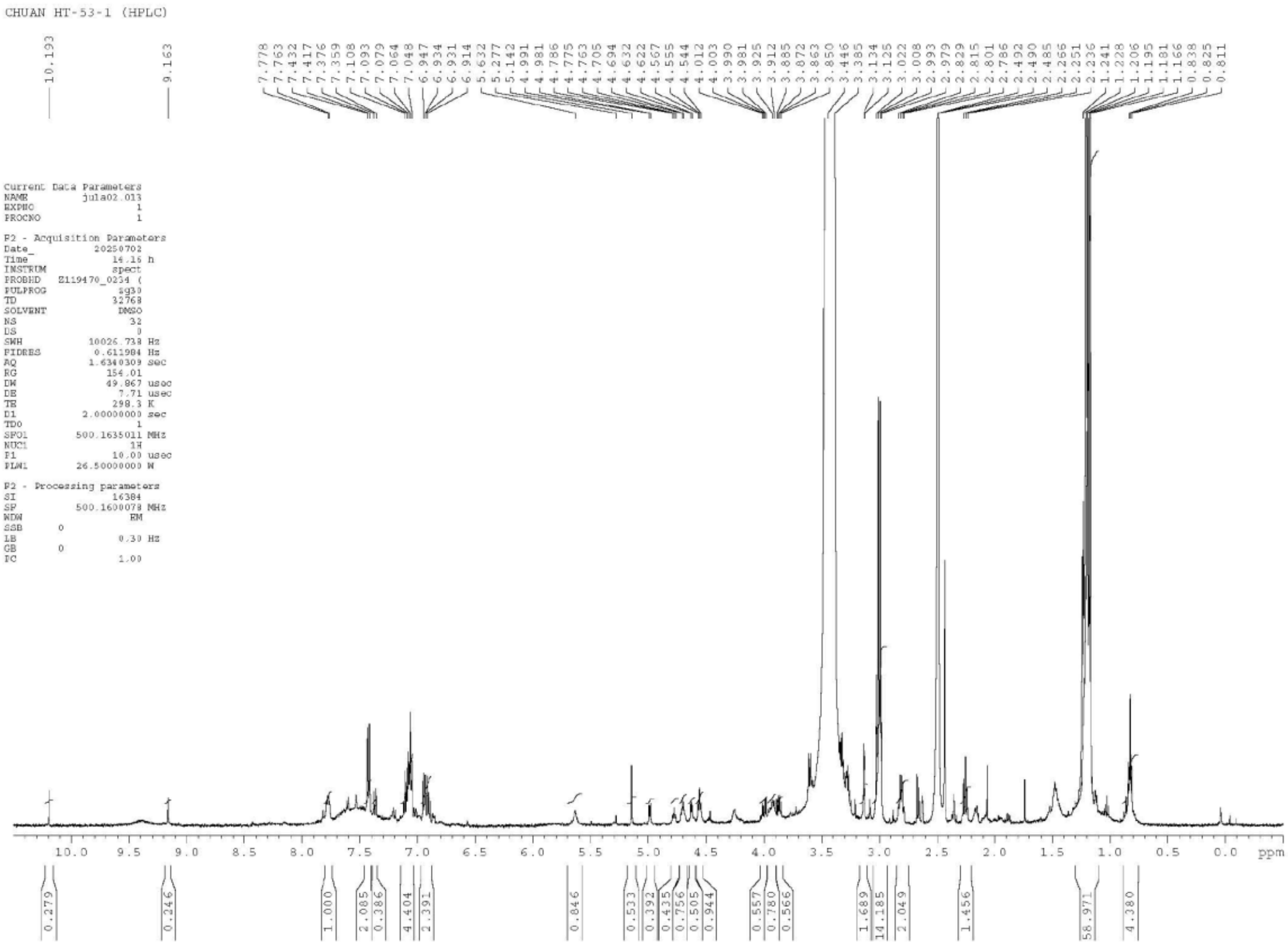

Figure S60: 17 (attempted) — ^{1}H NMR of HPLC fraction 14-1.

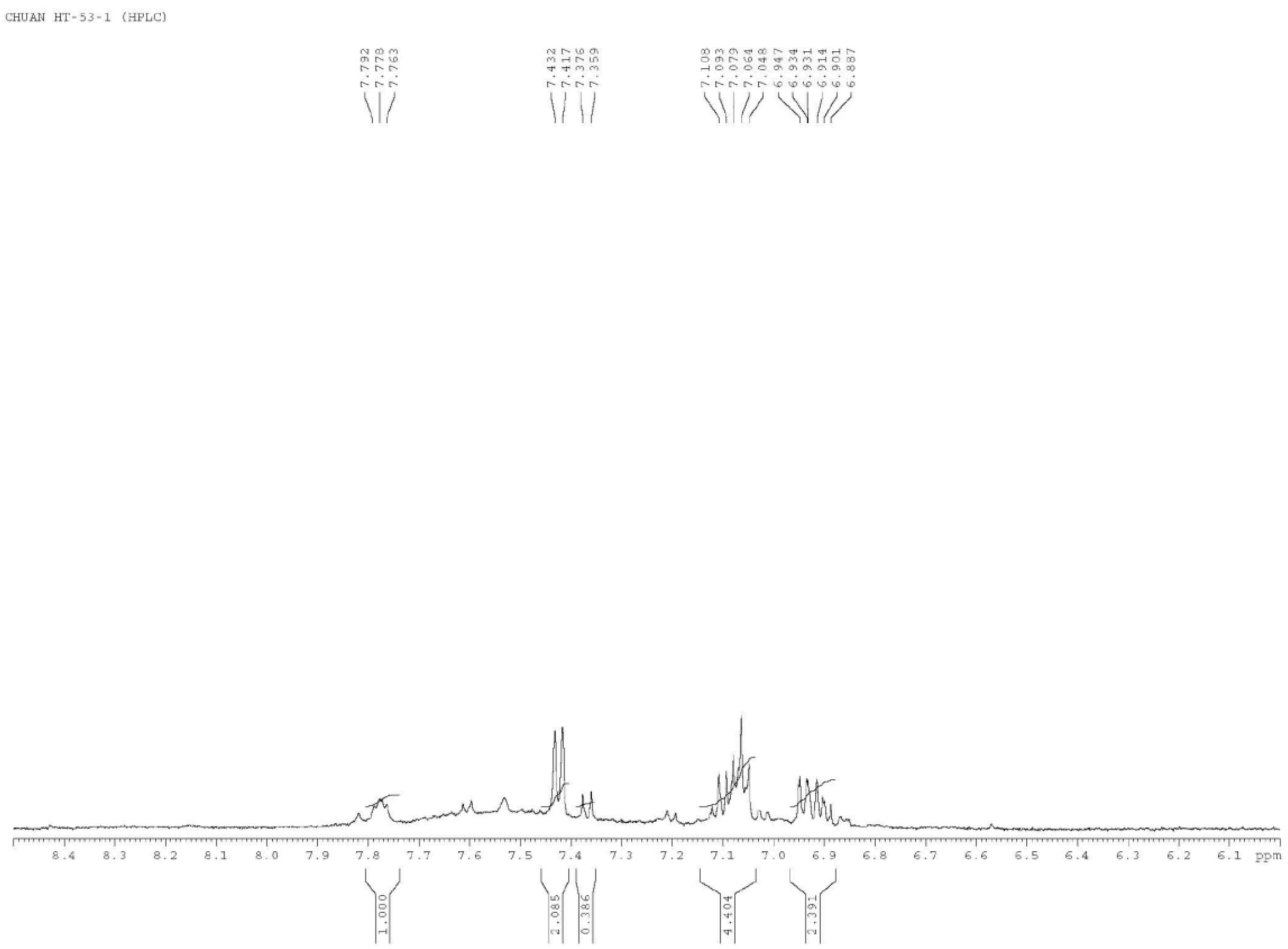

Figure S61: 17 (attempted) — ^{1}H NMR of HPLC fraction 14-1, aromatic region expanded.

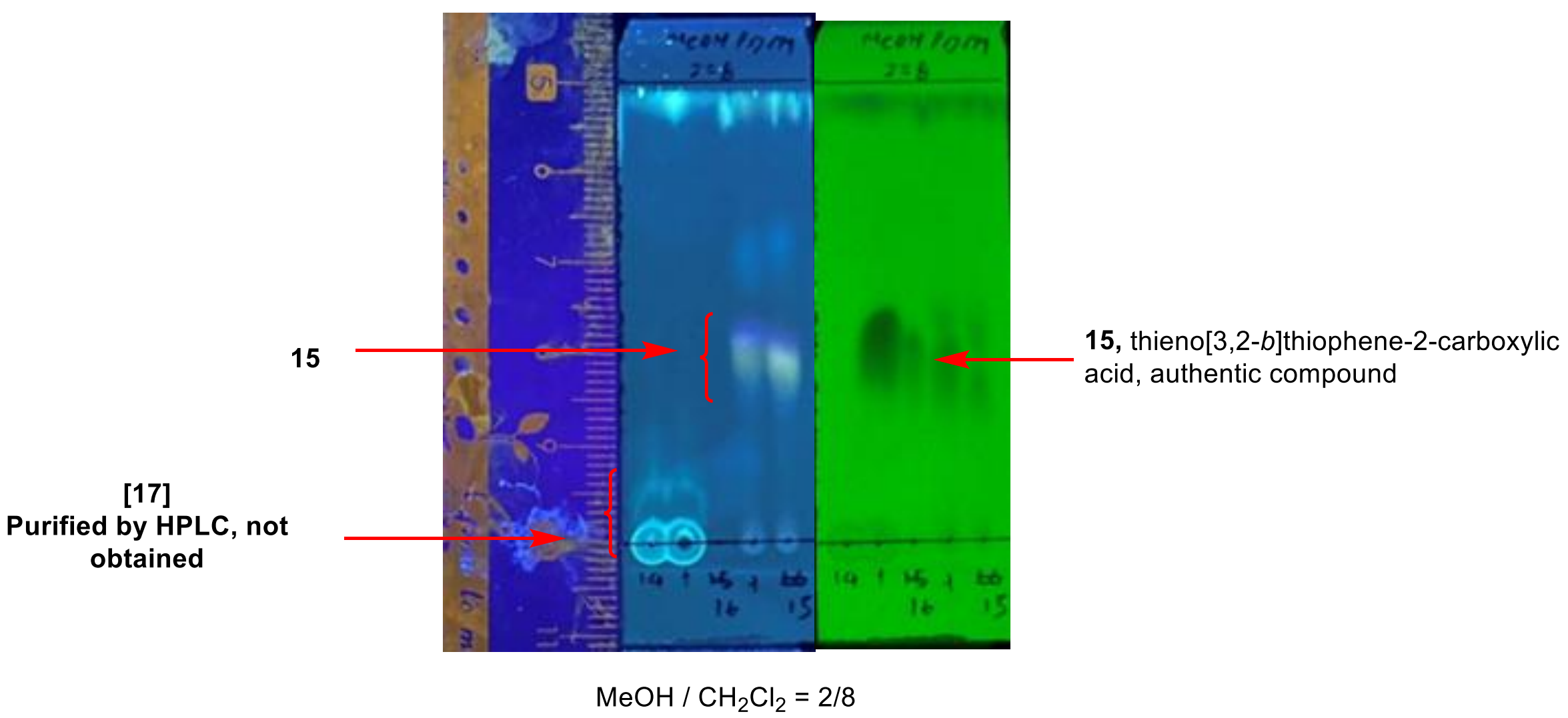

Figure S62: 17 (attempted), **15** and thieno[3,2-*b*]thiophene-2-carboxylic acid — TLC co-spotting comparison (MeOH/CH_2Cl_2 2:8). Compound **15** and thieno[3,2-*b*]thiophene-2-carboxylic acid are authentic samples prepared independently.

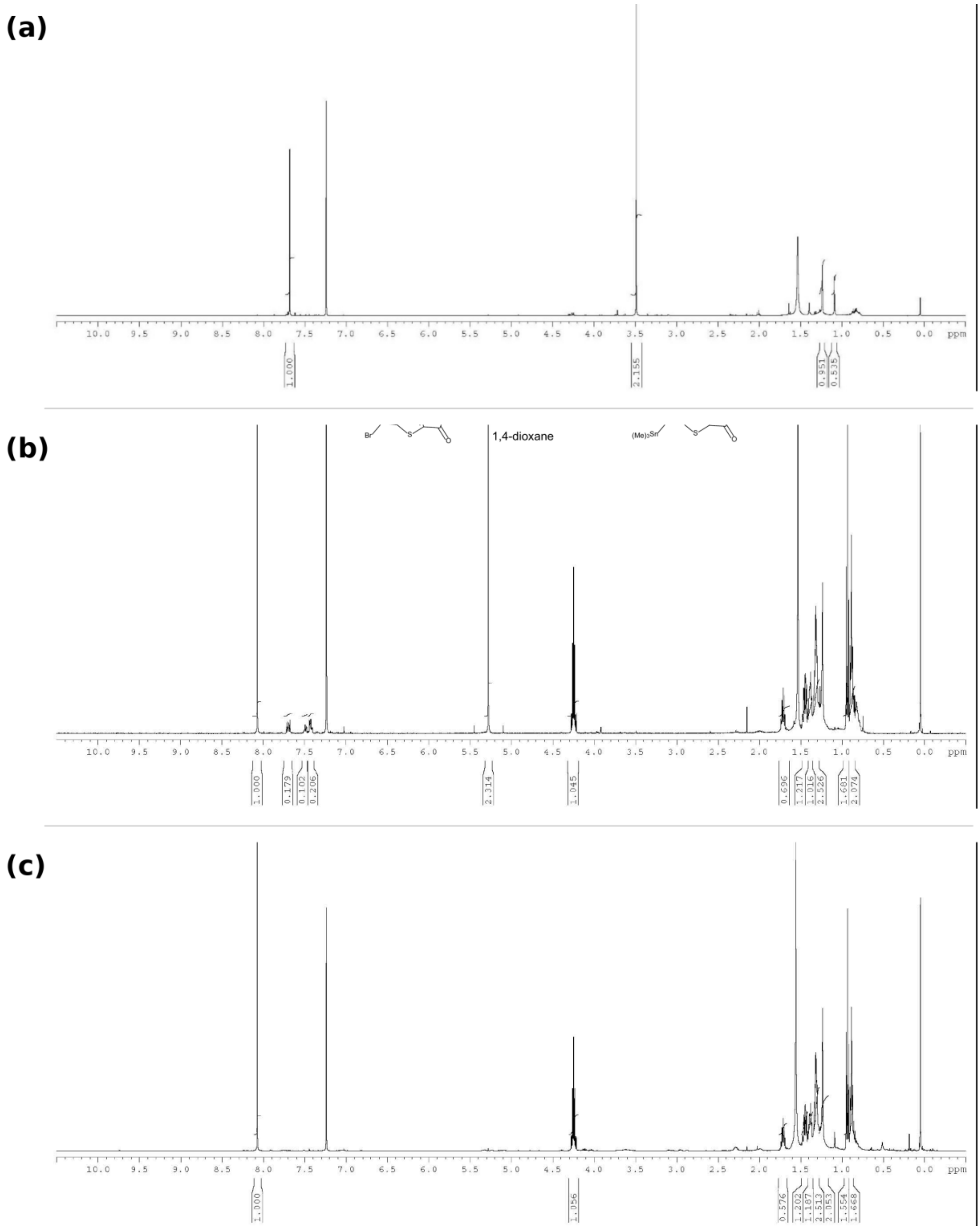

Figure S63: Stacked ^{1}H NMR spectra: (a) **10**; (b) crude product from $Me_6Sn_2/Pd(PPh_3)_2Cl_2$; (c) crude product from $Me_6Sn_2/Pd(PPh_3)_4$. The C6 methylene singlet of **10** near δ 3.5 is absent in (b) and (c).

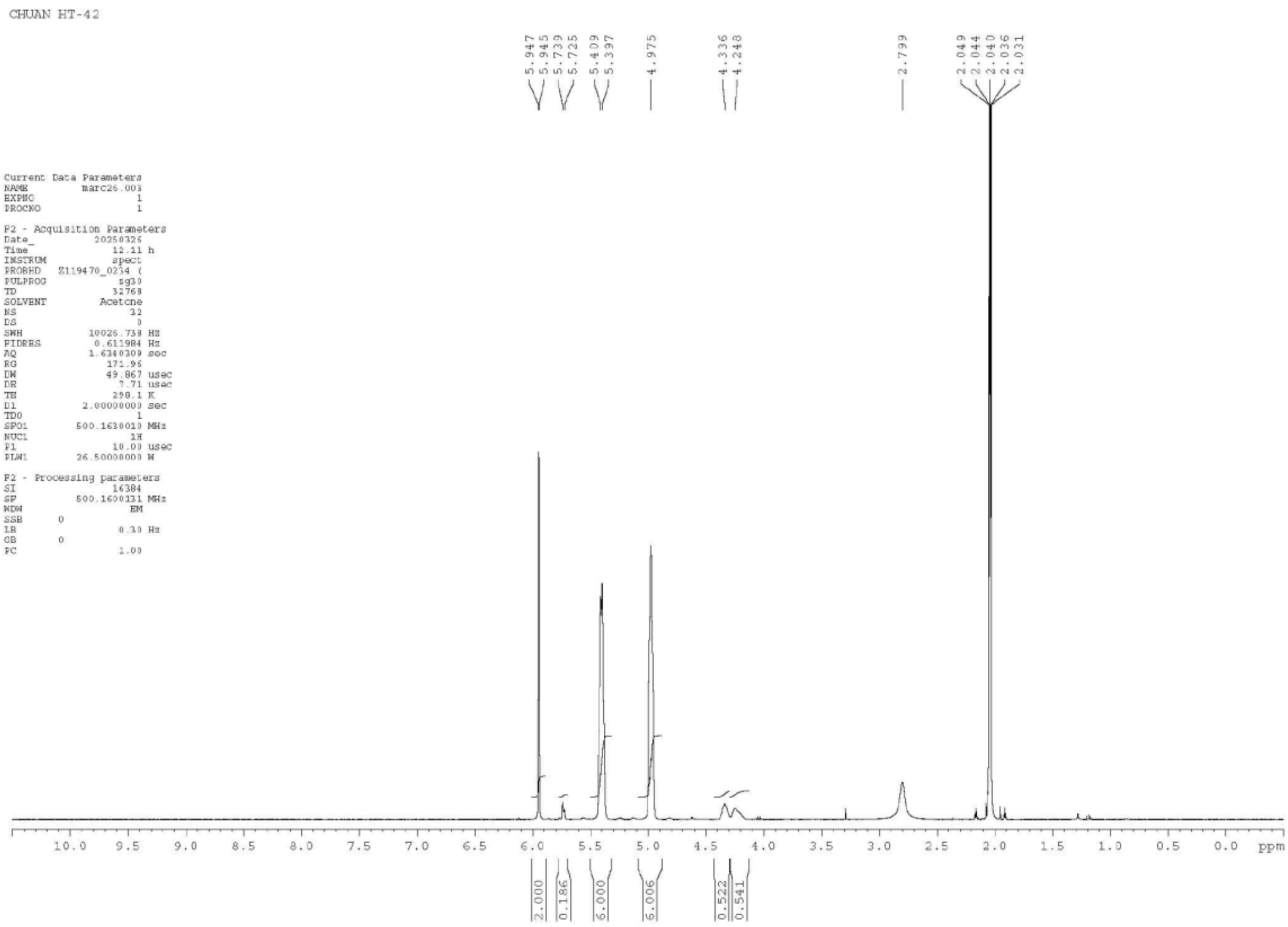

Figure S64: F-TEDA-PF_6 — ^1H NMR (acetone-d_6).

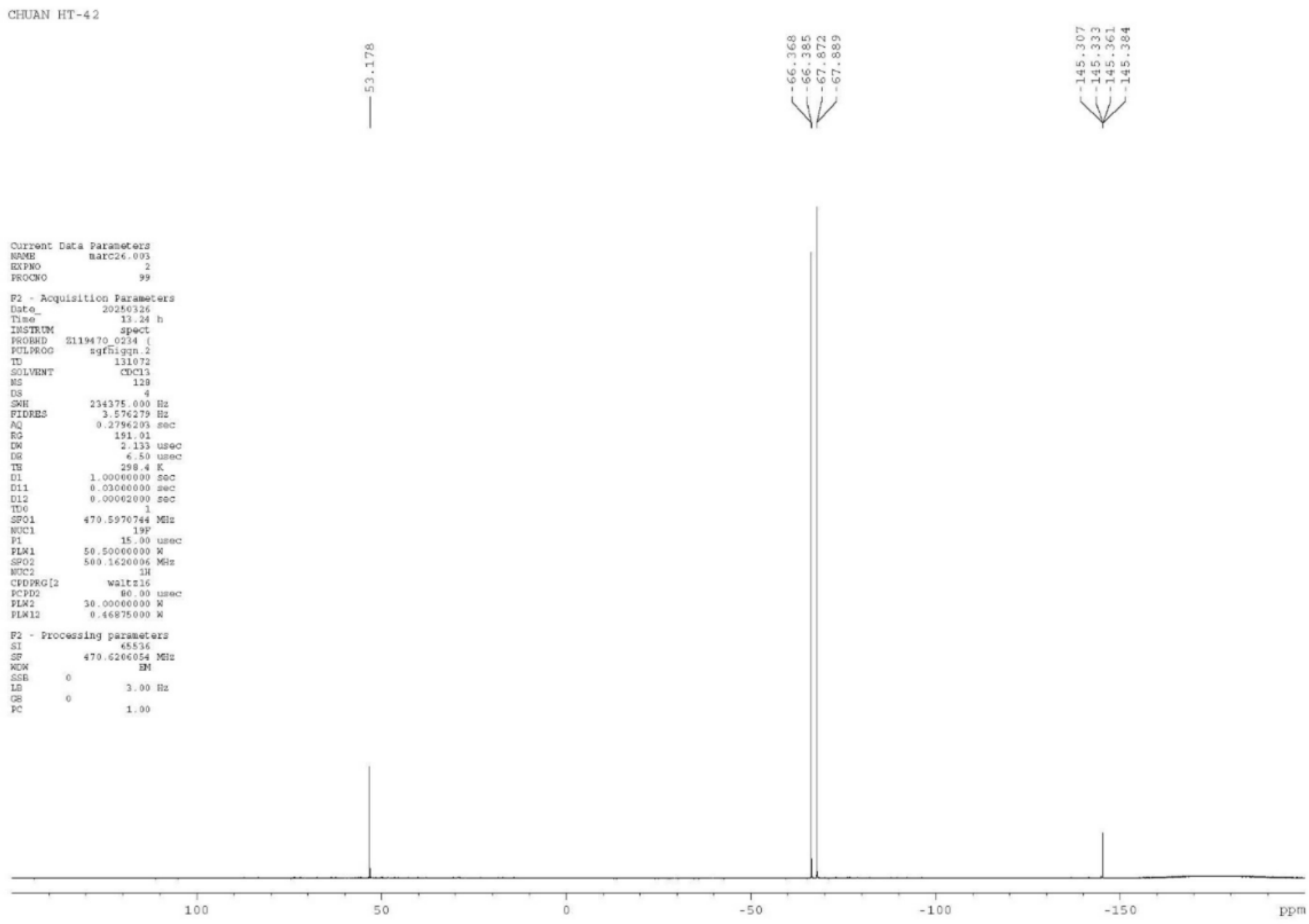

Figure S65: F-TEDA-PF_6 — ^{19}F NMR (acetone-d_6).

S9 References

S1. Sniekers, J.; Brooks, N. R.; Schaltin, S.; Van Meervelt, L.; Fransaer, J.; Binnemans, K. *Dalton Trans.* **2014**, *43*, 1589–1598.

S2. Sartori, G.; Bigi, F.; Maggi, R.; Baraldi, D.; Casnati, G. *J. Chem. Soc., Perkin Trans. 1* **1992**, 2985–2988.

S3. Li, S.; Ye, L.; Zhao, W.; Zhang, S.; Ade, H.; Hou, J. *Adv. Energy Mater.* **2017**, *7*, 1700183.

S4. Hodson, H. F.; Madge, D. J.; Slawin, A. N. Z.; Widdowson, D. A.; Williams, D. J. *Tetrahedron* **1994**, *50*, 1899–1906.

S5. Eldrup, A. B.; Prhavc, M.; Brooks, J.; Bhat, B.; Prakash, T. P.; Song, Q.; Bera, S.; Bhat, N.; Dande, P.; Cook, P. D.; Bennett, C. F.; Carroll, S. S.; Ball, R. G.; Bosserman, M.; Burlein, C.; Colwell, L. F.; Fay, J. F.; Flores, O. A.; Getty, K.; LaFemina, R. L.; Leone, J.; MacCoss, M.; McMasters, D. R.; Tomassini, J. E.; Von Langen, D.; Wolanski, B.; Olsen, D. B. *J. Med. Chem.* **2004**, *47*, 5284–5297.

S6. Furuya, T.; Strom, A. E.; Ritter, T. *J. Am. Chem. Soc.* **2009**, *131*, 1662–1663.

S7. Tang, P.; Furuya, T.; Ritter, T. *J. Am. Chem. Soc.* **2010**, *132*, 12150–12154.

S8. Ye, Y.; Sanford, M. S. *J. Am. Chem. Soc.* **2013**, *135*, 4648–4651.

S9. Makaravage, K. J.; Brooks, A. F.; Mossine, A. V.; Sanford, M. S.; Scott, P. J. H. *Org. Lett.* **2016**, *18*, 5440–5443.

S10. Gamache, R. F.; Waldmann, C.; Murphy, J. M. *Org. Lett.* **2016**, *18*, 4522–4525.

S11. Ye, Y.; Schimler, S. D.; Hanley, P. S.; Sanford, M. S. *J. Am. Chem. Soc.* **2013**, *135*, 16292–16295.

S12. Hwang, H.; Park, C.; Sin, D. H.; Song, E.; Cho, K. *Org. Electron.* **2020**, *83*, 105769.

S13. Borshchev, O. V.; Skorotetcky, M. S.; Trukhanov, V. A.; Fedorenko, R. S.; Surin, N. M.; Svidchenko, E. A.; Sosorev, A. Y.; Kazantsev, M. S.; Paraschuk, D. Y.; Ponomarenko, S. A. *Dyes Pigm.* **2021**, *185*, 108911.

S14. Iqbal, A.; Jost, M.; Kirchmayr, R.; Pfenninger, J.; Rochat, A.; Wallquist, O. *Bull. Soc. Chim. Belg.* **1988**, *97*, 615–643.

S15. Rochat, A. C.; Cassar, L.; Iqbal, A. Preparation of pyrrolo[3,4-c]pyrroles. European Patent 0094911 B1, November 23, 1983.

S16. Sonar, P.; Singh, S. P.; Soh, M. S.; Li, Y. Ambipolar polymeric semiconductor materials and organic electronic devices. Taiwan Patent I508991 B, November 21, 2015.

S17. Pop, F.; Humphreys, J.; Schwarz, J.; Brown, L.; van den Berg, A.; Amabilino, D. B. *New J. Chem.* **2019**, *43*, 5783–5790.

S18. Pomerantz, M.; Turkman, N. *Synthesis* **2008**, 2333–2336.

S19. El Kassmi, A.; Fache, F.; Lemaire, M. *Synth. Commun.* **1994**, *24*, 95–101.
